# DirectContacts2: a wiring diagram of human physical protein interactions

**DOI:** 10.1038/s41467-026-75863-3

**Published:** 2026-07-24

**Authors:** Erin R. Claussen, Miles D. Woodcock-Girard, Samantha N. Fischer, Kevin Drew

**Affiliations:** Department of Biological Sciences, University of Illinois at Chicago, Chicago, IL USA

**Keywords:** Molecular modelling, Protein structure predictions, Protein-protein interaction networks

## Abstract

Cellular function is driven by the activity of proteins in stable complexes. Protein complex assembly depends on the direct physical association of component proteins. Advances in macromolecular structure prediction with tools like AlphaFold and RoseTTAFold have greatly improved our ability to model these interactions in silico, but an all-by-all analysis of the human proteome’s ~200 M possible pairs remains computationally intractable. A comprehensive cellular map of direct protein interactions will therefore be an invaluable resource to direct screening efforts. Here, we present *DirectContacts2*, a machine learning model that distinguishes direct from indirect protein interactions using features derived from over 25,000 mass spectrometry experiments. Applied to ~25 million human protein pairs, our model outperforms previous resources in identifying direct physical interactions and enriches for accurate structural models including ~2500 AlphaFold3 models. Our framework enables structural modeling of disease-relevant complexes (e.g. orofacial digital syndrome (OFDS) complex) offering insights into the molecular consequences of pathogenic mutations (OFD1) and broadly, establishes a highly accurate protein wiring diagram of the cell.

## Abstract (web summary)

Knowledge of the physical interactions of proteins provides mechanistic understanding of their function. Here, the authors develop a machine learning classifier through the integration of 25,000 proteomics experiments to construct a wiring diagram of human cells.

## Introduction

**Fig. 1:**
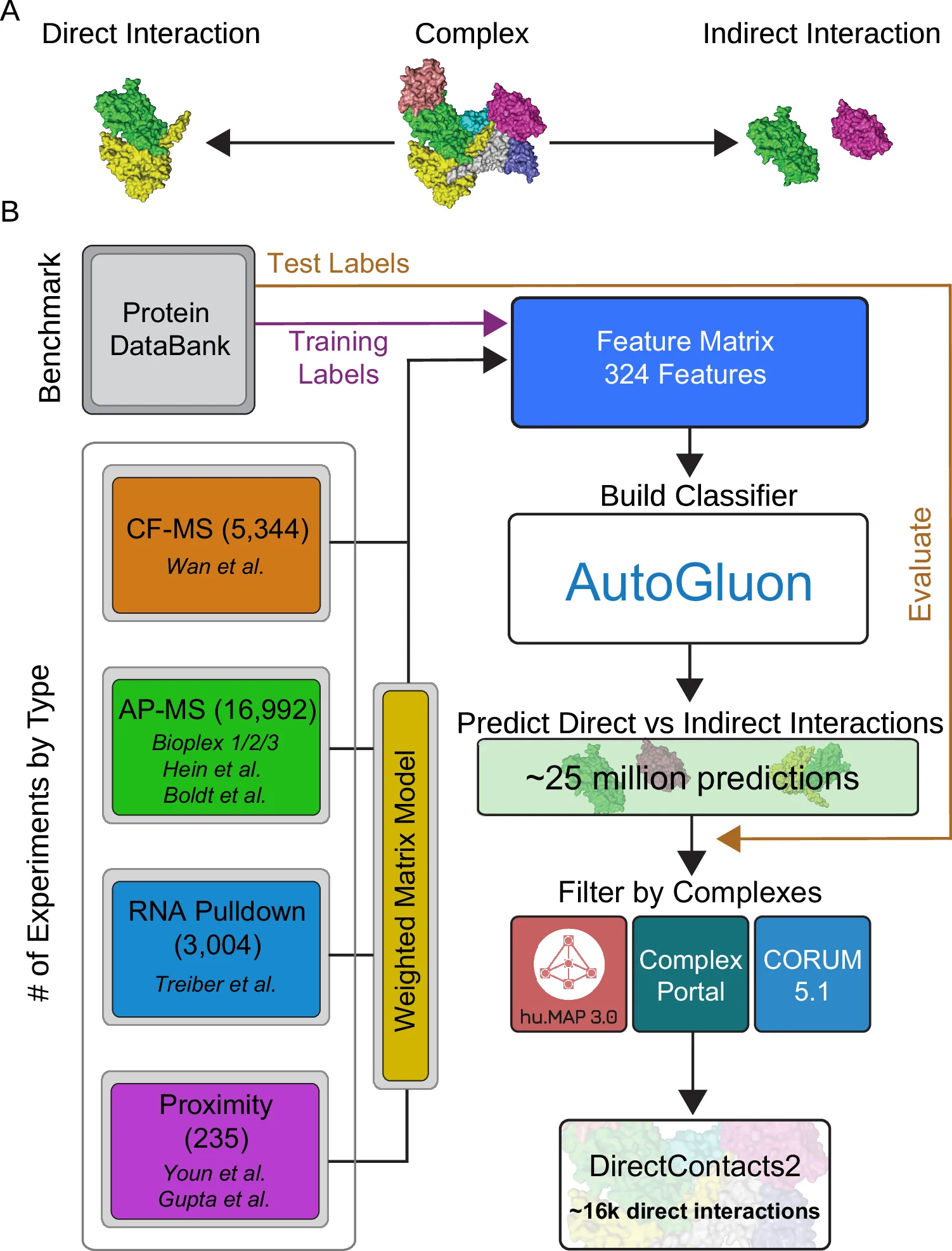
Classification of direct and indirect protein interactions across the human proteome. **A** Image displays an illustrative, multi-subunit protein complex where some directly interact (yellow and green subunits) while others indirectly interact through other subunits (magenta and green subunits). **B** Workflow from our machine learning pipeline to integrate high-throughput proteomics data to discriminate between direct and indirect protein interactions. Data from multiple sources were used to train a machine learning model, with the training set including 1472 positives and 47,246 negatives. The leave-out test set evaluation is performed on two sets: (1) 572 positives and 785 negatives, and (2) 572 positives and 25,717 negatives (see “Methods”). The model is then applied to ~25 million human protein pairs. The final network represents high-confidence direct interactions filtered by confidence and previously identified protein complexes. Adapted from ref. ^28^.

The self-assembly of proteins into protein complexes is critical for their biological function^1^. Disruptions in these protein complexes often give rise to human diseases, including cancer^2^, developmental disorders^3^, and protein folding disorders, such as Parkinson’s disease^4^. Knowledge of each direct physical interaction of protein subunits (Fig. 1A) ultimately gives rise to the wiring diagram of the cell. This diagram is essential for understanding the molecular mechanism of protein complex function, including why protein complex function fails in pathological states. It also aids in drug target discovery^5^ and constructing three-dimensional models of protein complexes on the proteome-wide scale.

Recent advances in computational protein structure prediction now allow for near-atomic structural modeling of proteins and protein complexes using sequence alone^6–9^. AlphaFold2 and RosettaFold have been applied to predict structures of protein-protein interactions across *E.coli*, yeast, human, and other proteomes^10–16^. However, current limitations in computational resources prevent scaling these methods to all pairs in the proteome (e.g., 200 million theoretical protein pairs in the human interactome). Non-negligible false positive rates of structural modeling algorithms also limit scalability. To limit the search space of true interactors, Burke et al. applied AlphaFold2 to high-confidence predictions from hu.MAP2.0^17^, a network of co-complex interactions trained on high-throughput mass spectrometry data, showed an enrichment of high-scoring structure predictions over random pairs^13^. Interestingly, while hu.MAP2.0 showed enrichment for high-confidence structural models, the hu.MAP2.0 model was originally designed to classify pairs that co-complex, regardless of whether or not they make a contacting interface. We reasoned that a machine learning model designed specifically to differentiate direct from indirect protein interactions will generate an accurate network of directly interacting pairs and ultimately enrich for high-confidence computationally derived protein interaction structural models.

Here, we aim to identify direct physical interactions among proteins in human cells. Using data from over 25,000 mass spectrometry experiments, we developed DirectContacts2, a machine learning classifier that discriminates between direct and indirect protein interactions in protein complexes. In previous work, we showed that thousands of co-fractionation mass spectrometry (CF-MS) experiments could aid in the identification of physical protein contacts^18,19^. Here we drastically improve on prior efforts in terms of accuracy and scale, by incorporating CF-MS data with affinity purification mass spectrometry (AP-MS)^20–24^, RNA pulldown experiments^25^, and a reanalysis of proximity labeling experiments^26,27^ to extract proteomic indicators of physical interactions. We built a machine learning classifier to integrate these features into a single probabilistic score, which we then applied to ~25 million human protein pairs. We show that our results outperform our previous DirectContacts^19^ model, hu.MAP2.0^17^, hu.MAP3.0^28^, and the HuRI reference map of human binary protein interactions^29^ on a benchmark of Protein Data Bank (PDB)-curated direct protein interactions^30,31^. In addition, we show our network is complementary to sequence-ML- based methods, such as RoseTTAFold2-PPI^15^. We then show our results boost the ability of identifying highly accurate AlphaFold2/AlphaFold-Multimer predictions when applying these structure prediction tools across the human proteome, and we extend this result to ~2500 AlphaFold3 models. We demonstrate the utility of DirectContacts2 predictions to guide structural model predictions for disease-associated complexes and place human disease mutations in structural context using a case study of a complex implicated in ciliopathy. Finally, we systematically evaluate the evolution of direct interactions with respect to their co-complexing proteins.

## Results

To construct our network of direct protein-protein interactions, we integrated 25,575 publicly available mass spectrometry experiments from high-throughput proteomic efforts using a machine learning framework trained on experimentally derived direct interactions found in the PDB. Our approach integrates orthogonal experimental datatypes, a strategy that has been successful in the past for building co-complex protein interaction networks, such as hu.MAP^17,28,32^. Here, we integrate co-fractionation mass spectrometry (CF-MS) data from Wan et al.^17^, AP-MS data from Bioplex 1,2,3^20–22^, Boldt et al.^24^, Hein et al.^23^, and other high-throughput proteomics datasets^25–27^. CF-MS experiments involve the biochemical separation of native protein complexes followed by mass spectrometry identification of proteins in collected fractions. We have shown previously^18,19^ that proteins in direct physical contact are more likely to coelute in a CF-MS experiment. These direct interactions can therefore be discriminated from indirectly interacting proteins by the higher similarity coefficient (e.g., Pearson correlation) of their elution profiles. In total, we construct features from 5344 CF-MS fractions (Fig. 1B), comparing nearly 1 million protein pairs (Supplementary Fig. 1A), and covering 10,396 proteins (Supplementary Fig. 1B).

AP-MS experiments involve the isolation of native protein complexes, followed by a series of washes, and then identification by mass spectrometry. We therefore expect proteins that physically interact with the bait protein to show higher abundance in AP-MS experiments than indirectly interacting proteins. In addition to evaluating AP-MS experiments individually, we previously showed that analyzing AP-MS data collectively, using a weighted matrix model (WMM), improves co-complex interaction prediction^17,28,32^. We implement the WMM by applying the hypergeometric test to a pair of proteins across the many thousands of AP-MS experiments. In essence, the WMM asks how often a pair of proteins are seen together in the same set of experiments and whether they are seen more often than random chance. In this application, we expect pairs seen in the same set of experiments far more than random chance to have a higher likelihood to directly interact, thus allowing us to discriminate between direct and indirect interactions. This is likely due to directly interacting proteins being pulled down together in more experiments than proteins that are indirectly interacting. In total, our model incorporates data from 16,992 AP-MS experiments (Fig. 1B), covering >22 million protein pairs (Supplementary Fig. 1A) and 17,100 proteins (Supplementary Fig. 1B).

In addition to AP-MS, we also incorporate WMM features derived from proximity labeling and RNA pulldown experiments into our model. Each individual experiment is expected to have a limited signal for direct interactions. However, when we apply the WMM method to these datasets, they do provide statistical evidence for direct interactions (see section “Signal from Individual Features”). Specifically, we applied the WMM to 235 proximity labeling experiments^26,27^ and 3004 RNA Pulldown experiments^25^ (Fig. 1B). In total, we integrate 25,575 mass spectrometry experiments, encompassing data for ~26 million protein pairs (Supplementary Fig. 1A) and 17,233 proteins (Supplementary Fig. 1B), into a machine learning framework to build a classifier that discriminates between direct and indirect protein interactions.

### Integration of proteomics data improves discrimination between direct and indirect interactions

To build a network of directly interacting proteins, we reasoned that the integration of many orthogonal features derived from mass spectrometry experiments will increase our ability to discriminate between direct and indirect protein interactions. We first constructed a benchmark of direct and indirect protein interactions based on three-dimensional structures in the Protein DataBank^30^. Due to concerns of data leakage in predicting protein interactions^33,34^, we split complexes from the PDB into training and test sets using a greedy hierarchical clustering approach (see “Methods”). Our benchmark clustering approach ensures no overlap of complexes, protein pairs, or individual proteins between the test and training sets, eliminating data leakage concerns and potential correlation structure within the high-throughput datasets among proteins of the same complex. The training split consists of 2177 known directly interacting pairs of proteins (Supplementary Data 1) and 1160 indirectly interacting pairs from the same complex, of which we have 1472 and 1001 entries in our feature matrix, respectively (Table 1). The limited number of indirectly interacting pairs (i.e., training negatives) prompted us to supplement the training set to include pairs from separate complexes, which resulted in a total of 100,707 indirectly and non-interacting (i.e., non-co-complexing) pairs of proteins (Supplementary Data 2) of which we have 47,246 entries in our feature matrix (Table 1). The test split consists of 638 known directly interacting pairs of proteins (positives) and 898 indirectly interacting co-complex pairs (negatives) (Supplementary Data 3 and 4), of which we have 572 and 785 entries in our feature matrix, respectively (see Table 1). To create a realistic evaluation scenario, our test set only includes complexes with ≥5 components to remove trivial cases of all components of a complex directly contacting and only includes negative labels within the same complex. These counts represent the entirety of the curated benchmark, which we provide as a resource. The final subset of protein pairs used for model training and evaluation are those that are represented in our proteomics-derived feature matrix (see “Methods”).

**Table 1:** Number of test and training benchmark interactions

	Complexes	Positive labels	Positive labels with feature matrix entries	Negative labels (intra-complex)	Negative labels (intra-complex) with feature matrix entries	Negative labels (intra- and inter-complex)	Negative labels (intra- and inter-complex) with feature matrix entries
Test	50	638	572	898	785	41,622	25,717
Train	595	2177	1472	1160	1001	100,707	47,246

We integrated the features described above, labeled with our training set using AutoGluon, a flexible machine learning model selection framework^35^. AutoGluon fits multiple models to training data, including random forests and neural networks, and evaluates each model’s accuracy. AutoGluon also utilizes ensemble methods, such as stacking and boosting to further increase accuracy and manage overfitting.

Using AutoGluon, we first trained 13 base models on our labeled proteomics data. AutoGluon then trains a WeightedEnsemble_L2 model using all 13 base models (Supplementary Data 7). Only the LightGBM base model resulted with a weight >0 after training the final WeightedEnsemble_L2 model, meaning that the final WeightedEnsembled_L2 model was equivalent to the LightGBM base model. We then applied this final classifier to 25,273,872 protein pairs, producing a confidence score for each interaction (Supplementary Data 8).

### DirectContacts2 network outperforms previous networks on the leave-out set of physically contacting protein pairs

**Fig. 2:**
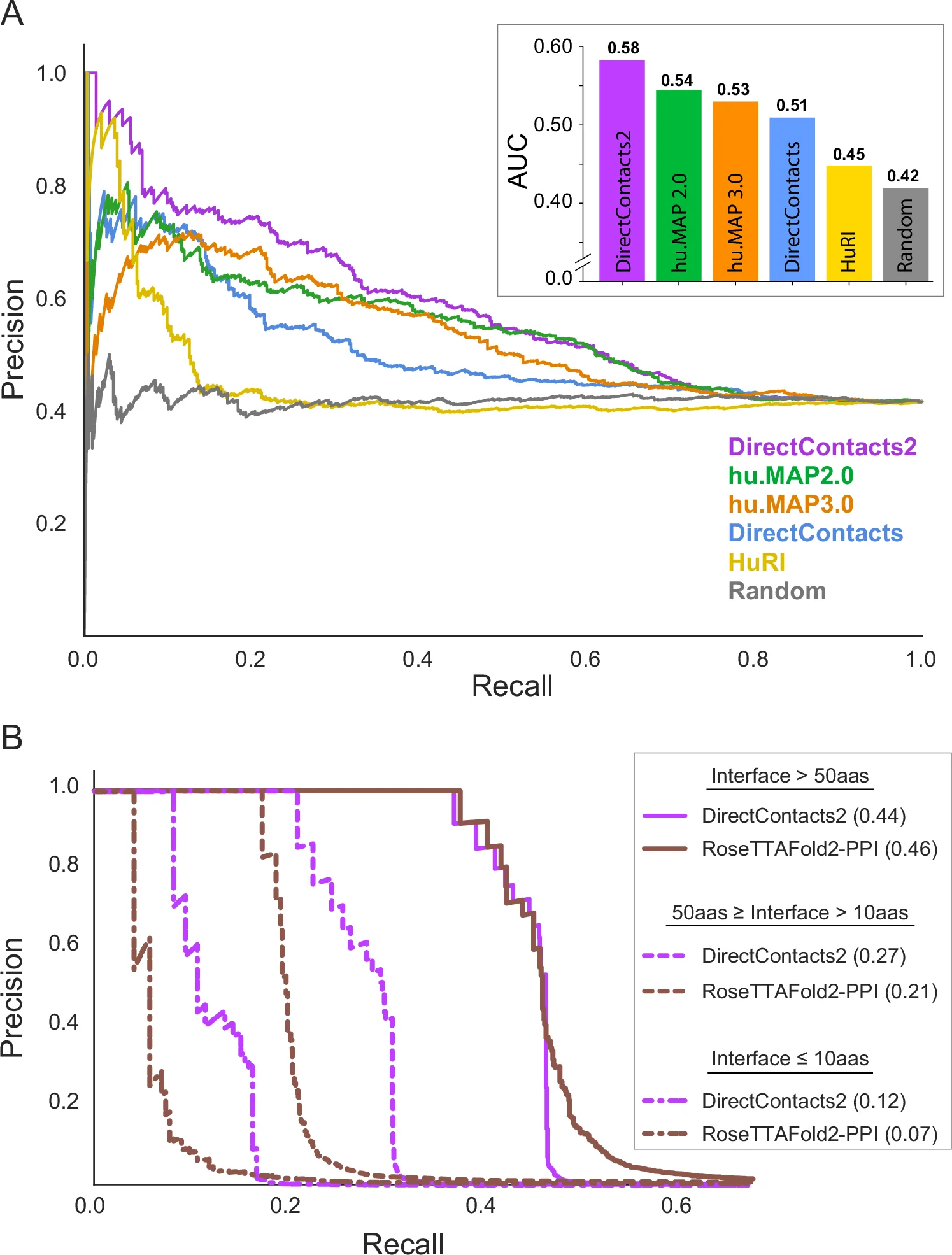
DirectContacts2 is highly accurate compared to alternative networks. **A** Precision recall analysis shows the DirectContacts2 model (purple) outperforms the HuRI network (yellow), hu.MAP2.0 (green), hu.MAP3.0 (orange), and the original DirectContacts model (blue). A random shuffling of pairs is shown in gray. Analysis is performed on the benchmark leave-out set of direct (positive) and indirect (negative) co-complex protein interactions. *Inset*: area under the precision–recall curve bar plot shows quantitatively that DirectContacts2 outperforms other models and networks. **B** Precision recall analysis comparing DirectContacts2 and RoseTTAFold2-PPI using the benchmark developed in Zhang et al.^15^. The benchmark is split into high affinity (PPI Interface > 55aas), medium affinity (50aas > PPI Interface > 10aas), and weak affinity (PPI Interface < = 10aas). Area under the precision–recall curve is shown in parentheses. Source data are provided as a Source Data file.

We next evaluate our DirectContacts2 predictions in comparison to other direct and co-complex networks using the same leave-out test set of complexes constructed from solved three-dimensional structures (described above). Performing a precision–recall analysis using this leave-out set, we observe that our DirectContacts2 predictions outperform the hu.MAP2.0 network, the hu.MAP3.0 network, the HuRI yeast2hybrid network, as well as our previous DirectContacts model (Fig. 2A). The Random curve represents the background probability of identifying a positive label with ~40% of direct pairs in the test set. Figure 2A (inset) shows the area under the precision–recall curve (AUC) for each network, and again demonstrates that DirectContacts2 outperforms other networks. Supplementary Fig. 2A shows a receiver operator curve (ROC) which is consistent with Fig. 2A. Additional metrics comparing networks can be found in Table 2.

**Table 2:** Performance metrics of networks on the test benchmark

Model (threshold)	Precision	Recall	F1	MCC	FDR	Top k-rank predictions
DirectContacts2 (≥0.9, high)	**0.771**	0.1	0.178	0.173	**0.229**	83
DirectContacts2 (≥0.7, low)	0.668	**0.306**	**0.419**	**0.248**	0.43	292
DirectContacts1 (high)	0.723	0.094	0.166	0.149	0.277	83
DirectContacts1 (low)	0.507	0.232	0.318	0.090	0.493	292
hu.MAP2 (high)	0.723	0.094	0.166	0.149	0.277	83
hu.MAP2 (low)	0.603	0.276	0.378	0.184	0.397	292
hu.MAP3 (high)	0.699	0.091	0.161	0.137	0.301	83
hu.MAP3 (low)	0.623	0.285	0.391	0.204	0.377	292
HuRI (high)	0.554	0.072	0.128	0.067	0.446	83
HuRI (low)	0.455	0.208	0.286	0.039	0.545	292
Random (high)	0.410	0.053	0.094	-0.003	0.590	83
Random (low)	0.401	0.183	0.252	-0.014	0.599	292

Highest ranking values are highlighted in **bold**.

Since our training set includes negative pairs from different complexes, we next asked if adding additional negative pairs from separate complexes into the test set alters the performance of our network. Supplementary Fig. 2B shows the precision–recall curve for DirectContacts2 and other major networks on the test set with expanded negative labels (Supplementary Data 5). We observe the DirectContacts2 network still outperforms all other tested networks similar to Fig. 2A. We next filtered our network by known co-complex associations in hu.MAP3.0^28^, CORUM^36^, and ComplexPortal^37^ (Supplementary Data 9), a strategy we previously showed improved the quality of a direct contact network^19^, and we anticipated would aid in relieving minor pathologies of our network, such as promiscuous proteins (see Discussion). We observe that filtering our network based on known complexes has near equivalent performance on this test set (Supplementary Fig. 2C).

The DirectContact2 score of this network shows a bimodal distribution (Supplementary Fig. 2D), with most pairs having low DirectContacts2 scores, while a substantial peak is seen with high DirectContacts2 scores. This observation is consistent with our expectation that most protein pairs do not interact directly, while a subset of pairs that interact directly are identified by our model. Based on this distribution, we selected a DirectContacts2 score threshold of ≥0.9, which resulted in 6967 confident pairs covering 7164 total proteins (36% of the human proteome).

Supplementary Fig. 2E shows the distribution of DirectContact2 scores for positively labeled (i.e., directly interacting) and negatively labeled (i.e., indirectly interacting) pairs from our leave-out test set. We see significant separation between the two distributions where positive labeled pairs are scored higher than negatively labeled pairs (Mann–Whitney two-sided test *p*-value: 4e-18), indicating our model’s ability to distinguish the two classes, consistent with our precision–recall analysis above. We therefore consider a lower confidence threshold of ≥0.7, resulting in 16,461 confident pairs covering 10,385 proteins (52% of the human proteome) based on this evaluation of the leave-out test set (Supplementary Fig. 2E, see below), which may be appropriate for certain applications requiring higher recall. Further, the relationship between precision and the DirectContacts2 confidence score is well-calibrated (Supplementary Fig. 2F) where, for example, a confidence score of 0.7 results in a precision ~0.7.

Additionally, we compare DirectContacts2 to the RoseTTAFold2-PPI method^15^, which uses sequence information to identify co-evolving residue pairs between proteins to identify protein interactions. To provide an independent analysis from our constructed benchmark, we utilized the RoseTTAFold2-PPI benchmark of direct interactions, which separates interactions into high affinity (PPI interface > 50aas), medium affinity (50aas > PPI interface > 10aas), and weak affinity (PPI interface < = 10aas). We see in Fig. 2B that DirectContacts2 and RoseTTAFold2-PPI have comparable performance on high-affinity interactions, while on medium-affinity and weak-affinity interactions, DirectContacts2 outperforms RoseTTAFold2-PPI. Since these methods have very different source information (i.e., DirectContacts2 is proteomics-based and RoseTTAFold2-PPI is sequence-based), we anticipate these methods to be complementary. In Supplementary Fig. 2G, we see an overlap of only ~3500 PPIs, suggesting future work may find value in integrating these two methods.

### Investigation of DirectContact2 predictions of a multisubunit complex: chaperonin CCT-prefoldin complex

**Fig. 3:**
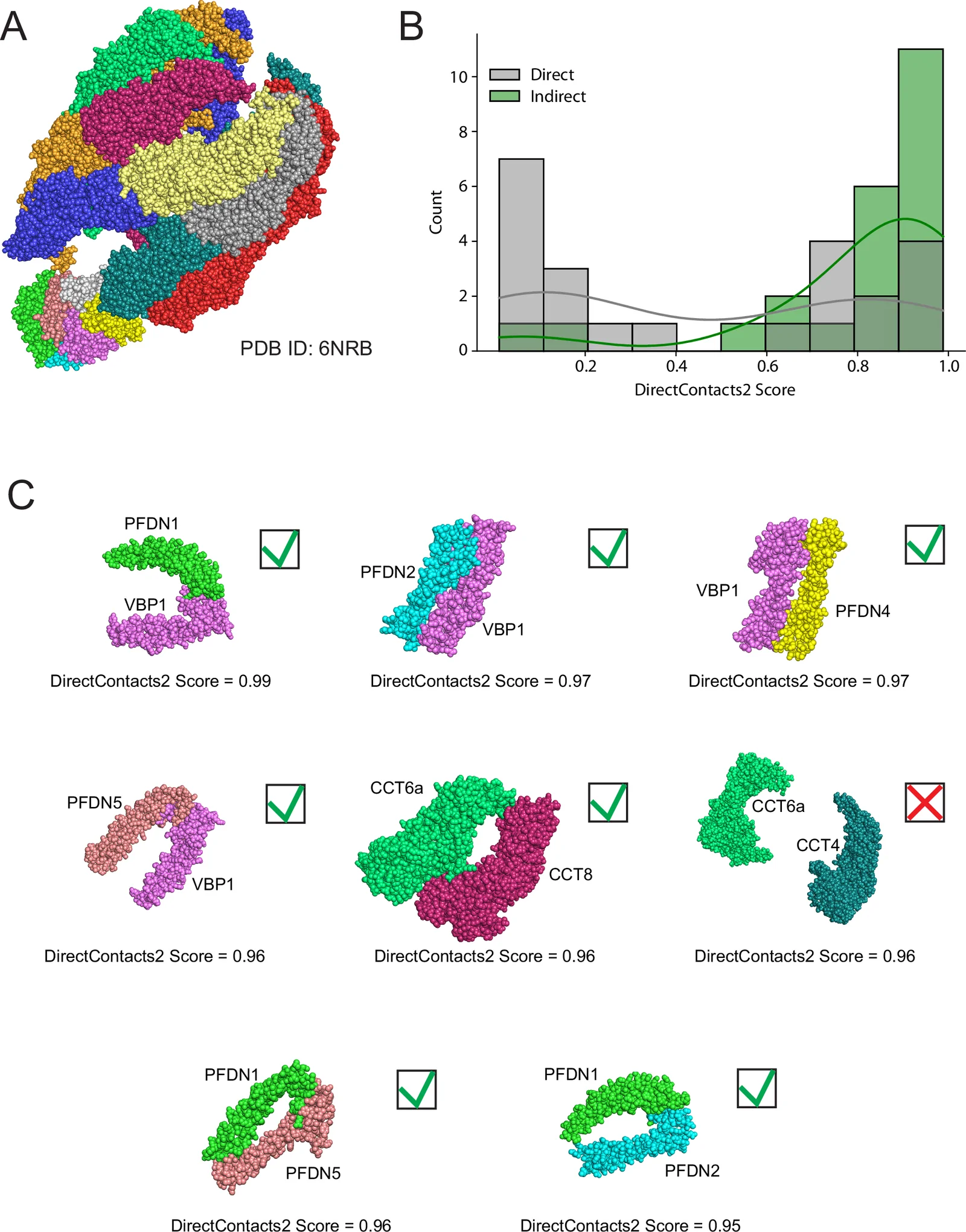
DirectContacts2 accurately identifies physical interactions of the chaperonin CCT-prefoldin complex. **A** Three-dimensional structure of the chaperonin CCT-prefoldin complex from the test leave-out set (PDBID: 6NRB). **B** Distributions of DirectContact2 scores for directly interacting pairs (green) and indirectly interacting pairs (gray) from chaperonin CCT-prefoldin complex. The directly interacting pairs have a right-shifted distribution with higher DirectContact2 scores, Mann–Whitney two-sided test *p* = 0.002. **C** Eight highest scoring protein pairs within the chaperonin CCT-prefoldin complex. Seven of the 8 high scoring pairs correctly predict direct interactions. Green checkmark = true positive, red X = false positive. Source data are provided as a Source Data file.

We next look at our model’s ability to discriminate between direct and indirect interactions on a specific example of a multisubunit complex from our leave-out set of test complexes. In Fig. 3A, we show the three-dimensional structure of the chaperonin CCT-prefoldin complex (PDBID: 6NRB^38^) with each unique protein chain colored differently. DirectContacts2 makes predictions for 46 pairwise interactions within the complex, 23 direct and 23 indirect. Figure 3B shows the DirectContacts2 confidence score distributions for these two sets of predictions, in which a clear distinction can be seen between the known positives and negatives, with direct interactions receiving higher scores by the model. The two distributions are significantly distinct using the Mann–Whitney two-sided test (*p* = 0.002). Notably, of the top eight highest scoring pairs of proteins from the chaperonin CCT-prefoldin complex, seven are true positives, and only one is a false positive (Fig. 3C). Alternatively, two false negatives are seen at the CCT-prefoldin interface (PFDN6–CCT4 and PFDN4–CCT4). This is expected as the majority of the signal in our ML framework is from biochemical isolation of subcomplexes (e.g., CF-MS, AP-MS) and pairs across stable subcomplexes are rarely seen in isolation of those subcomplexes (see Discussion). Interestingly, however, we do see limited evidence for direct interactions across stable subcomplexes. For example, when viewing elution profiles of CCT/prefoldin subunits in co-fractionation experiments, we observe CCT4 has the highest correlation with prefoldin subunits. This suggests there is a signal present for these interactions, albeit currently below the threshold of detection by our computational method (Supplementary Fig. 2F, G, pink box). In comparison, directly interacting subunits with stronger affinity, such as CCT4-CCT7 and CCT3-CCT6, do readily cluster together and are detected by our method (Supplementary Fig. 2H, I, yellow and orange box). Overall, the distributions seen in Fig. 3B align with our global analysis for the complete test set (Supplementary Fig. 2E) and further confirm that our DirectContacts2 network successfully discriminates between direct and indirect protein interactions within specific examples on which it was not trained.

### Enrichment of high-confidence multichain AlphaFold2 models in DirectContact2 predictions

Since the advent of AlphaFold2/AlphaFold-Multimer and RoseTTAFold^6,8,9^, several groups have applied these new methods to modeling protein interactions within the human proteome^12–16^. Due to the scale of all possible pairwise interactions across a proteome (*N*^2^, where *N* is the number of proteins in the proteome), applying computational structural modeling to all proteome pairs is currently infeasible, and protein pairs must therefore be prioritized.

A recent study by Elofsson and colleagues applied their FoldDock algorithm^13^ based on AlphaFold2 to generate structural models for 10,207 high-ranking hu.MAP 2.0 co-complex interactions in addition to 55,773 interactions from HuRI, derived from high-throughput yeast2hybrid experiments^13,17,29^. Using a score metric (pDockQ) that evaluates the quality of the modeled interaction, they observed an enrichment of pairs with confident pDockQ scores (≥0.23) in our previous hu.MAP2.0 network. We therefore theorized that our DirectContacts2 network should achieve greater enrichment for models with high pDockQ values due to its more stringent focus on direct physical interactions rather than just co-complexing interactions compared to our hu.MAP networks, which are a mix of direct and indirect interactions.

**Fig. 4:**
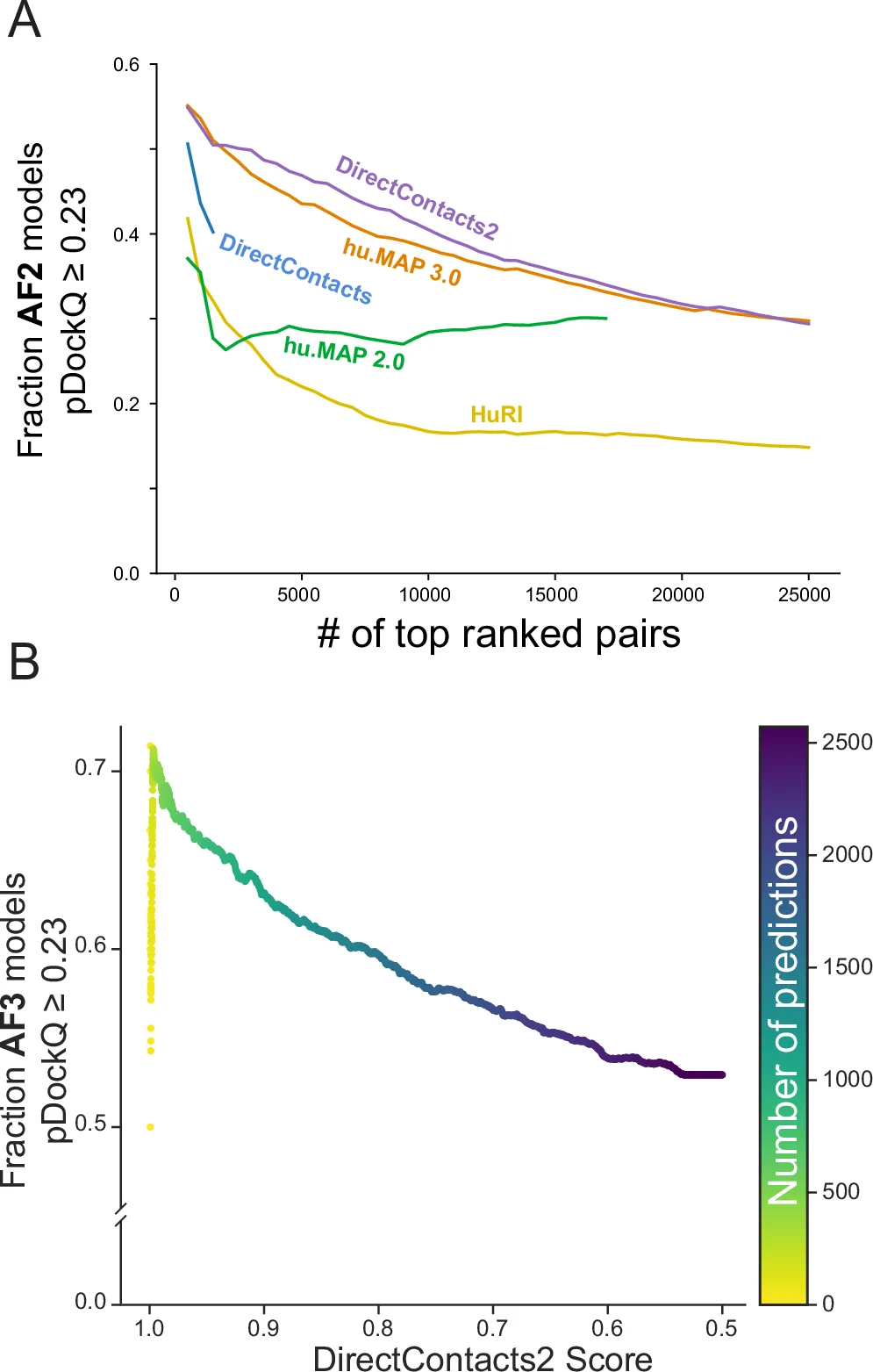
DirectContacts2 enriches for confident AlphaFold protein interaction structural models. **A** Comparison of enrichment for confident pair interfaces out of the set of top confident pairs for DirectContacts2 and other networks. For each set, the fraction of confident interfaces (pDockQ ≥ 0.23) out of their top predictions was calculated for increasing numbers of top predictions. Note: certain models made less predictions than others, and therefore their lines are truncated (e.g., DirectContacts1, blue line). **B** The fraction of AlphaFold3 protein interaction models with confident pDockQ pairs. Each dot represents the top N AlphaFold3 models ranked by their DirectContact2 confidence scores. Lower fraction values of confident models at the beginning of the curve are due to low sample size (see color bar). Source data are provided as a Source Data file.

To test if the DirectContacts2 model’s predictions are enriched for the quality of structural models over previous networks, we utilize an expanded set of 277,807 AlphaFold models^12,13^. To evaluate the networks on a fair basis, we calculate the fraction of each network’s most confident set of predictions with confident AlphaFold models (pDockQ ≥ 0.23) for increasing numbers of top-ranked pairs. In Fig. 4A, we observe 50% of the top 2500 (DirectContacts2 score > ~0.99) predictions contain confident AlphaFold models. This outperforms hu.MAP3.0^28^ (49%), DirectContacts1^19^ (40%), hu.MAP2.0^17^ (27%), and HuRI^29^ (28%). This trend remains consistent for all greater numbers of top predictions (Fig. 4A). To further assess the performance gain of filtering our network by known complexes, we compared our pre- and post-filtered DirectContacts2 network to the AlphaFold models and evaluated at a pDockQ ≥ 0.23. Supplementary Fig. 3A shows the complex-filtered network has a slight enrichment of confident AlphaFold models over the unfiltered network. This suggests that filtering by complex improves the overall accuracy of the network, likely by reducing false positives. Additionally, we perform the same rank-based analysis of pDockQ score enrichments between networks at a higher threshold for confidence (pDockQ ≥ 0.5)^39^, at which the relative enrichment of DirectContacts2 pairs for interface quality over other networks is consistent with Fig. 4A (Supplementary Fig. 3B). Using a benchmark of large heteromeric protein complexes, the Elofsson study highlighted that 38% of known directly interacting pairs resulted in having high-confidence pDockQ scores (≥0.5), suggesting an upper bound^13^. Comparing this with our result in Supplementary Fig. 3B suggests that our DirectContact2 model is near the upper bound of performance for high-confidence interactions. We next asked how complementary the networks are in the high-confidence AlphaFold predictions. Supplementary Fig. 3C shows an UpSet plot of the top 5000 predictions from DirectContacts2 and other networks. We see that most high-confidence predictions from each network are not found in the high-confidence predictions of other networks. This suggests that the networks themselves each provide unique interactions, and future work may consider a combination of networks.

A network of direct interactions should aim to encompass all interactions that bind directly, including those with high intrinsic disorder content that are difficult to model structurally. Looking at the relationship between disorder and structural confidence of high-confidence DirectContacts2 pairs (DirectContacts2 score ≥ 0.9), we see a slight anticorrelation (Pearson correlation coefficient = −0.215) between disorder content and pDockQ score (Supplementary Fig. 3D). We identify several protein pairs with high disorder content that are confidently predicted by DirectContacts2, but AlphaFold is unable to model confidently. For example, spliceosome subunits SNRPB (72% disorder) and SNRNP70 (66% disorder) have a DirectContacts2 score of 0.92 but a pDockQ score of 0.06. Additionally, SOSS complex subunits NABP2 (58% disorder) and INIP (60% disorder) have a DirectContacts2 score of 0.99 but a pDockQ score of 0.06. Overall, this suggests DirectContacts2 manages to identify protein interactions with high disorder content that are inaccessible by structural modeling.

### Generation of AlphaFold3 models from high-confidence DirectContacts2 predictions

While we observed a general enrichment for higher quality interfaces in existing AlphaFold structural datasets, we wanted to evaluate the practical utility of our DirectContacts2 score towards guiding new structural predictions. We produced AlphaFold3 models for 2573 pairs that our model predicted to have a DirectContacts2 score ≥ 0.5, and then assessed their pDockQ scores. We see in Fig. 4B a similar trend to our analysis above, in which high-confidence DirectContacts2 pairs (DirectContacts2 score ≥ 0.9) exhibit a greater enrichment for structural quality when modeled with AlphaFold3. A table of AlphaFold3 predictions and their scoring metrics can be found in Supplementary Data 10.

### Intermolecular cross-links are enriched in the DirectContact2 network

**Fig. 5:**
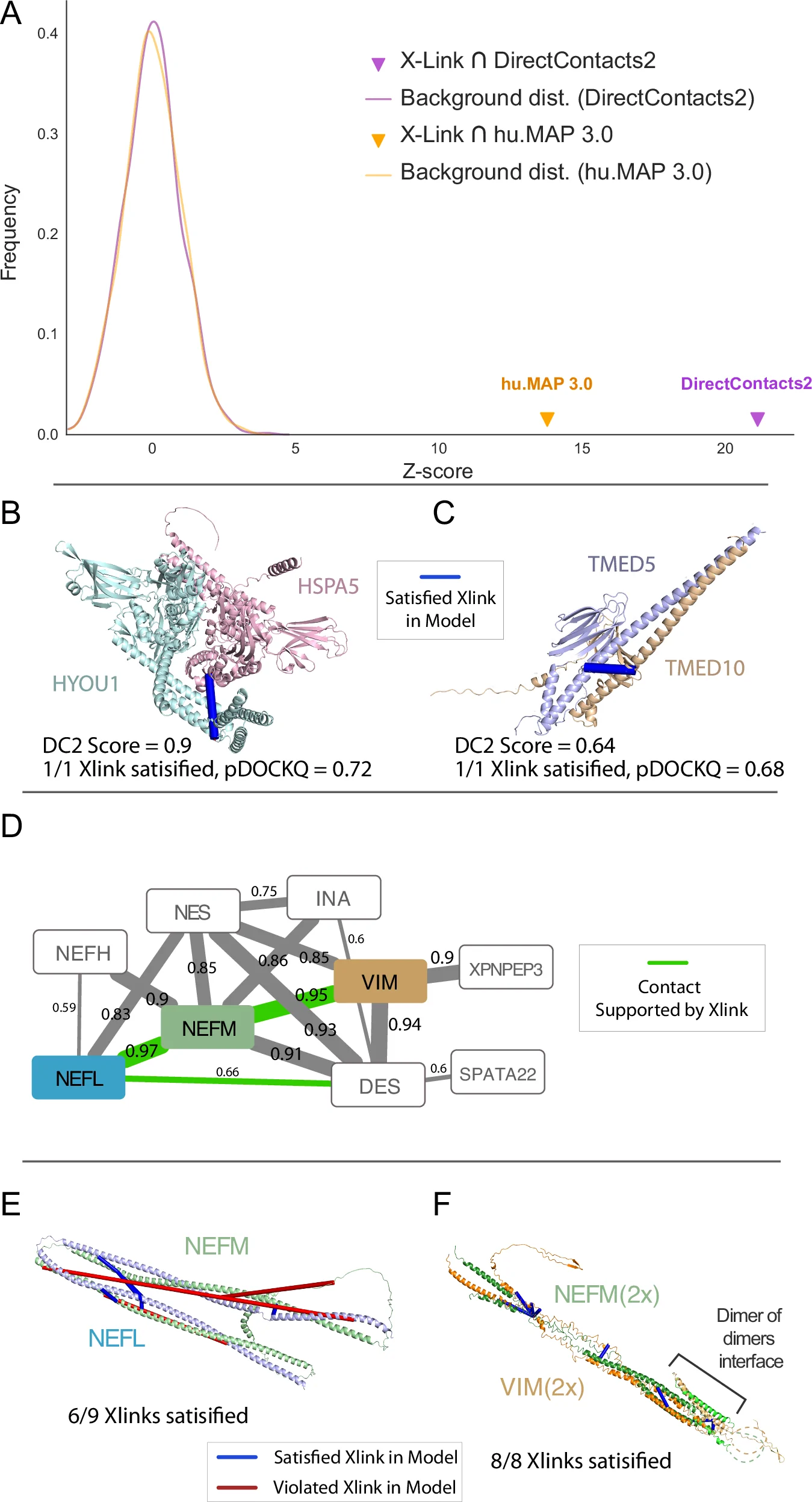
Crosslinking supports DirectContact2 predictions. **A** Overlap of crosslinking pairs from Wheat et al. with DirectContacts2. DirectContacts2 Z-score (purple triangle) is calculated by comparing the overlap of DirectContacts2 pairs with crosslinked pairs to a distribution of overlap of random pairs and crosslinked pairs (purple line) (see “Methods”). DirectContacts2 Z-score is substantially increased in comparison to hu.MAP3.0 pairs’ overlap with crosslinked pairs (orange triangle). **B**, **C** Examples of high-confidence DirectContacts2 predictions (HSPA5-HYOU1 and TMED5-TMED10) supported by both AlphaFold2 models and crosslinks. Both examples show crosslink is satisfied (blue line) by the structural model. **D** DirectContacts2 network of intermediate filament complex (HuMAP2_01819). Multiple direct edges are supported by experimental cross-links (green). Value on each edge represents the DirectContacts2 score. **E** AlphaFold3 model of the highest confident DirectContacts2 prediction (NEFL-NEFM) from the intermediate filament complex (**D**). The majority of experimental crosslinks are satisfied (6 satisfied = blue, 3 violated = red). **F** AlphaFold3 model of the second-highest confident DirectContacts2 prediction (NEFM-VIM) from intermediate filament complex (**D**). The dimer of dimers model shows that all 8 experimental crosslinks are satisfied. Source data are provided as a Source Data file.

Mass spectrometry cross-linking (XL-MS) experiments have the ability to identify directly interacting pairs of proteins from in vivo experiments. Since no XL-MS experiments were used in the training of DirectContacts2, cross-linking provides an additional independent means of benchmarking the DirectContacts2 network. Using the set of 5401 intermolecular cross-linked protein pairs identified in Wheat et al.^40^, we observe a substantial overlap between the top DirectContacts2 predictions and the crosslink-derived network when compared to random pairs of proteins (Z-score = 21.1) (Fig. 5A, see “Methods”). Since the DirectContacts2 network was constructed using the same feature matrix as hu.MAP3.0, we asked whether this enriched overlap is due to the direct physical nature of DirectContacts2 as opposed to simply co-complex interactions. To answer this, we perform the same overlap analysis between the hu.MAP3.0 network’s top pairs and the XL-MS dataset. In total, DirectContacts2 identified 127 XL-MS pairs out of its 1418 predictions and hu.MAP3.0 identified 123 XL-MS pairs out of its 2239 predictions (see “Methods”), corresponding to hit rates of ~9.0% and ~5.5%, respectively. This results in a greater than 1.6-fold increase in cross-link recovery rate for DirectContacts2 over hu.MAP3.0. Comparing the two networks on the basis of their resulting Z-scores, we see a 1.5-fold increase in overlap of our DirectContact2 predictions with protein crosslinks when compared to hu.MAP3.0 high-ranking pairs (Z-score 21.1 vs. 13.8) (Fig. 5A), demonstrating an enrichment for direct physical interactions above co-complex interactions.

We next focus on individual examples of interactions with cross-link support and show agreement between DirectContacts2 predictions, XL-MS cross-links, and AlphaFold2 models. Figure 5B shows the pair HSPA5 and HYOU1, which has a highly confident DirectContact2 score of 0.9 and a single experimental MS cross-link HSPA5 Lys370 and HYOU1 Lys883. Although there is no experimental structural coverage of these two proteins, an AlphaFold2 model of HSPA5 and HYOU1 from Burke et al.^13^ shows the MS cross-link between these residues is satisfied (pDockQ = 0.72). Similarly, the pair of proteins TMED5 and TMED10 was predicted to directly interact near our confident cutoff (DirectContacts2 score = 0.64) and has an experimental MS cross-link (TMED5 Lys169 and TMED10 Lys170) that is satisfied by an AlphaFold2 model (pDockQ = 0.68, Fig. 5C). This example highlights that a combination of orthogonal data, such as XL-MS, can provide supporting evidence for direct interactions. As a whole, these examples demonstrate concordance between DirectContacts2 and cross-linking data and further point to the ability to build high-quality structural models for protein complexes using DirectContacts2 as a guide.

We next turn to a larger complex with high-confidence DirectContacts2 predictions, which does not have an experimental structure. The intermediate filament complex (HuMAP2_01819) consists of several filament subtypes, but knowledge of their true direct interactions is limited (Fig. 5D). Intermediate filaments are involved in cytoskeleton construction, and their disruption leads to several human diseases^41^. Our DirectContacts2 model identifies confident direct interactions among several pairs of subunits in the complex. In particular, we observe the NEFL-NEFM and NEFM-VIM pairs to have the highest confidence scores within the complex, 0.97 and 0.95, respectively. We identify XL-MS crosslinks^40^ supporting these interactions, providing additional evidence for these direct interactions (Fig. 5D). We next modeled the two subcomplexes, NEFL-NEFM and NEFM-VIM, using AlphaFold-multimer to gain structural intuition of the interactions. Both resulting models produced two elongated coiled coil structures linked by a short loop region (Fig. 5E, F). Supporting these structures, recent work by Jänes et al. have generated AlphaFold models for both pairs, NEFL-NEFM and NEFM-VIM, both of which produced confident pDockQ values of 0.72^12^. We next map XL-MS cross-links from the Wheat et al. set onto our NEFL-NEFM AlphaFold-multimer model and observe that the model satisfied 6 out of 9 experimental cross-links. Intriguingly, the residues of 1 crosslink in the NEFL-NEFM model, NEFL:Lys91-NEFM:Lys271, are located at opposite ends of the structure with a distance of ~212 Å. This suggests the crosslink may be satisfied by a higher-order oligomerization of the NEFL-NEFM subcomplex.

We next evaluated the NEFM-VIM subcomplex on a similar basis and constructed an initial NEFM-VIM model that satisfied 6 out of 8 crosslinks, with the 2 violated crosslinks on opposite ends of the structure (NEFM:Lys259-VIM:Lys120: ~211 Å, NEFM:Lys,271-VIM:Lys120: ~198 Å). McCafferty et al. recently showed large distances between cross-linked residues agree with oligomerization models of proteins^42^, and intermediate filaments are known to form higher-order structures^43^. We therefore theorized that these unsatisfied cross-links may point to sites of oligomerization. Using a structural model built from the expected overlapping segments, we constructed a higher-order model of NEFM and VIM. When modeled as a higher-order oligomer, the remaining unsatisfied cross-links between NEFM and VIM were satisfied (Fig. 5F). Taken together, we show using DirectContacts2 predictions with complementary structural data, such as XL-MS, aids in the modeling of protein complex structures.

### DirectContacts2 guides the structural model of a human ciliopathy complex

**Fig. 6:**
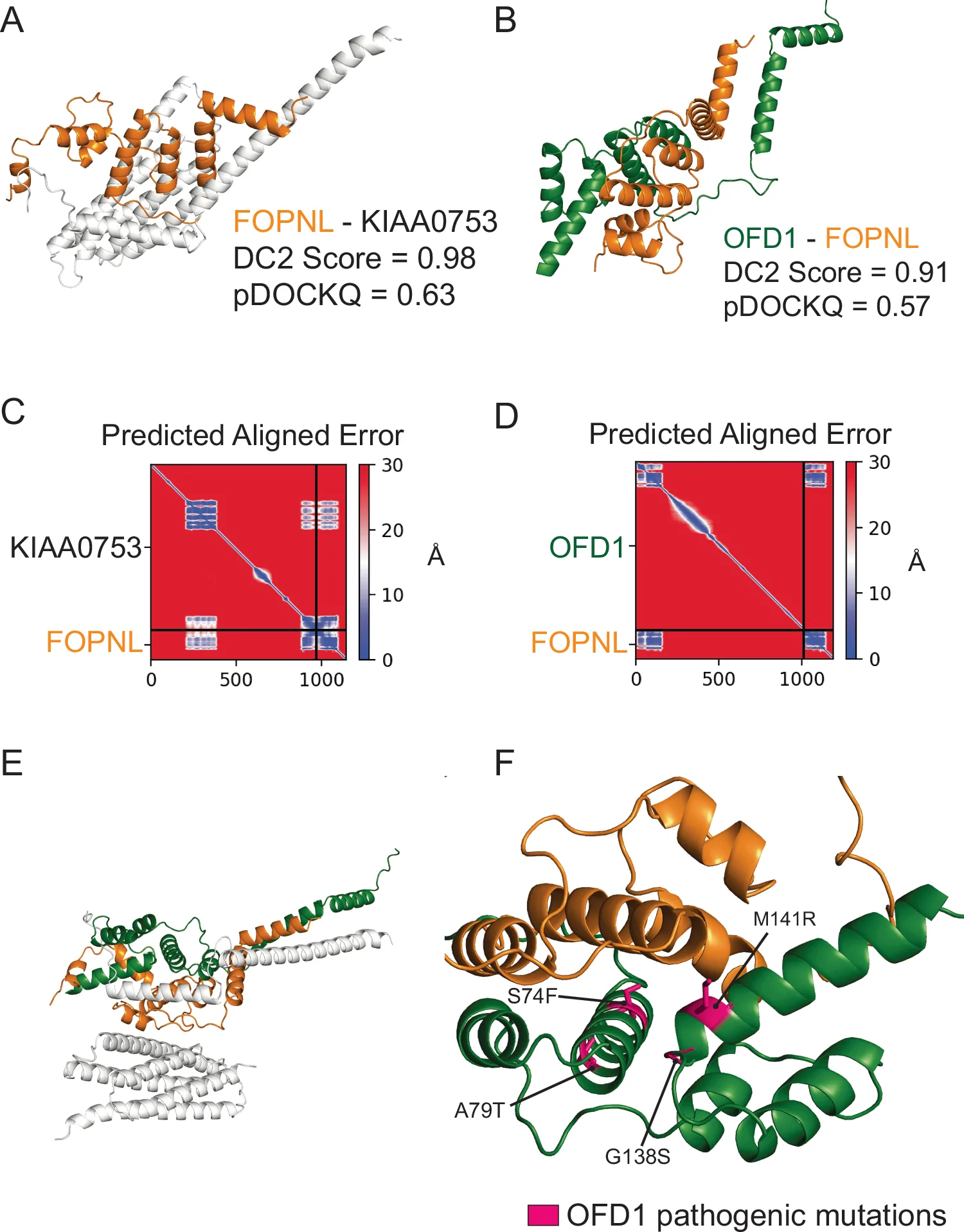
Direct contact network of a ciliary protein complex informs structural modeling. **A** AlphaFold-Multimer structure prediction of FOPNL-KIAA0753, highlighting the area around the interface. **B** AlphaFold-Multimer structure prediction of OFD1-FOPNL, highlighting the area around the interface. **C** Predicted aligned error graph of the FOPNL-KIAA0753 AlphaFold-Multimer prediction. **D**. Predicted aligned error graph of the OFD1-FOPNL AlphaFold-Multimer prediction. **E** Predicted interface of the OFD1-FOPNL-KIAA0753 complex by overlapping FOPNL chains. **F** OFD1 disease mutations (pink) cluster at the OFD1-FOPNL interface.

OFD1-PIBF1-FOPNL (CEP20, FOR20)-KIAA0753 form a complex whose members localize to the distal ends of basal bodies and are important for proper ciliogenesis^44,45^. Mutations in these genes are causative of ciliopathy disease, such as orofaciodigital syndrome 1, characterized by malformations in the face, oral cavity, and digits in females and neonatal lethality in males. In particular, four missense mutations (S74F^46^, A79T^47^, G138S^48^, and M141R^49^) in the OFD1 protein give rise to serious clinical phenotypes in human patients. However, there is currently no experimental structural data to interpret these mutations in the broader structural context of the proteins’ molecular interactions. Our hu.MAP2.0 network indicates that these four proteins are components of a larger complex (HuMAP2_00328), the Orofacial Digital Syndrome (OFDS) complex. Of the pairwise interactions in this complex, FOPNL-KIAA0753 and OFD1-FOPNL exhibit the highest DirectContact2 scores of 0.98 and 0.91, respectively. To place these mutations into their three-dimensional context, we therefore used our DirectContacts2 predictions to direct AlphaFold-multimer modeling of the three-dimensional structure of FOPNL-KIAA0753 and OFD1-FOPNL (Fig. 6A, B). The interfaces of the models resulted in high-confidence pDockQ scores of 0.63 and 0.57, respectively, indicating accurate modeling of the dimers. The predicted aligned error plots produced by AlphaFold-multimer (Fig. 6C, D) further suggest high global confidence of the structure. The FOPNL-KIAA0753 model was consistent with previous work that identified the C-terminus of KIAA0753 as being necessary and sufficient for the binding of FOPNL^44^.

Additionally, we observed another region of KIAA0753 (aa 206–380) that formed a substantial interface with FOPNL, with an average per-residue pLDDT value >80. To construct a higher order structure of the complex, we aligned the two structure models on their common subunit (FOPNL) and observed distinct interfaces between the three subunits (Fig. 6E). The resulting model placed four sites of OFD1 mutation (i.e., S74F^46^, A79T^47^, G138S^48^, and M141R^49^) at the interface of FOPNL (Fig. 6F), strongly suggesting that the disruption of this interface is responsible for the downstream functional defect giving rise to a clinical phenotype.

### Signal from individual features

Machine learning algorithms excel at integrating multiple lines of evidence from orthogonal sources towards building powerful classifiers. We next asked which features provide the most signal in discriminating between direct and indirect protein interactions. In Supplementary Fig. 4A, we show the top 20 features ranked based on the calculated area under the precision–recall curve (AUPRC) evaluated on the benchmark used to train our classifier. We have shown in previous work that CF-MS features could be used as powerful predictors of physical interactors^19^. Here, we see again that CF-MS data provides strong predictive signals of interaction, with 16 of the top 20 features in the DirectContacts2 model being CF-MS features. We also see AP-MS features exhibiting strong predictive capability, including the Bioplex3 features “uPeps,” which represent the quantification of the number of unique peptides for a given prey protein. These features originate from two cell types in the Bioplex3 dataset, HCT116 cells and HEK293T, and perform very well as discriminators of direct and indirect interactors. Additionally, we observe WMM features among the top features, suggesting that our WMM can discriminate between direct and indirect protein interactions as well.

Somewhat unexpectedly, WMM features calculated from proximity labeling experiments^26,27^ had very high performance under the AUPRC measure (Supplementary Fig. 4A). Due to the diffusible nature of labeling in proximity labeling techniques, features from individual proximity labeling experiments are not expected to be valuable discriminators between direct and indirect interactions. In fact, we see a limited number of pairs in our training set (9 total pairs) that have evidence from proximity labeling experiments (non-WMM features) where 1 subunit is a bait and the other is the prey. Furthermore, several bait-prey proximity labeling features representing data from an individual experiment are automatically removed from consideration by the AutoGluon framework during training due to their limited signal. While an individual proximity labeling experiment doesn’t appear to have a signal for this application, the WMM, which statistically analyzes many experiments collectively, shows substantial statistical power for discriminating between direct and indirect protein interactions (neg_ln_pval_youn_hygeo_gt4 and neg_ln_pval_cilium_hygeo features, Supplementary Fig. 4A, see also Supplementary Data 11).

To investigate this further, we return to the example of the CCT-prefoldin complex interactions. The prefoldin complex is a chaperone that physically engages with the TRiC/CCT complex to prevent misfolding and help maintain proteostasis. As described above, a recent cryo-EM structure of prefoldin in complex with the TRiC/CCT provides us with a specific test case of our WMM feature^38^. Supplementary Fig. 5A shows a heatmap of 20 proximity labeling experiments where at least 1 prefoldin subunit is identified above an abundance threshold (see “Methods,” ≥4 average PSMs). We observe two distinct regions that suggest individual prefoldin subunits are isolated together without other subunits. Specifically, we see PFDN5 and PFDN3 (highlighted box in yellow) as well as PFDN3 and PFDN2 (highlighted box in purple) are seen in several shared experiments, pointing to their likelihood to directly contact each other. Notably, we do not see PFDN1 and PFDN6 together without the other subunits of the complex, which is consistent with their spatial separation and lack of a direct interface as shown in the cryo-EM structure of the prefoldin complex (PDBID: 6NRB) (Supplementary Fig. 5B).

We next evaluate our calculation of the hypergeometric test on these data, as we expect it to more finely discriminate direct from indirect interactions. The hypergeometric test is a rigorous statistical test, which asks how often a pair of proteins are seen together in the same set of experiments, and are they seen more often than random chance. As described above, this is the central concept of the WMM calculation. To discriminate between direct and indirect interactions, we expect the values calculated by the hypergeometric test to exhibit a bimodal distribution where indirect interactions correspond to lower scores than direct interactions. Supplementary Fig. 5C shows the distribution of the -lg(Pval) calculated from the hypergeometric test for all observed pairs of prefoldin subunits and exhibits the expected bimodal topology (direct = higher scores, right density and indirect = lower scores, left density), with the PFDN5/PFDN3 and PFDN3/PFDN2 pairs having higher overall scores.

Comparing this distribution to the cryo-EM structure of prefoldin, we see that PFDN3 and PFDN5 form substantial direct interfaces (Supplementary Fig. 5B). Similarly, we see that PFDN3 and PFDN2 also make direct physical contact with each other in the structure, further validating our observations. Lastly, PFDN1 and PFDN6 do not interact, as shown by their locations on opposite sides of the complex, which is also quantitatively reflected in their placement within the left density of the bimodal -lg(Pval) distribution (Supplementary Fig. 5C). The heatmap (Supplementary Fig. 5A) also reflects this as PFDN1 and PFDN6 are seen in limited number of experiments and importantly, never without other prefoldin subunits. These results show, on an individual example, how the WMM computed on proximity labeling experiments can discriminate between direct and indirect protein interactions, and when taken together with our results from Supplementary Fig. 4A, demonstrate the power of WMM in conjunction with proximity labeling towards predicting direct interactions on the proteome scale.

### Relationship of DirectContacts2 features and model confidence

To better understand the relationship between the experimental features used to train the DirectContacts2 model and the confidence of the model itself, we compared the fraction of pairs that contained the top 20 features for various confidence thresholds (Supplementary Fig. 4B). As expected, we see the top features are more likely in high-confidence pairs than low-confidence pairs. Additionally, we see AP-MS features (e.g., uPeps_bp3_293T and uPeps_bp3_HCT116) are found in many confident predictions, while other predictive features are found in fewer total pairs. This is largely due to AP-MS having high coverage. It is also important to remember that multiple lines of limited evidence often give rise to confident predictions. It is expected that many of these top features with low total pairs are combined by our model with other features to increase confidence in the predictions.

Further, our model integrates several orthogonal classes of experimental data, specifically AP-MS, CF-MS, proximity labeling (WMM), and RNA-pulldowns (WMM). We asked how our model’s confidence changes when a prediction has many orthogonal pieces of evidence. In Supplementary Fig. 4C, we see the model is most confident for predictions with all feature classes. Interestingly, many predictions with only one source of evidence have low confidence (i.e., the highest peak in the low confidence region), but a subset are highly confident as well (i.e., additional peaks in the high confidence region). This is largely due to AP-MS features being very accurate, and additional evidence is unneeded to make a confident prediction.

### DirectContacts2 identifies interactions among uncharacterized proteins

We next highlight our network’s ability to identify interactions among uncharacterized proteins. H2BC17 is a poorly understood histone H2B variant with no homolog in mouse^50^. We identify a direct interaction between H2BC17 and H2AX, a histone variant primarily involved in DNA damage response^51^ (DirectContacts2 score = 0.95). AlphaFold3 produces a high-confidence model of this interaction (pDockQ = 0.72, Supplementary Fig. 6A, B). Further, the structural model satisfies a cross-link between these histone proteins, providing additional evidence of this interaction. We additionally identify interactions for other uncharacterized proteins, including C2ORF74 with PPP1CA (DirectContacts2 score = 0.99) and C15ORF61 with PCDHAC2 (DirectContacts2 score =  0.89). AlphaFold3 produces confident models for both interactions with pDockQ scores of 0.65 and 0.33, respectively (Supplementary Fig. 6C–F). These examples demonstrate the ability of DirectContacts2 to identify high-confidence direct interactions for uncharacterized proteins and place them into structural context.

### Analysis of the evolutionary age difference between directly interacting proteins

Protein complexes have been proposed to evolve by several mechanisms, including gene duplication^52^ and genomic gain or loss of individual subunits^53^. An additional model proposed is the assembly from preexisting modules, such as proposed for the NADH:ubiquinone oxidoreductase (complex I) complex, where the dehydrogenase module, the hydrogenase module, and the membrane-bound transporter module functioned separately but then assembled later in evolution to form complex I^54^. Certain modules, which later assemble into larger complexes during evolution, would have distinct evolutionary signals. To test the prevalence of this model, we reasoned that directly interacting protein pairs with similar evolutionary ages to each other but dissimilar ages to other members of their complex would represent candidates of this stepwise manner of evolution.

**Fig. 7:**
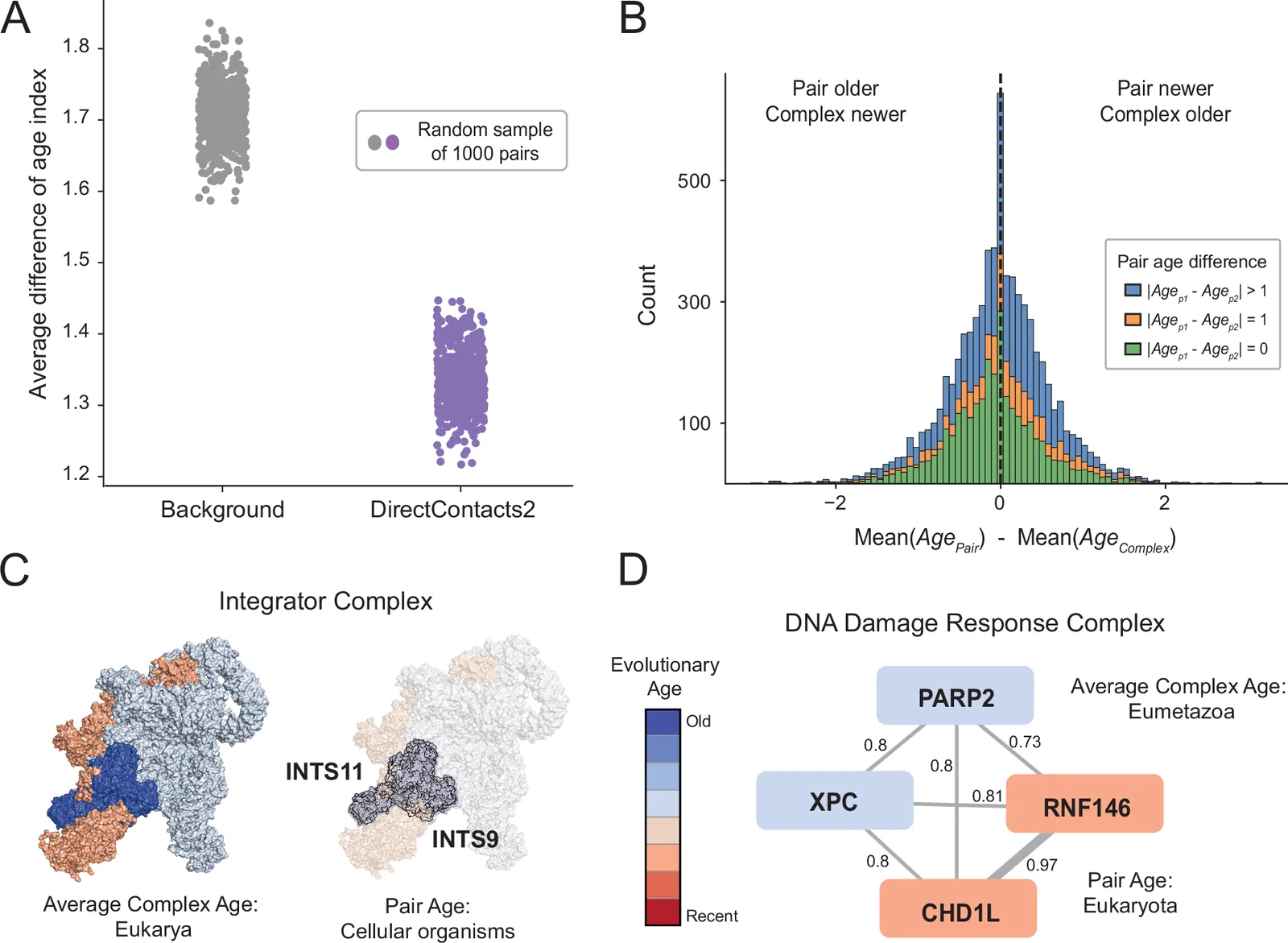
Evolutionary ages of direct interactions. **A** Scatter plot of the average pairwise age difference between high-confidence DirectContacts2 predicted pairs and random pairs. Each dot represents the average of a random sample of 1000 pairs (*n* = 500). **B** Stacked histogram of the difference between the mean evolutionary age of a given high-confidence DirectContacts2 interaction and the mean age of its associated complex. Color represents the difference in ages within the pair. **C** (Left) Structure of the integrator complex (PDB ID: 8RC4^73^) with each subunit colored according to its evolutionary age index, with cooler colors corresponding to older evolutionary ages and warmer corresponding to more recent ages. (Right) Same structure as left, with an outline overlay used to highlight the direct interaction identified by DirectContacts2. **D** Age network of a DNA damage response-related complex (huMAP3_08413.1) with high-confidence direct interactions between RNF146 and CHD1L. Nodes are colored by age as done in (**C**). Values on network edges correspond to the DirectContacts2 score of the interaction. Source data are provided as a Source Data file.

We first annotated DirectContacts2 pairs with evolutionary across eight numbered age bins: 0:* Cellular organisms,* 1: *Eukarya+Archaea,* 2: *Eukarya+Bacteria,* 3: *Eukaryota,* 4: *Opisthokonta,* 5:* Eumetazoa,* 6: *Vertebrata,* 7: *Mammalia*^55^. Using these annotations, we analyzed high-confidence direct pairs (DirectContacts2 score ≥ 0.9) to determine the degree of similarity in their protein ages against a random background of shuffled pairs. Comparing randomly sampled distributions of age differences between pairs shows direct pairs exhibiting a markedly lower mean difference in consensus age compared to the background (Fig. 7A). Using a one-sided permutation test, DirectContacts2 pairs showed significantly more similar ages relative to the random background (*P* < 0.0001, see “Methods”). We additionally plot the distribution of age differences for DirectContacts2 pairs and random pairs and see an increase in density of similarly aged proteins that directly interact (Supplementary Fig. 7). Together, these results demonstrate that proteins predicted to directly interact share similar evolutionary ages more than expected by chance.

We next test the prevalence of complexes forming via the stepwise addition of functional modules. Here, we assume directly interacting proteins that function together will share the same age. We, therefore, compared the average age index of DirectContacts2 pairs with that of their protein complexes (Fig. 7B). Positive values correspond to direct pairs that evolved more recently than their complexes, and negative values to direct pairs that are older than their complexes. The resulting distribution is largely normal and zero-centered, suggesting complex subunits evolve together and the stepwise combination of functional modules through evolution is likely rare.

Notable exceptions, however, exist in the extremes of the distribution where a pair’s age differs starkly from that of its complex. The integrator complex, a large 14-subunit transcription regulatory complex, contains directly interacting subunits, INTS9 and INTS11 (DirectContacts2 score = 0.98). The integrator complex is understood to be conserved across metazoa^56^, with many subunits having eukaryotic origin (age index = 3). However, its catalytic core, comprising INTS9 and INTS11, has an earlier evolutionary origin, with homology for the pair found in early cellular organisms (age index = 0) (Fig. 7C). INTS9 and INTS11 are therefore ancient relative to other subunits of the integrator complex (Age_pair_ − Age_cpx_ = −2.7). These results are consistent with recent work, which identifies INTS11 as homologous to the archaeal transcription termination factor FttA^57^.

We additionally identify CHD1L and RNF146 as directly interacting (DirectContacts2 score = 0.97), which both have an evolutionary age of eumetazoa (age index = 5) (Fig. 7D). CHD1L and RNF146 are part of a DNA damage response-associated complex (huMAP3_08413.1) whose other subunits are older and conserved through eukaryotes (age index = 3). This results in an Age_pair_ −  Age_cpx_ of 1.7. While difficult to determine if CHD1L and RNF146 were a functional module prior to their interaction with the remaining DNA damage response subunits, this suggests CHD1L and RNF146 may have formed a directly interacting pair before joining the rest of the complex later in evolutionary time.

## Discussion

Determining a comprehensive wiring diagram of the cell is an important challenge in biological research. A network of directly interacting biomolecules provides molecular mechanisms for understanding disease and molecular function. In addition, it provides a global view of how the cell is constructed, leading to functional insights of the system as a whole. Here, we construct DirectContacts2, a network of directly interacting proteins, by integrating a broad array of high-throughput proteomics experiments. Our integration was based on a machine learning framework trained on directly contacting proteins identified from experimentally solved protein structures (Fig. 1). We showed our network outperformed the state-of-the-art in identifying direct interactions (Fig. 2) as well as enriching for high-confidence AlphaFold2 models (Fig. 4). Furthermore, our network shows good concordance with crosslinking data, which is an orthogonal experimental method for identifying direct protein interactions (Fig. 5). We then demonstrate the utility of our network by building and analyzing AlphaFold models of protein interactions without known experimental structures including ~2500 AlphaFold3 models. Specifically, we analyze interactions with high DirectContact2 scores, including the HYOU1-HSPA5, TMED5-TMED10, NEFL-NEFM, NEFM-VIM interactions, and demonstrate support for their direct interactions using high-confidence AlphaFold models and crosslinking data (Fig. 5). We identify developmental disease proteins having high DirectContact2 scores with their interactors. When modeled, these interactions result in high-confidence AlphaFold2 models that provide mechanistic structural insight into several patient mutations’ roles in known clinical phenotypes (Fig. 6). Finally, we utilize our DirectContacts2 network to test modes of protein complex evolution and identify rare candidates of functional modules assembling into larger complexes throughout evolution (Fig. 7).

The development of highly accurate machine learning models relies substantially on well-curated training and test sets. Here, we carefully constructed a benchmark of direct interactions derived from the Protein Databank (PDB). For this problem, we developed a clustering algorithm for benchmark creation that controls data leakage without unnecessarily discarding training examples. Further, we considered the selection of negative examples to be as important as positive examples and therefore curated negative examples as only those with sufficient structural evidence to be certain the two proteins do not interact. Since the mass spectrometry experiments used for our DirectContacts2 model are the same as used for the hu.MAP3.0 model, it is interesting to note that hu.MAP3.0 does not outperform hu.MAP2.0 on the constructed leave-out test set (Fig. 2A) despite having an additional 10,000 proteomics experiments. The gain in performance of DirectContacts2 over hu.MAP3.0 is largely, therefore, due to the curation of the benchmark. We anticipate the benchmark used here for the DirectContacts2 model will be the de facto benchmark for this specific problem in the field moving forward.

While our DirectContacts2 network is more accurate than other state-of-the-art networks, our predictions still have an appreciable amount of error, both false positives and false negatives. Errors can arise through several mechanisms. To discriminate between direct and indirect interactions, our method attempts to extract signals from experimental proteomics data where subcomplexes are biochemically separated from their larger assemblies (see Supplementary Fig. 2H, I). As discussed previously^19^, subcomplexes of highly stable complexes (e.g., 20S proteasome core) are rarely seen apart from the entire complex in high-throughput AP-MS and CF-MS data, making it difficult to discern direct interactions. Alternatively, low-stability and transient complexes (e.g., kinase–kinase substrate interactions) are difficult to capture with the experiments utilized here. Discriminating direct interactions within highly stable complexes and capturing transient interactions, therefore, continues to be a challenge in the field. Identifying separation techniques that disrupt high-stability complexes or stabilize low-stability complexes may help capture these elusive interactions.

Alternatively, DirectContacts2 could be combined with orthogonal approaches such as machine learning methods, which use sequence co-evolution. As we show in Fig. 2B, on certain classes of proteins, DirectContacts2 outperforms RoseTTAFold2-PPI, a sequence-based machine learning method. Additionally, we see in Supplementary Fig. 2G, DirectContacts2 and RoseTTAFold2-PPI make confident predictions for an orthogonal set of protein pairs. This suggests the two networks are highly complementary, likely due to DirectContacts2 being based on high-throughput proteomics experiments while RoseTTAFold2-PPI is sequence-based. Moreover, Zhang et al. specifies certain features that limit RoseTTAFold2-PPI predictive ability, including MSA depth, selective pressure at the interface, conservation at the interface, number of interface residues, and interface residue variability^15^. None of these features directly apply to the DirectContacts2 method, again suggesting they are highly complementary.

The DirectContacts2 network is also biased towards high-abundance complexes. The power of integrating a large compendium of experiments comes from having multiple lines of evidence to inform prediction. Low-abundance proteins may rarely be seen in high-throughput proteomics experiments, which makes predictions difficult for these biomolecules. However, DirectContacts2 increases the total coverage of the human proteome over our previous DirectContacts network by ~7 times (DirectContacts2 proteins = 10,407 vs. DC1 proteins = 1504). Crosslinking datasets provide residue-level resolution of protein interfaces but also suffer from bias towards high-abundance proteins, among other challenges^58^. The Wheat et al. crosslinking dataset^40^, for example, identified crosslinks for 2291 proteins, ~4.5 times smaller than the DirectContacts2 network.

As described above, we have taken several approaches to globally address the error in our network. First, we filtered predicted pairs by membership in a previously identified protein complex. Second, we threshold predicted pairs in the network by the machine-learned confidence score. Interestingly, prior to filtering by protein complex, we noted a trend of several proteins making many interactions. Specifically, we observe 19 promiscuous proteins with >100 interactions (DirectContacts2 score ≥ 0.7). These proteins include heat shock proteins (e.g., HSPA5, HSPA8, HSPA9, and HSPA2), HLA proteins (e.g., HLA-C, HLA-B, and HLA-A), and tubulins (e.g., TUBB, TUBA1A, and TUBB4A). While we cannot rule out experimental artifacts playing a role in producing these interactions, the functions of these proteins suggest a high degree of connectivity. We provide our raw, unfiltered DirectContacts2 predictions in Supplementary Data 8 and suggest a threshold of ≥0.7 for confident interactions and a threshold of ≥0.9 for high-confidence interactions.

Further, a major challenge in the field remains to address the computational intractability of screening all by all pairs of proteins in a given proteome by structural modeling techniques (e.g., AlphaFold). Our network improves on the list of strategies to prioritize protein pairs for screening of protein pairs, eliminating large swaths of pairwise space unlikely to interact. Interestingly, orthogonal efforts to address this problem by pooling random pairs of proteins in AlphaFold runs was recently shown to increase both speed and accuracy of interaction screening^59^. The prioritization approaches and pooling approaches are, in theory, complementary and therefore can be combined to more efficiently screen entire interactomes.

Overall, the DirectContacts2 resource will be valuable for the structural, computational, and proteomics research communities. We anticipate the resource will have many applications, including prioritizing high-throughput computational structural modeling and identifying therapeutic targets that were once considered undruggable.

## Methods

### Compilation of mass spectrometry datasets

To generate our DirectContacts2 network, we utilized a feature matrix derived from published mass spectrometry experiments where each feature quantifies the statistical evidence for a pair of proteins to interact (Table 3). The feature matrix consisted of the same experiments used in Fischer et al. for the construction of hu.MAP3.0 co-complex interaction network^28^. Features representing CF-MS data from Wan et al.^18^ consist of 1-D vector comparison measures, including Poisson noise, Pearson correlation coefficient, weighted cross-correlation, co-apex score, and MS1 ion intensity distance metric. CF-MS features were filtered to ensure the pair of proteins contained at least one 1-D vector comparison score >0.5 in at least one human CF-MS experiment. Features from Bioplex1^22^ and Bioplex2^21^ included NWD score, *Z* score, plate *Z* score, entropy, unique peptide bins, ratio, total PSMs, ratio total PSMs, and unique:total peptide ratio. Bioplex3^20^ features included the same features as Bioplex1 and 2 for both HEK293T and HCT116 cell lines. AP-MS features from Guruharsha et al.^60^ and Malovannaya et al.^61^ were represented by HGSCore value and MEMOs value (mapped to “approved” = 10, “provisional” = 3, and “temporary” = 1), respectively. Hein et al.^23^ AP-MS data included prey.bait.correlation, valid.values, log10.prey.bait.ratio, and log10.prey.bait.expression.ratio. Features from Boldt et al.^24^ included the socioaffinity index (SA_*ij*_), spoke model indices (S_*ij*_, S_*ji*_) and matrix model socioaffinity index (M_*ij*_). Proximity labeling features representing single experiments from Youn et al.^26^ and Gupta et al.^27^ include average spectra, average saint probability, max saint probability, fold change, and Bayesian FDR estimate. Proximity labeling features were ultimately removed during feature selection of the classifier training workflow as they provided no predictive signal (see below). The full feature matrix can be found at https://humap3.proteincomplexes.org/static/downloads/humap3/humap3_20220625.featmap.gz. Descriptions of each feature can be found in Supplementary Data 11.

**Table 3:** Datasets

Dataset	Number of experiments	Links
Wan et al.^18^	5344 fractions	https://zenodo.org/records/15293715/files/humap2_feature_matrix_20200820.featmat.gz?download=1
Hein et al.^23^	1125 bait pull-downs	https://zenodo.org/records/15293715/files/humap2_feature_matrix_20200820.featmat.gz?download=1
Gupta et al.^27^	2 conditions × 58 proximity labeled baits = 116	https://zenodo.org/records/15293715/files/humap2_feature_matrix_20200820.featmat.gz?download=1
Youn et al.^26^	119 proximity labeled baits	http://www.cell.com/cms/attachment/2118963855/2087347233/mmc2.xlsx
Boldt et al.^24^	217 bait pull-downs	https://static-content.springer.com/esm/art%3A10.1038%2Fncomms11491/MediaObjects/41467_2016_BFncomms11491_MOESM834_ESM.xlsx
Treiber et al.^25^ (reprocessed by Mallam et al.^72^)	3004 RNA hairpin pull-down MS runs	https://www.cell.com/cms/10.1016/j.celrep.2019.09.060/attachment/c1395ea9-6837-4266-807d-c90f6be11328/mmc4.csv
Bioplex 3.0^20^ (includes Bioplex 2.0^21^ and 1.0^22^)	10,128 bait pull-down HEK 293T, 5522 bait pull-downs HCT 116	https://bioplex.hms.harvard.edu/data/BioPlex_293T_Network_10K_Dec_2019.tsvhttps://bioplex.hms.harvard.edu/data/BioPlex_HCT116_Network_5.5K_Dec_2019.tsv

### Weighted matrix model calculation

Weighted matrix model (WMM) features were calculated on Bioplex1^22^, Bioplex2^21^, Bioplex3^20^ (HCT116, Hek293T, and HCT116+Hek293T combined), Treiber et al.^25^, Hein et al.^23^, Gupta et al.^27^, and Youn et al.^26^. The full method was previously described in our hu.MAP^32^ study, as well as in Hart et al.^62^. The WMM is based on the hypergeometric test and asks, given two proteins A and B, whether they are seen in the same set of experiments more often than random chance using Eq. 1, where *k* = # of experiments both proteins, A and B, are seen together, *n* = # of experiments protein A is seen, *m* = # of experiments protein B is seen, and *N* = total # of experiments in the dataset. For each pair of proteins, a *p*-value is produced, which is then mapped to a negative logarithmic function (−log(*p*-value)), such that low *p*-values correspond to high-confidence interactions. In addition, we include a feature that summarizes the number of experiments each protein pair shares in a dataset (pair_count).

1 $$p\left({{{\rm{\#shared\,experiments}}}}\ge k | n,m,N\right)={\sum }_{i=k}^{\min \left(n,m\right)}\frac{\left({n}\atop{i}\right)\left({N-n}\atop{m-i}\right)}{\left({N}\atop{m}\right)}$$

The WMM for Bioplex 3.0 was calculated for the HEK293 experiments and HCT116 experiments individually, as well as collectively for the two cell type experiments combined. We used two thresholds (Bioplex Z-score > 2 and Z-score > 4) to discern whether an individual protein was present in an experiment and then calculated the WMM (e.g., feature name: neg_ln_pval_bp3_HCT116_Z4).

### Clustering method to create a benchmark test and a training dataset

To construct our benchmark, we developed a greedy clustering method to ensure there would be no data leakage between the test and training sets. Our method removes overlap of proteins as well as complexes, so the test and training sets are fully disjoint. First, we retrieved the Interactome3D (Ver. 2020_05) *Homo sapiens* table, and then filtered for entries with associated experimental PDBs (i.e., labeled “structure” in the type column). Interactome3D^31^ identifies physically interacting pairs of proteins from three-dimensional structures in the Protein DataBank^30^, and collates them into “interactions.dat” data tables, giving high-level summaries for each pair, where an “entry” corresponds to a single row in this table.

We then group Interactome3D entries based on their parent complexes (defined by PDB id) and calculate a matrix of Jaccard indices for all complex pairs. Jaccard index is defined as J(X, Y) = |X ∩ Y|/|X ∪ Y|, where X and Y are sets of protein subunits in different protein complexes. Complexes were then clustered using Seaborn ClusterMap^63^, a hierarchical clustering approach, based on the Jaccard index. From the hierarchical clustering, a single cluster is selected as a seed where all complexes within the cluster have a Jaccard index > 0. The seed cluster is then expanded to include additional complexes that have a Jaccard index > 0 to any complex in the seed cluster. If new complexes are added to the seed cluster, the process is repeated until all overlapping complexes are included in the cluster. The overall process iterates by clustering the remaining complexes using Seaborn ClusterMap to choose a new seed cluster, followed by adding overlapping complexes. The success of this method relies on the assumption that the Jaccard index matrix of all pairwise complex comparisons is sparse, where few complexes overlap with other complexes, which is true for this case. Otherwise, if the Jaccard index matrix is dense, a node removal approach would be necessary, reducing the size of an already limited dataset. Pseudocode can be found in Supplementary Note 1.

This resulted in 5 clusters of complexes whose constituent pairs were completely unique to that cluster. The largest cluster was used as our training set, and the smaller four were combined and used as our leave-out set. See Supplementary Data 6 for a comprehensive list of positive test and training pairs. See our GitHub repository for an implementation of the code (https://github.com/KDrewLab/DirectContacts2_analysis/blob/main/machine_learning/DirectContacts2_building_benchmark_from_interactome3D.ipynb).

To label our feature matrix during training, we only considered protein pairs from complexes with ≥3 subunits to avoid trivial cases of dimers, which are guaranteed to only have a single, direct interaction. Negative labels in the training set were defined as all pairs of proteins in the set not labeled as positive, including co-complexing pairs that do not share an interface, as well as pairs from different complexes. To gain better insight into true performance, we constructed a more rigorous leave-out test set, which included only complexes with ≥5 subunits. This nearly eliminates the trivial cases where all subunits in a complex share direct interfaces with all others. Also, labels of test negatives were defined to be only co-complexing protein pairs that do not share an interface within a complex. This contrasts with the training set, as it excludes negatives between separate complexes, and, therefore, is a more stringent test. Finally, we only considered PDB structures with ≥80% sequence coverage in the structure. This ensures that protein pairs with limited structural coverage are truly indirect interactions, rather than potentially interacting through unresolved protein domains not reflected in their structures. In total, the training set consisted of 2177 positive labels from 595 complexes, of which 1472 had entries in our feature matrix and 100,707 negative labels, of which 47,246 had entries in our feature matrix (Table 1). Training entries with no values in the feature matrix were not used during training. Training entries with partial evidence in the feature matrix were included with NaNs used for missing features as recommended by AutoGluon. Many AutoGluon classifiers have native support for NaNs, including LightGBM, the final chosen model for DirectContacts2. The test set consisted of 638 positive labels from 50 complexes, of which 572 had entries in our feature matrix and 898 negative labels, of which 785 had entries in our feature matrix. All test entries are included in the evaluation to properly estimate the total recall. Entries with no values in the feature matrix are given a confidence score = 0.0 +  np.random.random()/1000000. To test for bias in our model, we additionally created a test set with expanded negative labels to include non-interacting pairs from separate complexes (Table 1, intra- and inter-complex). For a complete list of training and test entries, see Supplementary Data 1–6.

### Machine learning framework to discriminate between direct and indirect interactions

To train our classifier, we used AutoGluon’s tabular predictor with a binary classification scheme^35^. For training, we used our feature matrix (described above) of 324 experimentally derived features representing proteomic evidence of direct physical interaction. Labels for training were derived from Interactome3D as described above, and we set AutoGluon’s evaluation function to be “accuracy,” using default values for all other parameters. We empirically observe that training AutoGluon classifiers using LightGBM with “accuracy” and “balanced_accuracy” produced equivalent models (i.e., same model parameters and weights). AutoGluon’s fit function performs training and hyperparameter optimization for 13 different classifier architectures (Supplementary Data 7). Within Autogluon’s automated workflow, several features were deemed insufficiently predictive and were excluded from further evaluation, specifically: “AvgSpec,” “AvgP,” “MaxP,” “Fold_Change,” “BFDR,” “AvgSpec_nonciliated_bioid,” “AvgP_nonciliated_bioid,” “MaxP_nonciliated_bioid,” “Fold_Change_nonciliated_bioid,” “BFDR_nonciliated_bioid.”

An additional 6 features were deemed to carry no information (i.e., had insufficient training data to direct learning) and were ignored by the model: “Hs_helaN_ph_hcw120_1_pq_euc,” “Hs_helaN_ph_hcw120_2_pq_euc,” “Hs_helaN_ph_saf48_pq_euc,” “Hs_helaN_ph_tcs375_P1_apex,” “Hs_helaN_ph_tcs375_P2_pq_euc,” and “Hs_helaN_ph_tcs375_P3_pq_euc.” After training, the top-ranked base classifier was the LightGBM model. AutoGluon’s ensemble model (i.e., WeightedEnsemble_L2) did not improve accuracy over the base LightGBM model. For a performance comparison of all AutoGluon models, see Supplementary Data 7.

The final model was used to predict confidence scores (i.e., DirectContact scores) of 25,273,872 protein pairs for their probability of interacting directly (Supplementary Data 8). To construct our complex filtered network, we filtered our total set predictions based on pairs also found in complexes from ComplexPortal^37^, CORUM^36^, and hu.MAP3.0^28^ producing 191,011 DirectContacts2 pairs found in literature, curated or computationally predicted protein complexes (Supplementary Data 9).

### Precision recall analysis

Precision recall curves were calculated using the prcurve.py script in the protein_complex_maps package (https://github.com/KDrewLab/protein_complex_maps/blob/master/protein_complex_maps/evaluation/plots/prcurve.py [Commit: 3ebfb06]) using parameters --add_tiny_noise and --complete_benchmark. The script uses the Python scikit-learn package^64^ precision_recall_curve function and the matplotlib package^65^ for visualization. The model performance is evaluated on the leave-out set, consisting of 638 direct contacts (Supplementary Data 3) and 898 indirect contacts (Supplementary Data 4). Additionally, the recall comparison consists of all positive pairs in the leave-out set, not just those with data in the feature matrix. All networks were compared to the same leave-out set. We compared our pre- and post-complex-filtered DirectContacts2 network as well as the original DirectContacts^19^ (10.1371/journal.pcbi.1005625.s003), hu.MAP2.0^17^ (https://zenodo.org/records/15293715/files/humap2_ppis_ACC_20200821.pairsWprob.gz?download=1), hu.MAP3.0^28^ (https://humap3.proteincomplexes.org/static/downloads/humap3/humap3_all_ppis_202305.pairsWprob.gz), HuRI^29^ on a precision–recall basis. We additionally compare to randomly shuffled predictions labeled as “Random” in the plot. HuRI was not utilized in the generation of the DirectContacts2 network and was used only for evaluation purposes. HuRI was downloaded from http://www.interactome-atlas.org/data/HuRI.tsv. HuRI interactions do not have an associated confidence score, so as done in hu.MAP2.0^17^ for precision recall analysis, HuRI interactions were ranked based on the number of separate assays in which the interaction was identified. We also evaluated our model using precision recall analysis on a modified set of negatives that included additional negatives from inter-complex indirect contacts (i.e., pairs of proteins in separate complexes) totaling 41,622 pairs (Supplementary Fig. 2B, and see also Table 1). The inputs to the prcurve.py script were identical to above, except for the --input_negatives parameter using the set of negatives from Supplementary Data 5, rather than from Supplementary Data 4.

### Calculation of precision, recall, F1, MCC, FDR, and ROC

To evaluate the performance of our chosen confidence thresholds for DirectContacts2 (DirectContacts2 score ≥ 0.7 and DirectContacts2 score ≥ 0.9) against other networks, we performed a top-k rank-based evaluation across each network for precision (Eq. 2), recall (Eq. 3), F1 (Eq. 4), MCC (Eq. 5), and FDR (Eq. 6) (TP = true positive, FP = false positive, TN = true negative, FN = false negative). For each cutoff, we obtained the number of predictions in the test set (i.e., *k* = 83 for ≥0.9; *k* = 292 for ≥0.7) and use these as the top-k predictions. We then take the top-k predictions which overlap with the test set for other networks in Table 2. For calculating the ROC in Supplementary Fig. 2A, we used true positive rate (Eq. 7) and false positive rate (Eq. 8).

2 $${{{\rm{Precision}}}}=\frac{{{{\rm{TP}}}}}{{{{\rm{TP}}}}+{{{\rm{FP}}}}}$$

3 $${{{\rm{Recall}}}}=\,\frac{{{{\rm{TP}}}}}{{{{\rm{TP}}}}+{{{\rm{FN}}}}}$$

4 $${{{\rm{F}}}}1=\,\frac{2*{{{\rm{Precision}}}}*{{{\rm{Recall}}}}}{{{{\rm{Precision}}}}+{{{\rm{Recall}}}}}$$

5 $${{{\rm{MCC}}}}=\frac{{{{\rm{TP}}}}*{{{\rm{TN}}}}-{{{\rm{FP}}}}*{{{\rm{FN}}}}}{\sqrt{\left({{{\rm{TP}}}}+{{{\rm{FP}}}}\right)({{{\rm{TP}}}}+{{{\rm{FN}}}})({{{\rm{TN}}}}+{{{\rm{FP}}}})({{{\rm{TN}}}}+{{{\rm{FN}}}})}}$$

6 $${{{\rm{FDR}}}}=\frac{{{{\rm{FP}}}}}{{{{\rm{TP}}}}+{{{\rm{FP}}}}}$$

7 $${{{\rm{TPR}}}}=\frac{{{{\rm{TP}}}}}{{{{\rm{TP}}}}+{{{\rm{FN}}}}}$$

8 $${{{\rm{FPR}}}}=\frac{{{{\rm{FP}}}}}{{{{\rm{FP}}}}+{{{\rm{TN}}}}}$$

### Characterization of feature support for DirectContacts2 predictions

To identify the features with the strongest signal for discriminating direct from indirect interactions, we performed precision–recall analysis using the raw, untrained scores from each individual feature. Raw feature scores were evaluated against the training set interactions, with inter-complex negatives excluded from the set. Three iterations of randomly shuffled benchmark pairs were used to generate a random comparison. Features were then ranked by area under the precision–recall curve, and the top 20 features are used for further analysis and shown in Supplementary Fig. 4A.

To determine whether these identified top 20 features support high-confidence DirectContacts2 predictions, we evaluated their presence across predicted pairwise interactions at different DirectContacts2 score thresholds. This analysis was performed using the set of all DirectContacts2 predictions (Supplementary Data 8). For each of the top 20 features, we defined high feature support as a value within the top 25% of that feature’s score distribution across all predictions. This allowed us to distinguish pairs with any signal for a given feature from pairs with high-scoring feature support. For several DirectContacts2 score thresholds (e.g., 0.9, 0.7, 0.5, 0.3, 0.1, and all predictions), we calculated the fraction of predicted pairs that have high-scoring feature support. In general, we see that pairs with higher DirectContacts2 scores have a greater enrichment of high-scoring support from individual top-ranking features (Supplementary Fig. 4B).

We next asked whether support from multiple orthogonal feature classes was associated with higher DirectContacts2 scores. All features in the feature matrix were divided into four feature classes: CF-MS, AP-MS, weighted matrix model features for proximity label data (proximity-WMM), and weighted matrix model RNA pulldown (RNA-WMM). Since raw proximity labeling features were not considered in DirectContacts2 training, they were excluded from this analysis. Notably, no predicted pairs with a DirectContacts2 score ≥ 0.2 were supported only by raw proximity labeling data. We examined all predicted pairs with a DirectContacts2 score ≥ 0.2 from the raw network (Supplemental Data 8). For each pair, we identified features with non-NaN values and mapped those features to the four feature classes as described above. Pairs were then grouped by the number of feature classes represented, and their score distributions were evaluated using density plots (Supplementary Fig. 4C).

### Comparison to RoseTTAFold2-PPI network

To compare DirectContacts2 to RoseTTAFold2-PPI^15^, we used the RoseTTAFold2-PPI benchmark, which distinguished weak interactions from strong interactions (Zhang et al. Supplementary Data 2, downloaded from https://conglab.swmed.edu/humanPPI/downloads/benchmarks.tar.gz). The positives in this set (“pairs_partitioned_by_interface_sizes”) were determined by identifying human protein interactions (1) found in STRING, BioGRID, or Uniprot databases, (2) found similar sequences to the pair (>95% sequence identify, >90% coverage) in the PDB, and (3) pair had >5 residue pairs <6 Å (see Zhang et al. for details). The negative set used in Zhang et al. was found in the “positives_and_negatives.tsv” file, which was originally constructed by randomly sampling protein pairs (see Zhang et al. for details). To ensure no data leakage between the DirectContacts2 training set and the RoseTTAFold2-PPI test set, we removed all pairs that overlapped between the two. This resulted in the removal of 370 entries from the “strong” set, resulting in 1412 total entries, 475 entries from the “medium” set, resulting in 3194 entries, 17 entries from the “weak” set, resulting in 247 entries, and 5 entries from the negative set, resulting in 29,995 entries. As done in Zhang et al., when calculating precision, we weighted true positives by 0.021 (strong), 0.009 (medium), and 0.121 (weak), which results in a signal-to-noise ratio of 1:1000 (i.e., precision = TP × wp/(TP × wp + FP), where wp is a weight parameter; the recall calculation stays the same).

To determine the overlap between DirectContacts2 confident interactions and RoseTTAFold2-PPI confident interactions, we downloaded RoseTTAFold2-PPI final predictions (https://conglab.swmed.edu/humanPPI/downloads/final_predictions.tar.gz) and used the “final_predictions_80.tsv” file, which contains predictions with an expected precision of 80%. For comparison, we utilized the complex filtered DirectContacts2 predictions with a score > = 0.7.

### Comparison to AlphaFold2 modeled interactions

To evaluate DirectContact2’s ability to inform complex structural modeling, we compared our predictions to confidence values of AlphaFold2/AlphaFold2-Multimer-modeled structures downloaded from Burke et al.^13^ and Jänes et al.^12^. AlphaFold2 models and confidence scores (i.e., pDockQ values) were downloaded from (https://archive.bioinfo.se/huintaf2/) and (https://ftp.ebi.ac.uk/pub/databases/ProtVar/predictions/interfaces/2024.05.28_interface_models_high_confidence.tar, https://ftp.ebi.ac.uk/pub/databases/ProtVar/predictions/interfaces/2024.05.28_interface_models_low_confidence.tar). We recalculated pDockQ values for Jänes et al. models using the pDockQ.py script (https://gitlab.com/ElofssonLab/FoldDock/-/blob/main/src/pdockq.py) using the default interface distance cutoff of 8 Å. This resulted in a final set of 277,807 AlphaFold2 and AlphaFold-multimer models with associated pDockQ scores. Here we tested the complex filtered DirectContacts2, DirectContacts, hu.MAP2.0, hu.MAP3.0, and HuRI networks on their ability to rank the set of pairs on their likelihood to interact. Due to differences in each network’s confidence scores, we calculated the fraction of pairs with pDockQ scores ≥ 0.23 out of each network’s top-ranked *K* predictions, for *K* between 500 and 25,000 in increments of 500, generating a fraction-confident-at-*K* curve (Fig. 4A). The same analysis was performed at a pDockQ score threshold of ≥0.5 (Supplementary Fig. 3B).

### Generation of AlphaFold3 models

To assess DirectContacts2’s utility in guiding structure predictions in practice, we modeled 2573 pairs with a DirectContacts2 score ≥ 0.5 in AlphaFold3. 366 were generated using the AlphaFold Server (alphafoldserver.com)^7^, and 2207 using the official public release of AlphaFold3 (github.com/google-deepmind/alphafold3) installed locally. AlphaFold3 predictions included only the two peptide chains comprising a given pair in their structures, with no post-translational modifications. AlphaFold Server runs were performed without manual seeding, equivalent to selecting a random integer as a seed. We used a model seed of 1 for structures predicted locally. For local predictions, the multiple sequence alignments and model inferences were performed separately utilizing AlphaFold3’s --norun_inference and --norun_data_pipeline flags to optimize resource usage across CPU and GPU environments. Aside from this, local AlphaFold3 runs were performed with default settings. Computation for local runs was performed on Indiana University’s Jetstream2 HPC cluster, for which resources were granted via the NSF Access program.

For each pairwise structure prediction, pDockQ was evaluated using the same methodology and parameters discussed above. We then used this set of pairs to produce the analysis shown in Fig. 4B, where we show the fraction of pairs with pDockQ scores ≥0.23 at different thresholds of DirectContacts2 score.

### Relationship between structural modeling confidence and intrinsic disorder

To compare protein disorder content and AlphaFold3 modeling confidence, we used high-confidence DirectContacts2 pairs (DirectContacts2 score ≥ 0.9) with AlphaFold3 models. We used Pearson correlation between pDockQ of the structural models and the average fraction of disorder (DisProt database (v. 2025_06)^66^) between the two proteins in the pair (Supplementary Fig. 3D). Only pairs with annotations for both proteins were considered when determining correlation.

### Inter-protein cross-links comparison analysis

To evaluate DirectContacts2 predictions with respect to protein crosslinking data, we compared DirectContacts2 predictions to inter-protein cross-links identified in Wheat et al.^40^ (Dataset_S02, sheet “Dataset S2A,” link: https://www.pnas.org/doi/suppl/10.1073/pnas.2023360118/suppl_file/pnas.2023360118.sd02.xlsx). Cross-linking mass spectrometry (XL-MS) captures covalently linked residues between protein chains in close spatial proximity, providing strong experimental evidence of direct physical contact within multi-protein assemblies. Multiple cross-links per protein pair were treated as a single cross-link identification between the protein pair, creating a non-redundant set with 1 cross-link per protein pair. Finally, to ensure cross-linked protein pairs are inter-protein links, identified peptides for each protein in the pair were compared to the other protein in the pair. If a match was found, this suggested a possible self-link and the cross-link pair was excluded from further analysis.

To compare the DirectContacts2 and hu.MAP3.0 networks on their agreement with cross-linked pairs, we compared each set to the crosslink dataset. For this analysis, we utilized the top ranking 25,000 confident interactions from our complex filtered DirectContacts2 network (Supplementary Data 9), and 25,000 most confident hu.MAP3.0 pairwise predictions. To account for differences in prediction coverage between networks, we only considered interactions involving the 1329 proteins common among all three sets (i.e., DirectContacts2, hu.MAP3.0, Wheat et al.). We further limited interactions from these proteins to only those in protein complexes. Specifically, we determined the total set of pairs from the 1329 proteins that were known or predicted to co-complex per literature-curated databases (i.e., CORUM^36^, ComplexPortal^37^, and hu.MAP3.0^28^), resulting in 12,086 possible pairings for this common set of proteins. Intersecting this universe of pairs with the Wheat X-linked pairs results in the set of possible 229 X-linked pairs involving proteins for which DirectContacts2 and hu.MAP3.0 makes predictions. Intersecting the universal set with the DirectContacts2 and hu.MAP3.0 networks show they make 1418 and 2239 total predictions in this universe of pairs, respectively. From these pairs, DirectContacts2 identified 127 XL-MS pairs out of its 1418 predictions and hu.MAP3.0 identified 123 XL-MS pairs out of its 2239 predictions.

For both DirectContacts2 and hu.MAP3.0, we then sampled a random 229 co-complex pairs of proteins from the non-redundant cross-link dataset. Co-complex pairs were defined as pairs of proteins present in hu.MAP3.0, CORUM, or ComplexPortal. The random samples were then intersected with the sets of pairs from each high-confidence network. This sampling and intersection process was repeated 1000 times to generate background distributions representing the tendencies to predict cross-linked protein pairs by random chance. Finally, we used the previously calculated intersection sets of both networks with the XL-MS set of pairs to estimate each network’s enrichment for cross-linked protein pairs against their random backgrounds, obtaining Z-scores representing each network’s enrichment for cross-linked pairs over random chance.

### Agreement of AlphaFold models and inter-protein cross-links

AlphaFold2 models of protein interactions HYOU1-HSPA5 and TMED5-TMED10 were used from Burke et al.^13^. AlphaFold-multimer models of NEFL-NEFM and NEFM-VIM were created using the “colabfold_batch” command line script developed as part of ColabFold^67^. For each protein pair, we downloaded FASTA sequences from Uniprot, constructed input files, and ran colabfold_batch with the following parameters: “--templates --num-recycle 3 --use-gpu-relax --model-type alphafold2_multimer_v3.” Colabfold_batch code was downloaded from the localcolabfold GitHub repository (https://github.com/YoshitakaMo/localcolabfold). To generate higher stoichiometry models of NEFM-VIM interaction, we created an AlphaFold3^7^ model with default settings of expected overlapping segments (based on crosslinking data), specifically NEFM segments 85–176, 181–349, 350–498 and VIM segments 85–175, 176–338, 363–466 (https://zenodo.org/records/15634862/files/AF3_fold_nefm_vim_tails12_heads_2024_05_23_13_21.zip?download=1). This, therefore, restricts prediction to the expected dimer-of-dimer interface. We then aligned in PyMol^68^ two copies of the original 1:1 stoichiometry NEFM-VIM model onto the dimer-of-dimer interface model (https://zenodo.org/records/15634862/files/NEFM_VIM_higherorder_20240529.cif?download=1). Crosslinks were visualized using PyXlinkViewer^69^. PyXlinkViewer crosslink files are also available from the Zenodo repository.

### Calculation of pdockQ scores

To assess AlphaFold-multimer predictions, we calculated the pDockQ score using the pdockq.py script downloaded from https://gitlab.com/ElofssonLab/FoldDock/-/blob/main/src/pdockq.py. The script takes the PDB output from AlphaFold-Multimer and uses the plDDT of the interface residues to evaluate the likelihood of interaction.

### Orofacial digital syndrome complex

Mutations for OFD1 were identified using the Uniprot variant viewer^70^, which were UniProt reviewed and labeled “pathogenic.” The orofacial digital syndrome (OFDS) complex was identified in hu.MAP2.0 (id: HuMAP2_00328, http://humap2.proteincomplexes.org/displayComplexes?complex_key=HuMAP2_00328). Pairs from the OFDS complex were ranked by DirectContact2 scores, where OFD1-FOPNL and FOPNL-KIAA0753 were the highest-scoring pairs within the complex. We used “localcolabfold” (i.e., AlphaFold-multimer) to predict three-dimensional structural models for these high-scoring protein pairs. The pDockQ score was calculated to evaluate the likelihood of interaction based on the AlphaFold-multimer structures. Pathogenic mutations of OFD1 were mapped on the AlphaFold structure in PyMOL^68^. The trimer structural model of OFD1-FOPNL-KIAA0753 was created by aligning the common subunit of the dimer models (i.e., FOPNL) in PyMOL.

### Analysis of the evolutionary age difference between directly interacting proteins

Our evolutionary analysis used evolutionary ages defined in Liebeskind et al.^55^. This dataset associates human genes with a probability mass function (PMF, i.e., vector of probabilities for each age) over eight numbered age bins: 0: *Cellular organisms*, 1: *Eukarya+Archaea*, 2: *Eukarya+Bacteria*, 3: *Eukaryota*, 4: *Opisthokonta*, 5: *Eumetazoa*, 6: *Vertebrata*, 7: *Mammalia*. We first mapped each protein in the 6967 high-confidence complex filtered DirectContacts2 pairs (Supplementary Data 12; DirectContacts2 score ≥ 0.9) to its age PMF from Liebeskind et al. and discarded pairs with proteins that did not map. This resulted in a set of 6844 highly confident direct pairs where each protein has an age PMF.

We next compared the ages of DirectContacts2 pairs to a random background. We created the random background by shuffling the second protein column (i.e., “uniprot_id2” column) of the high-confidence set, preserving the same protein set while shuffling the pairs. To determine whether proteins in predicted direct pairs exhibit similar evolutionary age (i.e., lower age index difference), we ran a permutation analysis on the difference of means between the highly confident and background distributions for pairwise age index difference (i.e., Δ = *μ*_BG_ − *μ*_CONF_, where *μ* denotes the sample mean of absolute age index difference ∣*a*_i_ − *a*_j_∣ between proteins *i* and *j*, in a pair). Significance was determined using SciPy’s implementation of the permutation test with 40,000 resamples (i.e., *n*_resamples = 40,000) and an alternative hypothesis that the mean age difference of background pairs is greater than that of pairs in the highly confident set (i.e., alternative = “greater”). We then performed manual bootstrapping of pairwise mean age index difference for pairs from the highly confident and background sets. Five hundred random samples of 1000 pairs were drawn from both sets, and the average pairwise age index difference was plotted for each sample (Fig. 7A).

To compare the mean age index of direct pairs to the age of their associated complexes, we obtained average age indexes for the pair and the pair’s complex (i.e., “complex_acc” column, Supplementary Data 9). For any set of proteins (e.g., pair, complex), we define the average age index as the expected value of the average PMF of all protein PMFs in the set. For pairs associated with multiple protein complexes, a random complex was chosen. Taking the difference of the pair’s average age index with its chosen complex’s average age index yields a discrete distribution. Averaging over 500 of the above trials gives the continuous distribution of mean pair-complex age index differences plotted in Fig. 7B. Pairwise age values can be found in Supplementary Data 12.

### Reporting summary

Further information on research design is available in the Nature Portfolio Reporting Summary linked to this article.

## Supplementary information

Supplementary Information

Description of Additional Supplementary Files

Supplementary Data 1

Supplementary Data 2

Supplementary Data 3

Supplementary Data 4

Supplementary Data 5

Supplementary Data 6

Supplementary Data 7

Supplementary Data 8

Supplementary Data 9

Supplementary Data 10

Supplementary Data 11

Supplementary Data 12

Reporting Summary

Transparent Peer Review File

## Source data

Source Data

## Data Availability

The Autogluon model can be found on HuggingFace: 10.57967/hf/9294. We provide the full feature matrix and all test and training feature matrices on huggingface: 10.57967/hf/9295 under CC4.0 license. AlphaFold structural models can be found on Zenodo (10.5281/zenodo.15634861) and are available in ModelArchive^71^ (modelarchive.org) with the accession code “ma-drew-dc2” (10.5452/ma-drew-dc2). Data shown in Supplementary Fig. 2I was downloaded from PRIDE: PXD002321. (Co-fractionation experiments in *D. melanogaster*). Source data are provided with this paper.

## Code availability

All code can be found at our GitHub repository: https://github.com/KDrewLab/DirectContacts2_analysis.git (version 5ddb90c) under the MIT license or https://github.com/KDrewLab/protein_complex_maps (Commit: 3ebfb06) also under the MIT license. Code is also available and archived through CodeOcean: https://codeocean.com/capsule/5754597/tree.
