# Supplementary material for: DirectContacts2: a wiring diagram of human physical protein interactions
Source: Nat Commun. 2026 Jul 24;17:9037. doi: 10.1038/s41467-026-75863-3 (PMC13507227; doi:10.1038/s41467-026-75863-3)
Supplement: Supplementary file 2 [file 41467_2026_75863_MOESM16_ESM.pdf]

nature portfolio

Peer Review File

DirectContacts2: A wiring diagram of human physical protein interactions

Corresponding Author: Dr Kevin Drew

This file contains all reviewer reports in order by version, followed by all author rebuttals in order by version.

Version 0:

Reviewer comments:

Reviewer #1

(Remarks to the Author)
The manuscript from Claussen et al. develops a machine learning model to distinguish directly interacting protein–protein interactions (PPIs) from indirectly associated proteins. The model is trained on features derived from a variety of mass spectrometry (MS)–based experiments, including co-fractionation, proximity labeling, and large-scale AP–MS studies such as BioPlex. Labels for the training set were assigned using experimentally determined PDB structures of multi-protein assemblies.

Unfortunately, the overall strategy appears conceptually flawed. A binary classification is insufficient to describe the underlying biochemical reality of protein interactions. In the MS-based input datasets used here, transient but direct interactions are often less strongly correlated than stable, indirect associations. For example, in co-fractionation or AP–MS experiments, weak or transient direct interactions may produce lower correlation profiles or peptide counts than constitutive complex members that do not directly contact each other. Among the MS techniques considered, only crosslinking mass spectrometry (XL–MS) provides unambiguous evidence for direct PPIs, yet in this study, XL–MS data are used only for validation, not model training.

This issue is illustrated clearly in Figure 3. The CCT/TRiC complex consists of eight subunits arranged in a double-donut structure. These 16 subunits form a constitutive assembly and are not observed as individual subunits or sub-complexes. Consequently, AP–MS using a single subunit as bait retrieves all subunits at high abundance, even though each subunit directly contacts only a few partners (e.g., CCT2 contacts CCT4, CCT5, and the second copy of CCT2). The DirectContacts2 (DC2) scores for CCT2–CCTx pairs are all >0.5, and in many cases, indirect pairs score higher than the direct ones (e.g., CCT2–CCT7: 0.941; CCT2–CCT4: 0.629). Figure 3 highlights only the eight highest-scoring pairs, which obscures this pattern. Moreover, the figure depicts a super-assembly of two constitutive complexes, prefoldin (PFDN) and CCT. The most informative evaluation would test whether the model predicts the inter-complex direct interactions (e.g., PFDN6–CCT4 and PFDN4–CCT4 in the corresponding PDB structure). Yet these have low DC2 scores (0.111 and 0.0105, respectively), suggesting that the model primarily distinguishes stable, constitutive associations from transient ones, rather than truly direct from indirect PPIs.

The proximity-labeling data for prefoldin (Figure S4A) also support this interpretation. While the authors highlight direct pairs such as PFDN3/2 and PFDN3/5, the same data show non-contacting subunits (e.g., PFDN6 and PFDN2) co-detected in multiple experiments, as well as instances where PFDN3 or PFDN2 appear without any partners. It is also unclear why PFDN4 is missing from this figure.

The global benchmarking further reinforces this view. The authors' prior hu.MAP3 model—trained on the same MS-derived features and datasets to predict co-complex membership, regardless of directness, performs comparably to DC2. The Precision–Recall plots (Figure 2 and Supplemental Figure 2C) and AlphaFold predicted interface comparisons (Figure 4A and Supplemental Figure 2D) show only modest improvements for DC2 over hu.MAP3. This marginal gain likely reflects that the MS-based features encode interaction strength or stability, not physical directness.

Supplementary Figure 2B does show some separation between direct and indirect test-pair score distributions, but the overlap remains substantial. The discriminative power appears too limited to inspire confidence in DC2 predictions for complexes of interest to most researchers.

Figure 5 evaluates DC2 using >5000 PPIs identified by XL–MS from the Huang lab. The results are presented as Z-scores relative to random protein pairs. However, this background consists of non-interacting proteins, not pairs from the same complex lacking direct contacts. As such, the separation between the two distributions does not truly test the ability to classify direct versus indirect PPIs. The same analysis applied to hu.MAP3 yields similar findings: DC2 scores are somewhat higher than huMAP3 scores, but the difference (e.g., 80 vs. 55 standard deviations from background) is not biologically meaningful given the inappropriate null model. A more informative analysis would compare to protein pairs selected from known protein-assemblies, but also it would be helpful to show a simple confusion matrix or contingency table comparing XL–MS–validated PPIs with DC2 predictions.

Overall, while a machine learning model capable of accurately predicting direct interactions would be a valuable advance, this work does not convincingly achieve that goal. The DC2 model appears to primarily reflect the stability or affinity of associations rather than physical contact per se. I also note that while the manuscript suggests that proteome-wide AlphaFold screening is computationally prohibitive, recent work indicates that large-scale pooled AlphaFold-based PPI prediction is indeed feasible (see bioRxiv 2025.07.01.662654v2).

In summary, the manuscript is clearly written and well-presented, but the model's conceptual basis and evaluation do not support the claim that it distinguishes direct from indirect PPIs. I would recommend major revision or rejection unless the authors can demonstrate improved benchmarking against known direct/indirect contacts that DC2 specifically captures direct physical interactions rather than general co-complex association strength.

(Remarks on code availability)

Reviewer #2

(Remarks to the Author)
Summary
The authors present DirectContacts2, which is a machine learning model designed to distinguish direct from indirect protein interactions using features derived from mass spectrometry-based proteomics data. The model's performance was evaluated against existing methods, demonstrating better precision and recall in predicting direct contacts. The authors further validated their predictions using structure prediction approaches and highlighted several structural modeling examples of disease-relevant protein complexes to illustrate the model's applicability.

Major comments:
1. There appears to be several issues with the setup of the framework. As noted in the main text, the training data consists of 2,177 positives and 100,707 negatives, resulting in a positive-to-negative ratio of 1:50. According to the methods section, the authors used accuracy as the evaluation function in the training process, which is not suitable for imbalance datasets and may fail to capture the model's actual performance in addressing the minority class. Furthermore, in the methods section, the authors mentioned that "the training set consisted of 2,177 positive labels from 595 complexes of which 1,472 had entries in our feature matrix and 100,707 negative labels of which 47,246 had entries in our feature matrix". This indicates that a substantial proportion of training data contains missing values. However, the manuscript does not clearly explain how these missing values were handled or how the model makes predictions for samples with incomplete features.
2. The methodological novelty of the DirectContacts2 framework appears limited. According to the methods section, it relies on the same features as the previous work hu.MAP3.0 and used the weighted matrix model established in the previous hu.MAP study. Although the authors showed that the new model achieved better precision-recall in predicting direct protein interactions, the authors may wish to add additional clarification about what specific improvements or innovations distinguish this framework from prior work.
3. In the "Integration of Proteomics Data Improves Discrimination Between Direct and Indirect Interactions" section, the authors state that the negative set consists of "100,707 known indirectly and non-interacting (i.e., non-co-complexing) pairs of proteins." However, in the "DirectContacts2 Network Outperforms Previous Networks on Leave-Out Set of Physically Contacting Protein Pairs" section, the authors mention that they "excluded the negative set pairs from separate complexes to provide a rigorous assessment of our model." These two statements appear somewhat inconsistent. While it is reasonable to evaluate model performance on distinguishing direct from indirect interactions within the same complex, including non-co-complexing pairs during training could make the classification task substantially easier and potentially introduce bias into the model. This point should be clarified.
4. In the "Integration of Proteomics Data Improves Discrimination Between Direct and Indirect Interactions" section, the authors report that they "selected a DirectContacts2 score threshold of ≥ 0.5," based on the score distribution shown in Supplementary Figure 2A. However, the rationale for choosing this threshold is not clearly explained. Specifically, in Supplementary Figure 2A, the small "bump" corresponding to higher DirectContacts2 scores occurs around 0.95, making it unclear why 0.5 was chosen as the cutoff. The authors should provide a justification for this threshold selection (e.g., whether it was empirically optimized, based on prior studies, or derived from precision-recall tradeoffs).
5. In the "DirectContacts2 Guides the Structural Model of a Human Ciliopathy Complex" section, the authors highlight the OFD1-PIBF1-FOPNL(CEP20, FOR20)-KIAA0753 complex as an example to demonstrate how DirectContacts2 can guide AlphaFold modeling. However, this complex has already been identified in prior datasets (e.g., hu.MAP2.0), which diminishes the novelty of this example and does not convincingly illustrate the unique contribution of DirectContacts2 in structural modeling. The authors are encouraged to present novel direct interactions or complexes uniquely discovered by DirectContacts2, ideally supported by independent functional evidence or orthogonal validation.
6. One of the main advantages of DirectContacts2 over structure prediction-based approaches (e.g., AF3) is its ability to

generate large-scale, all-by-all predictions of direct interactions across nearly the entire proteome. However, this strength is not sufficiently emphasized in the current manuscript. Much of the paper focuses on validating predicted interactions, many of which correspond to previously known interactions. To better highlight the novelty and distinct contribution of DirectContacts2, it would be valuable to include additional analyses that demonstrate its innovation, for example, performing some network-scale functional analyses.

Minor comments:
1. In Figure 1B, the “Benchmark” component (grey box) in the workflow diagram is somewhat confusing. The arrow connecting the “Benchmark” dataset (containing direct and indirect protein pairs) to the “Feature matrix” (grey-blue box) suggests that these data are used as input features. However, they should instead serve as ground truth labels. Additionally, please clarify how the number 5,252 in the “Benchmark” box was calculated. In the main text (line 129), it is stated that there are 2,177 known directly interacting pairs and 100,707 known indirectly and non-interacting (non-co-complexing) pairs, so the derivation of 5,252 is unclear.
2. In Figure 2, panels A and B appear redundant and could be combined into a single panel that shows both precision-recall curves and auPR values. The authors may consider adding ROC curves and auROC values for additional evaluation. Also, additional details are needed to clarify whether the methods for comparison were applied to the same set of protein pairs and how fair comparisons were guaranteed in the evaluation.
3. In the “Integration of Proteomics Data Improves Discrimination Between Direct and Indirect Interactions” section, the authors mentioned that they “filtered our network by known co-complex associations in hu.MAP3.0, CORUM, and ComplexPortal”. It is unclear, however, how the resulting network can then be fairly compared to the hu.MAP 3.0 network, given that hu.MAP 3.0 itself was used as part of the filtering process.
4. In the section “Enrichment of High Confidence Multichain AlphaFold2 Models in DirectContact2 Predictions”, the text states that “we calculate the fraction of each network’s most confident set of predictions with confident AlphaFold models (pDockQ ≥ 0.23) for increasing sizes of top-ranked pairs.” However, in Figure 4, the y-axis label is “pDockQ > 0.23”, which is inconsistent and should be unified.
5. In Figure 4A, the blue line representing “DirectContacts” is significantly shorter than the others. Does this indicate that “DirectContacts” predicts only ~2,500 pairs? Also, in the main text, please provide citations for all previously published methods used in benchmarking (e.g., DirectContacts, hu.MAP, etc.).
6. In Figure 4B, it is unclear what each dot represents; please clarify this in the text. Moreover, many yellow dots have high DirectContacts2 scores but low fractions of AF3 models with high pDockQ scores, suggesting that the most confidently predicted direct interactions by DirectContacts2 are not supported by AF3 models. This observation appears to contradict the argument presented in this section.
7. The legend next to Figure 5D is confusing. The blue and dark red edges represent “Satisfied Xlinks” or “Violated Xlinks” in the structure models from panels B/E/F, while the green edges indicate “Contacts supported by Xlinks” in the network shown in panel D. However, combining these legends next to panel D makes interpretation unclear. Consider separating or relabeling them for clarity.
8. In “DirectContacts2 guides the structural model of a human ciliopathy complex” section line 325, please explain the sources of the multiple OFD1 mutations and further provide the detailed information for these selected mutations (i.e., associations with any diseases; deleteriousness scores from variant effect prediction, etc.). It can make the statement “the disruption of this interface is responsible for the downstream functional defect giving rise to a clinical phenotype” stronger and convincing.
9. In Supplementary Figure 3, the y-axis tick labels are confusing. They appear to correspond to the feature names used in the model, but the current labels are difficult to interpret. Please rename them to improve readability and interpretability.
10. In Supplementary Figure 4, the colors assigned to Prefoldin subunits in panel A do not match those in panel B. Please either relabel the subunits in panel B or adjust the color scheme so that they correspond consistently across panels.
11. In “Precision Recall Analysis” of the Methods section, please use the full name of “sklearn” package (should be scikit-learn).

(Remarks on code availability)

Reviewer #3

(Remarks to the Author)
The authors present a machine-learning model for separating direct from indirect protein–protein interactions. This is an important distinction that is often ignored in constructing molecular network models based on proteomics data. The new model was trained on large-scale mass spectrometry datasets. The authors report that their model can separate direct from indirect protein–protein interactions more effectively than the existing methods. Thus, the method should be of interest to the scientific community, including systems biologists and structural biologists.
I have the following comments/questions for the authors:

1. The model is trained on features derived from mass spectrometry datasets and supervised using only the protein–protein interactions documented in the PDB. The model is then applied to the full set of ~25M human protein pairs. Because the model is trained on stable, crystallizable complexes, how applicable is it to identifying novel physical interactions that are transient, weak, and condition dependent? In other words, how much can DirectContacts2 generalize beyond the training set in the PDB?

2. The DirectContacts2 score distribution is bimodal, with a large peak near 0 (presumably false interactions) and a small peak near 1 (likely true interactions) (Supplementary Figure 2A). Authors have used 0.5 as the confidence cutoff, but this appears to have been an ad hoc choice. Is there a rationale supporting this choice? The authors should probably provide a threshold sensitivity analysis, reporting precision, recall, F1, MCC, and/or FDR at each possible cutoff. Without such an analysis, it is unclear if 0.5 is optimal in some sense; for example, one could argue based on the bimodal distribution that a higher threshold (eg, 0.7–0.9) might yield “better” predictions.

3. The AlphaFold validation shows that only about 50% of the highest-scoring predictions (DirectContacts2 > ~0.99) result in good structural models (pDockQ ≥ 0.23). This finding suggests that many predicted direct interactions, even with scores as high as 0.9, cannot currently be structurally modelled. It would be interesting to discuss such outcomes in terms of different kinds of structural modeling errors, intrinsically disordered regions, and other reasons.

(Remarks on code availability)

Reviewer #4

(Remarks to the Author)
I co-reviewed this manuscript with one of the reviewers who provided the listed reports. This is part of the Nature Communications initiative to facilitate training in peer review and to provide appropriate recognition for Early Career Researchers who co-review manuscripts.

(Remarks on code availability)

Reviewer #5

(Remarks to the Author)
I co-reviewed this manuscript with one of the reviewers who provided the listed reports. This is part of the Nature Communications initiative to facilitate training in peer review and to provide appropriate recognition for Early Career Researchers who co-review manuscripts.

(Remarks on code availability)

Version 1:

Reviewer comments:

Reviewer #1

(Remarks to the Author)
The manuscript revisions clarify and improve several technical aspects of the methodology that were raised by the reviewers, including the choice of confidence thresholds, balance between training and testing sets, the null model used for the CLMS benchmarking, and the description of the benchmarking procedures. These changes improve the clarity of the manuscript and make the evaluation of the method more transparent.

My primary concern remains that the experimental readouts used as input features are derived from MS-based proteomics experiments that do not clearly distinguish between interaction strength/stability and physical interaction directness. While the authors provide arguments that integrating many experiments can help extract such signals, I remain cautious about the interpretation that the model specifically captures direct physical contacts. For example, Figure 4A shows only modest improvement in recovering high-quality AF3 structural models relative to hu.MAP3, which is trained on essentially the same underlying data but without labels explicitly intended to represent direct contacts.

A similar pattern is seen in the CLMS benchmarking analysis. According to the revised methods section, 229 crosslinked protein pairs exist within the “universe” of interactions shared among the three datasets considered. DirectContacts2 identifies 127 of these pairs among its 1,418 predictions, while hu.MAP3 identifies 123 among 2,239 predictions. While DirectContacts2 shows somewhat stronger enrichment for crosslinked pairs than hu.MAP3, both methods still fail to recover a substantial fraction of the experimentally observed direct interactions (eg, the 229 crosslinked PPIs).

Minor, side point: it's not clear how they come up with the "universe" of 12,086 possible protein-pairs.

That said, the authors make reasonable points regarding the ability of machine learning models to extract signal across many experiments even when individual datasets are noisy or incomplete. The revised manuscript is also technically clearer and better contextualized.

Overall, I remain somewhat skeptical that a machine learning model trained primarily on MS-based association data can fully resolve interaction directness. However, the approach appears useful as a prioritization or ranking framework for candidate direct interactions, and the resulting resource is likely to be of interest to the community, particularly if the manuscript frames the method as enriching for likely direct interactions rather than definitively detecting them.

(Remarks on code availability)

Reviewer #2

(Remarks to the Author)
Summary:
This revised manuscript presents DirectContacts2, a machine learning framework that aims to distinguish direct from indirect protein-protein interactions using features derived from large-scale mass spectrometry datasets. Although the authors have clarified several points from the initial review, including benchmark construction, handling of missing values, and negative sampling rationale, the central methodological concerns remain unresolved, limiting confidence in the validity and significance of the reported findings.

Follow-up comments:
1. The main concern about novelty remains. DirectContacts2 appears to use essentially the same overall feature space as hu.MAP3.0 and related previous work. The main difference is the benchmark construction and the relabeling of the task from co-complex association to direct-versus-indirect interaction. However, both this extension and the results still seem incremental. Even though DirectContacts2 is trained specifically on direct-versus-indirect labels, the gains over hu.MAP2.0/3.0 are modest. In Table 1, the performance improvement is limited. In Fig. 4A and Fig. S3B for the comparison of enrichment for confident pair interfaces, especially for the top-ranked pairs that are often the most relevant in practice, hu.MAP3.0 is not clearly worse and in some regions looks comparable. This makes the practical advantage of the new framework less convincing. To summarize, the work still appears to be a modest reworking of the previous hu.MAP framework, without the level of methodological innovation or validation expected for a high-impact publication. Comparison only to earlier hu.MAP networks is not enough. If the authors wish to argue that DirectContacts2 represents a meaningful advance, it should be benchmarked not only against prior co-complex networks, but also against current ML- and structure-based PPI prediction methods such as AF2Complex, RoseTTAFold2-PPI, Boltz-2, TT3D, and other relevant recent baselines. Without these comparisons, the manuscript does not convincingly establish either the novelty or the practical advantage of the proposed method.

2. The clarifications regarding the “greedy clustering” and training objectives serve more to highlight the inherent limitations of the study than to mitigate them. The core technical contribution appears to be an incremental data-splitting strategy, essentially a standard search for connected components, which the authors overstate as a novel “greedy clustering” algorithm. The authors didn’t explain why other deduplication tools (e.g., CD-HIT, MMseqs2-based splits) are insufficient for this specific problem. In addition, this approach, combined with a highly limited test set of only 50 complexes and a training phase reliant on “easy” inter-complex negatives, raises serious doubts about the model’s ability to generalize to the broader human proteome. The provided pseudocode is not clear and contains several inconsistencies and apparent logical errors (e.g., incorrect stopping condition, ambiguous indexing of clustering outputs, and improper list operations). As written, it is difficult to reproduce the method, which undermines the transparency and reliability of the benchmark construction.

3. The authors' response regarding accuracy versus balanced accuracy relies on an unverified, framework-specific behavioral claim rather than principled reasoning. Throughout the manuscript, critical methodological decisions including model selection, training objective, missing value handling, and ensemble construction are justified primarily by deferring to the AutoGluon framework rather than by principled experimental design. Furthermore, in the revised manuscript and the response letter, the observation that “accuracy” and “balanced accuracy” yield identical models appears to be a consequence of the AutoGluon framework rather than a principled property of the learning problem. The authors seem to be unclear about the training objectives and evaluation metric in the model selection process. In other words, the training objective is not directly tied to the evaluation metric, and the role of the metric is primarily in model selection. The training objective for each binary classifier doesn’t seem to adjust the class imbalance according to the AutoGluon implementation. Therefore, this observation does not justify the use of accuracy in an imbalanced setting, nor does it demonstrate that class imbalance has been appropriately handled. Instead, it raises additional concern about the validity and correctness of the entire model training and evaluations. To summarize, the use of accuracy as the evaluation metric in a highly imbalanced setting is not adequately justified, and the observation that accuracy and balanced accuracy yield identical models appears to reflect framework-specific behavior rather than a principled result. Furthermore, the feature selection process is insufficiently described, and no explicit strategies are employed to address class imbalance.

4. In addition to the class imbalance issue, the internal cross-validation setting also seems to be problematic. There’s no enforced complex-level separation during the cross-validation step, therefore data leakage may be reintroduced at the feature and model selection stage, undermining the carefully constructed outer benchmark split. The model is subsequently applied to tens of millions of protein pairs without validation of generalization or calibration, and the final network is heavily filtered using existing complex databases, limiting the extent to which the results represent de novo predictions. Overall, the methodological rigor of the modeling pipeline falls short of the standards expected for a high-impact study.

5. The description of the final classifier is also somewhat misleading. The manuscript states that AutoGluon trained 13 base

models and then a WeightedEnsemble_L2 model using these base models. However, the authors also state that only the LightGBM base model received non-zero weight in the final WeightedEnsemble_L2. If so, the final predictor is effectively just the LightGBM model rather than a meaningful ensemble. While it is fine to note that an ensemble-selection procedure was attempted, the manuscript should describe the final deployed classifier more directly and avoid implying that predictions benefited from combining multiple base learners when the ensemble in practice collapsed to a single model.

6. We appreciate the authors for providing Table 3, but it also shows that the number of pairs actually used for model fitting is much smaller than the benchmark counts emphasized in the text, which is misleading. For example, the main text described the training set as having 2,177 positives and 100,707 negatives, but only 1,472 positives and 47,246 negatives appear to have feature-matrix entries and are actually used for training. The same issue applies to the test set. Pairs without feature-matrix entries are excluded from the model and should not be presented interchangeably with the effective training and test samples. The manuscript should explicitly report the number of PPIs with usable features that were actually used for training and evaluation, rather than foregrounding the larger benchmark totals in a way that overstates the amount of informative data available to the model.

7. The explanation for adding non-co-complexing pairs to the training set is understandable, given the limited number of within-complex indirect negatives. However, this still remains a concern. The biological question of interest is whether the model can distinguish direct from indirect interactions within complexes. Once a large number of clearly unrelated inter-complex pairs are added as negatives, the training problem becomes easier and shifts toward a broader distinction between associated and unrelated pairs. Even if the evaluation is later restricted to within-complex negatives, the training objective is no longer fully aligned with the biological claim.

8. In Fig. S2E, the overlap between the score distributions for direct and indirect pairs is still substantial. Since these pairs come from well-characterized PDB complexes, this is not a minor issue. If the model does not separate the two classes well even in this benchmark setting, it is difficult to be confident about performance on less well-supported pairs. This does not mean the model has no value, but it suggests that the paper is showing enrichment rather than strong discrimination. That is a more limited claim than the manuscript currently makes.

9. The manuscript continues to emphasize large-scale applicability, but there is still not enough guidance on what this means in practice. For example: how much feature support is needed before a prediction becomes reliable, how performance changes for pairs with sparse evidence, what fraction of the proteome is covered well enough for confident use, and how medium-confidence predictions should be interpreted. These questions are important for evaluating the real utility of the method from a user's perspective.

10. While DirectContacts2 shows a higher fraction of overlap with cross-linked pairs (127/1,418 vs 123/2,239), the absolute improvement is modest (~3.5 percentage points), yet corresponds to a disproportionately large increase in Z-score (20.5 vs 13.7). This suggests that the null distribution has very low variance, inflating statistical significance and potentially overstating the magnitude of improvement.

11. There is also a minor consistency issue with the score thresholds. Earlier sections define 0.7 and 0.9 as the lower- and higher-confidence cutoffs, but later in the "Intermolecular Cross-links are enriched in DirectContact2 Network" section, TMED5-TMED10 is discussed as a predicted direct interaction despite having a DirectContacts2 score of 0.64. This should be clarified.

12. Some of the figures are still underdeveloped for a paper at this level. Fig. 2 in particular remains rather thin as a main figure, and the inset bar plot is largely redundant with the PR curves.

(Remarks on code availability)

Reviewer #3

(Remarks to the Author)
The revision addresses the comments on the original version.

(Remarks on code availability)

Reviewer #4

(Remarks to the Author)
I co-reviewed this manuscript with one of the reviewers who provided the listed reports. This is part of the Nature Communications initiative to facilitate training in peer review and to provide appropriate recognition for Early Career Researchers who co-review manuscripts.

(Remarks on code availability)

Reviewer #6

(Remarks to the Author)
I co-reviewed this manuscript with one of the reviewers who provided the listed reports. This is part of the Nature Communications initiative to facilitate training in peer review and to provide appropriate recognition for Early Career Researchers who co-review manuscripts.

(Remarks on code availability)

Version 2:

Reviewer comments:

Reviewer #1

(Remarks to the Author)
I am satisfied that the authors have addressed my comments/concerns and recommend publication.

(Remarks on code availability)

Reviewer #2

(Remarks to the Author)

(Remarks on code availability)

Open Access This Peer Review File is licensed under a Creative Commons Attribution 4.0 International License, which permits use, sharing, adaptation, distribution and reproduction in any medium or format, as long as you give appropriate credit to the original author(s) and the source, provide a link to the Creative Commons license, and indicate if changes were made.
In cases where reviewers are anonymous, credit should be given to 'Anonymous Referee' and the source.
The images or other third party material in this Peer Review File are included in the article's Creative Commons license, unless indicated otherwise in a credit line to the material. If material is not included in the article's Creative Commons license and your intended use is not permitted by statutory regulation or exceeds the permitted use, you will need to obtain permission directly from the copyright holder.
To view a copy of this license, visit https://creativecommons.org/licenses/by/4.0/

Response to REVIEWER COMMENTS

We first would like to thank the reviewers for their constructive and supportive comments. We strongly believe their comments have helped us improve our manuscript.

Reviewer #1 (Remarks to the Author):

The manuscript from Claussen et al. develops a machine learning model to distinguish directly interacting protein–protein interactions (PPIs) from indirectly associated proteins. The model is trained on features derived from a variety of mass spectrometry (MS)–based experiments, including co-fractionation, proximity labeling, and large-scale AP–MS studies such as BioPlex. Labels for the training set were assigned using experimentally determined PDB structures of multi-protein assemblies.

Unfortunately, the overall strategy appears conceptually flawed. A binary classification is insufficient to describe the underlying biochemical reality of protein interactions. In the MS-based input datasets used here, transient but direct interactions are often less strongly correlated than stable, indirect associations. For example, in co-fractionation or AP–MS experiments, weak or transient direct interactions may produce lower correlation profiles or peptide counts than constitutive complex members that do not directly contact each other. Among the MS techniques considered, only crosslinking mass spectrometry (XL–MS) provides unambiguous evidence for direct PPIs, yet in this study, XL–MS data are used only for validation, not model training.

We agree that binary classification has its limitations but we politely disagree that our strategy is conceptually flawed. We acknowledge in our original discussion section that transient interactions are difficult to capture with current proteomics methods, and this continues to be an outstanding challenge in the field. We additionally acknowledge examples of highly stable complexes (e.g. 20S proteasome) which are rarely disrupted, limiting our ability to discern direct interactions (see 3rd paragraph of discussion). Despite these limitations, our model successfully discriminates between direct and indirect interactions as demonstrated by its superior performance on the leave-out test set of direct interactions from PDB structures (Figure 2). Moreover, the binary classification model provides a practical means to prioritize the 200 million possible interactions for AlphaFold modeling efforts.

We also agree with the reviewer that XL-MS data provides evidence for direct PPIs. Unfortunately, our experience with current large scale XL-MS datasets is that they require strong thresholds to control for false positives, thereby limiting the total number of direct protein pairs suitable for use in a training set. Further, XL-MS is biased towards highly abundant proteins, even more so than our method. For example, our network spans >10,000 proteins which is 4 times larger than crosslinking experiments. Efforts by others in the field are currently being undertaken to reanalyze high-throughput XL-MS datasets, which may make them more suitable for machine learning training in the future. Currently, however, we consider XL-MS to be best utilized for validation.

We have revised our discussion section to clarify these points and include a more thorough discussion on the topic.

This issue is illustrated clearly in Figure 3. The CCT/TRiC complex consists of eight subunits arranged in a double-donut structure. These 16 subunits form a constitutive assembly and are not observed as individual subunits or sub-complexes. Consequently, AP–MS using a single subunit as bait retrieves all subunits at high abundance, even though each subunit directly contacts only a few partners (e.g., CCT2 contacts CCT4, CCT5, and the second copy of CCT2). The DirectContacts2 (DC2) scores for CCT2–CCTx pairs are all >0.5, and in many cases, indirect pairs score higher than the direct ones (e.g., CCT2–CCT7: 0.941; CCT2–CCT4: 0.629). Figure 3 highlights only the eight highest-scoring pairs, which obscures this pattern. Moreover, the figure depicts a super-assembly of two constitutive complexes, prefoldin (PFDN) and CCT. The most informative evaluation would test whether the model predicts the inter-complex direct interactions (e.g., PFDN6–CCT4 and PFDN4–CCT4 in the corresponding PDB structure). Yet these have low DC2 scores (0.111 and 0.0105, respectively), suggesting that the model primarily distinguishes stable, constitutive associations from transient ones, rather than truly direct from indirect PPIs.

We thank the reviewer for prompting this point for clarification. Counter to intuition, biochemical isolation of complexes such as AP-MS or CF-MS do not consistently retrieve all subunits in a complex. This, consequently, is the signal our machine learning method identifies when classifying between direct and indirect interactions. We now provide an example of a CF-MS experiment that shows separation of subcomplexes of the CCT/Prefoldin complex (Supplementary Figure 2F-G). When examining these elution profiles, we see that positive examples CCT3-CCT6 and CCT2-CCT7 profiles exhibit very high correlation and cluster together (Supplementary Figure 2F) orange and yellow boxes, respectively). In addition, direct inspection of the raw data reveals that these pairs have unique profiles compared to the other subunits of the CCT complex, sharing distinct peaks (Supplementary Figure 2G), suggesting that they are isolated separately from the full complex (i.e., subcomplexes). We also see evidence for CCT4 and prefoldin interactions with these data. Although the DirectContacts2 scores for PFDN6-CCT4 and PFDN4-CCT4 interaction are low, as the reviewer notes, the underlying CF-MS data show signal, suggestive of an interaction. Specifically, we see that CCT4 is more correlated with the subunits of the prefoldin subcomplex compared to other CCT subunits, although admittedly not enough to make a confident prediction (Supplementary Figure 2F, magenta box). Finally, what is important to remember is that our method is an integration of thousands of mass spectrometry experiments and we have appropriately modeled the error of our network which outperforms other networks.

The proximity-labeling data for prefoldin (Figure S4A) also support this interpretation. While the authors highlight direct pairs such as PFDN3/2 and PFDN3/5, the same data show non-contacting subunits (e.g., PFDN6 and PFDN2) co-detected in multiple experiments, as well as instances where PFDN3 or PFDN2 appear without any partners. It is also unclear why PFDN4 is missing from this figure.

We thank the reviewer for the opportunity to clarify. It is important to remember that our approach using the weighted matrix model does not rely only on the results of individual experiments but rather takes a statistical approach by integrating many experiments together. The signal from the weighted matrix model applied to proximity labeling data is based on the hypergeometric test across many experiments to identify pairs of proteins that are present in the same experiments more often than random chance. The reviewer notes that two non-contacting subunits, PFDN6 and PFDN2, are co-detected in multiple experiments. While the two subunits are indeed found in the same experiments, importantly they are never found alone, suggesting that they are less likely to directly interact. Alternatively, PFDN3 and PFDN5 are seen in several experiments by themselves with respect to the complex, and therefore are more likely to be directly interacting. When these data are evaluated using the weighted matrix model, the -log(pval) of PFDN6 and PFDN2 for the proximity labeling dataset is 12.6 which is considerably lower than the two interacting pairs PFDN3 - PFDN2 (18.7) and PFDN5 - PFDN3 (25.4). This difference is highlighted in Supplementary Figure 5C. By training on direct and indirect labels from the PDB, our machine learning model integrates many features, including the weighted matrix model of proximity labeling to build a classifier to discriminate between direct and indirect interactions.

We thank the reviewer for pointing out the absence of PFDN4 from the figure. The weighted matrix model feature applied to the Youn et al. dataset with a PSM threshold > 4 was shown to be a top feature in our AUPRC analysis (Supplementary Figure 4). PFDN4 is absent because it does not pass the abundance threshold of > 4 PSMs for any of the described experiments. We have updated the figure legend for Supplementary Figure 5A to specify this.

The global benchmarking further reinforces this view. The authors' prior hu.MAP3 model—trained on the same MS-derived features and datasets to predict co-complex membership, regardless of directness, performs comparably to DC2. The Precision–Recall plots (Figure 2 and Supplemental Figure 2C) and AlphaFold predicted interface comparisons (Figure 4A and Supplemental Figure 2D) show only modest improvements for DC2 over hu.MAP3. This marginal gain likely reflects that the MS-based features encode interaction strength or stability, not physical directness.

We thank the reviewer for making this observation and the opportunity to clarify. As the reviewer notes, the hu.MAP3 model shows strong performance in discriminating between direct and indirect interactions even though the model is trained to identify co-complex interactions. We do however observe the PR curve of hu.MAP3.0 does poorly in the high-confidence region (Figure 2). The PR curve for hu.MAP3.0 does recover but not to the precision level of DirectContacts2. This can be more directly seen in the AUC calculation (Figure 2 inset) where DirectContacts2 outperforms all other models including hu.MAP3.0.

We also observe DirectContact2's superior performance when compared to AF2 modeling. The reviewer's comment spurred us to investigate whether the high-confidence predictions substantially overlap with the high-confidence predictions from other models. We now include an

UpSet plot (Supplementary Figure 3C) which shows limited overlap among the top 5,000 predictions for each individual model. This suggests that at high levels of confidence, the models are largely complementary to one another, where DirectContacts2 makes a different set of predictions from the other networks.

Supplementary Figure 2B does show some separation between direct and indirect test-pair score distributions, but the overlap remains substantial. The discriminative power appears too limited to inspire confidence in DC2 predictions for complexes of interest to most researchers.

We agree with the reviewer that there remains overlap between direct and indirect interaction scores in Supplementary Figure 2E (updated). However, systematically distinguishing direct vs indirect interactions represents a significantly more challenging problem than identifying co-complex interactions, and therefore complete separation of score distributions is not expected given current experimental data. Higher DirectContacts2 scores are empirically enriched for direct physical contacts in independent benchmarks, allowing interactions to be ranked according to confidence. Consequently users can select thresholds appropriate for their application, prioritizing high precision when confidence is critical or increasing recall when broader coverage is desired. We have revised the Results section (see 'DirectContacts2 Network Outperforms Previous Networks on Leave Out Set of Physically Contacting Protein Pairs') to clarify the choice and appropriate use of score thresholds.

Figure 5 evaluates DC2 using >5000 PPIs identified by XL–MS from the Huang lab. The results are presented as Z-scores relative to random protein pairs. However, this background consists of non-interacting proteins, not pairs from the same complex lacking direct contacts. As such, the separation between the two distributions does not truly test the ability to classify direct versus indirect PPIs. The same analysis applied to hu.MAP3 yields similar findings: DC2 scores are somewhat higher than huMAP3 scores, but the difference (e.g., 80 vs. 55 standard deviations from background) is not biologically meaningful given the inappropriate null model. A more informative analysis would compare to protein pairs selected from known protein-assemblies, but also it would be helpful to show a simple confusion matrix or contingency table comparing XL–MS–validated PPIs with DC2 predictions.

We thank the reviewer for making this suggestion. We now construct a random background only from pairs of proteins in the same complex (see updated Methods), and we compare each network's enrichment for cross-links over this updated background. These new results show a similar conclusion as before, that the DirectContact2 network is more enriched with cross-linked pairs than the hu.MAP3.0 network. We have revised our methods to describe this new procedure and include raw overlap values of both DirectContacts2 and hu.MAP3.0 with the crosslink dataset. We hope the updated analysis sufficiently addresses any statistical concerns and more explicitly demonstrates our network's superior enrichment for cross-linked protein pairs.

Overall, while a machine learning model capable of accurately predicting direct interactions would be a valuable advance, this work does not convincingly achieve that goal. The DC2

model appears to primarily reflect the stability or affinity of associations rather than physical contact per se. I also note that while the manuscript suggests that proteome-wide AlphaFold screening is computationally prohibitive, recent work indicates that large-scale pooled AlphaFold-based PPI prediction is indeed feasible (see bioRxiv 2025.07.01.662654v2).

We appreciate the reviewer's concern regarding the interpretation of DirectContacts2 predictions. As discussed above, large-scale proteomics measurements capture a mixture of biochemical properties, including interaction stability and affinity, which inherently shape the signals available for model training. Within these experimental constraints, however, DirectContacts2 consistently shows improved discrimination between direct and indirect interactions compared to existing large-scale resources, including our previous co-complex networks, our previous attempts at identifying direct interactions, and the large-scale yeast2hybrid dataset considered to be the most comprehensive direct network available. These results indicate that, despite the limitations of current data modalities, the model enriches for physical contact relationships beyond what can be achieved using individual datasets alone. While modeling additional biochemical properties such as stability and affinity would be valuable, this represents a complementary objective beyond the scope of the present study.

Regarding the large-scale pooled AlphaFold approach (Todor et al. BioRxiv 2025), this is an exciting and substantial advance in the field. Their approach was applied to the 113,050 pairwise PPIs in *Mycoplasma genitalium* which has a reduced genome with orders of magnitude fewer PPIs than the 200 million possible pairwise PPIs in the human proteome. Todor et al. estimates a reduction of total inference time by half using the pooled approach. A recent BioRxiv paper (Schmid et al. BioRxiv 2025) estimates the total computational cost of inferring structural models for all 200M human pairs would require ~50M graphics processing unit (GPU) hours. Using a pooled method may reduce this cost to ~25M GPU hours. This represents a substantial reduction but remains computationally prohibitive. Utilizing our DirectContacts2 network and other prioritization strategies is still necessary for efficient structural modeling of human PPIs. We have updated our discussion section to highlight this point.

In summary, the manuscript is clearly written and well-presented, but the model's conceptual basis and evaluation do not support the claim that it distinguishes direct from indirect PPIs. I would recommend major revision or rejection unless the authors can demonstrate improved benchmarking against known direct/indirect contacts that DC2 specifically captures direct physical interactions rather than general co-complex association strength.

We again thank the reviewer for their close reading of our manuscript and we hope we have satisfactorily addressed their concerns in our revisions.

Reviewer #2 (Remarks to the Author):

Summary
The authors present DirectContacts2, which is a machine learning model designed to distinguish direct from indirect protein interactions using features derived from mass

spectrometry-based proteomics data. The model's performance was evaluated against existing methods, demonstrating better precision and recall in predicting direct contacts. The authors further validated their predictions using structure prediction approaches and highlighted several structural modeling examples of disease-relevant protein complexes to illustrate the model's applicability.

Major comments:

1. There appears to be several issues with the setup of the framework. As noted in the main text, the training data consists of 2,177 positives and 100,707 negatives, resulting in a positive-to-negative ratio of 1:50. According to the methods section, the authors used accuracy as the evaluation function in the training process, which is not suitable for imbalance datasets and may fail to capture the model's actual performance in addressing the minority class. Furthermore, in the methods section, the authors mentioned that "the training set consisted of 2,177 positive labels from 595 complexes of which 1,472 had entries in our feature matrix and 100,707 negative labels of which 47,246 had entries in our feature matrix". This indicates that a substantial proportion of training data contains missing values. However, the manuscript does not clearly explain how these missing values were handled or how the model makes predictions for samples with incomplete features.

We thank the reviewer for this comment and for giving us the opportunity to clarify. First, we want to mention the difference between our test and training sets. Our test and training sets were created from a benchmark of direct and indirect interactions derived from structures of protein complexes in the PDB. Having a limited number of training examples, we supplemented the negative labels in our training set with pairs of proteins in separate complexes which improved the overall performance of the model. As the reviewer noted, this results in our training set being imbalanced. Importantly, however, the test set (i.e. leave-out evaluation set) is constructed in a way to be more accurate to biological reality, selecting negatives from only within a complex. Our final evaluation of the model is based on this test set using a precision recall framework which appropriately deals with any class imbalance. We have updated the text to clarify this topic and added a new table (Table 3) for clarity. In addition, we provide a precision recall plot of the test set supplemented with inter-complex negatives, which shows a similar trend of DirectContacts2 outperforming other models (Supplementary Figure 2B).

The training of the model, like the reviewer mentioned, is done using the "accuracy" evaluation function. We agree that, in theory, the accuracy function for training a model has limitations and a balanced accuracy would be more appropriate due to the imbalance in our training data. However, when we build a model empirically using the balanced accuracy function in the AutoGluon framework it generates an equivalent model (i.e. same model and weights) as the accuracy function. This is due to AutoGluon utilizing the evaluation function differently for different models. For the chosen LightGBM model AutoGluon treats accuracy and balanced accuracy the same. So while the accuracy and balanced accuracy model building produces several different base models, the final weighted ensemble model is the same in our case regardless of the evaluation function used. We do therefore think training a model with the balanced accuracy evaluation function is likely better in most cases; however, when

implemented under the Autogluon framework on this classification problem, it does not alter the model. We have updated the methods (see 'Machine learning framework to discriminate between direct and indirect interactions') to mention this point. Importantly though, our final evaluation is done using precision recall analysis which robustly handles imbalanced benchmarks.

Regarding missing values, there are two cases that require handling. First, an individual pair of proteins in our training set may have no entries in our feature matrix at all. In this case, the protein pair is ignored and not used for training. If a protein pair is present in our test benchmark but lacks data in our feature matrix, it is given a 0 confidence score +/- a small random number and still evaluated in our precision recall analysis. This allows for the proper calculation of recall estimates. The second case is if the protein pair has partial evidence in our feature matrix, for example, the pair may have AP-MS data but not CF-MS data. In this case, the protein pair is used and any features with missing values are represented as NaNs. AutoGluon's recommendation is to use raw data with missing values as input (i.e. NaNs) due to different models dealing with missing values differently (the selected model, LightGBM, has native support for NaN values). We have updated the methods (see 'Clustering Method to Create Benchmark Test and Training Dataset') to include additional details of how our model was constructed including missing values.

2. The methodological novelty of the DirectContacts2 framework appears limited. According to the methods section, it relies on the same features as the previous work hu.MAP3.0 and used the weighted matrix model established in the previous hu.MAP study. Although the authors showed that the new model achieved better precision-recall in predicting direct protein interactions, the authors may wish to add additional clarification about what specific improvements or innovations distinguish this framework from prior work.

We thank the reviewer for this comment and realized we glossed over the advances in our methodology in the original manuscript. The main advance in our methods was the development of a robust framework for the construction of the benchmark test and training sets, which was nontrivial in terms of how to properly construct. Data leakage is a major concern when constructing test and training sets for machine learning and has historically been an issue in the protein interaction prediction field[1,2]. The present problem requires the test and training sets to be disjoint at the pairwise level but also complex level. This is because the correlation structure of protein complexes in high throughput datasets is thought to carry over between test and training sets even if pairwise interactions or individual subunits are disjoint. Our previous work within the hu.MAP workflow deals with splitting test and training sets based on complexes but we found this original method was insufficient for constructing a direct interaction benchmark. This was largely due to the limited number of labels of direct and indirect interactions and therefore we were not able to train a general model with it. We therefore developed a new greedy algorithm that first hierarchically clusters complexes based on their pairwise Jaccard indices to find a seed cluster and then efficiently adds remaining overlapping complexes to the cluster. This ultimately limits the loss of labels that our original method suffered from. We have now included additional details to highlight the novelty and detail our algorithm with pseudocode

in the methods section (see ‘Clustering Method to Create Benchmark Test and Training Dataset’). While in this work we relied on portions of our previous machine learning workflow and feature matrix, we found constructing a proper benchmark was the most significant part of the success and generality of our model. Additionally, we uploaded our benchmark to huggingface and we expect it will be the de facto benchmark for this problem in the field moving forward. Finally, we add text to the discussion section to highlight this development.

3. In the “Integration of Proteomics Data Improves Discrimination Between Direct and Indirect Interactions” section, the authors state that the negative set consists of “100,707 known indirectly and non-interacting (i.e., non-co-complexing) pairs of proteins.” However, in the “DirectContacts2 Network Outperforms Previous Networks on Leave-Out Set of Physically Contacting Protein Pairs” section, the authors mention that they “excluded the negative set pairs from separate complexes to provide a rigorous assessment of our model.” These two statements appear somewhat inconsistent. While it is reasonable to evaluate model performance on distinguishing direct from indirect interactions within the same complex, including non-co-complexing pairs during training could make the classification task substantially easier and potentially introduce bias into the model. This point should be clarified.

We thank the reviewer for their careful reading of the manuscript and giving us the opportunity to clarify this point. We realize now this description of our model was lacking and have updated the text and added a new table (Table 3) for clarity. As mentioned above in response to point 1, we supplemented the training set with additional non-co-complexing negative pairs which resulted in a total of 100,707 training negatives pairs. Our aim was to supplement negative labels which would aid in the discrimination of our mass spectrometry based evidence features. We expect, during training, there would be negligible overall difference between features of a within complex negative and a non-co-complexing negative. To test for potential bias added into the model by this process, we temporarily supplemented our test set in the same way we supplemented the training set and evaluated our model using a precision recall curve (Supplementary Figure 2B). The precision recall analysis shown in Figure 2 is evaluated on the more rigorous test set of excluding negative set pairs from separate complexes. We see similar behaviour amongst the models regardless of the test set used, with DirectContacts2 performing the best in both regimes (compare Figure 2 and Supplementary Figure 2B).

4. In the “Integration of Proteomics Data Improves Discrimination Between Direct and Indirect Interactions” section, the authors report that they “selected a DirectContacts2 score threshold of ≥ 0.5,” based on the score distribution shown in Supplementary Figure 2A. However, the rationale for choosing this threshold is not clearly explained. Specifically, in Supplementary Figure 2A, the small “bump” corresponding to higher DirectContacts2 scores occurs around 0.95, making it unclear why 0.5 was chosen as the cutoff. The authors should provide a justification for this threshold selection (e.g., whether it was empirically optimized, based on prior studies, or derived from precision-recall tradeoffs).

The reviewer makes a valid point about confidence thresholds and cutoffs. Our original intention behind demarcating regions of differing confidence with hard numerical cutoffs (e.g. DC2 score

≥ 0.5, DC2 score ≥ 0.7) is to maximize its utility by providing interpretable labels. However, for many applications, we anticipate the network to be used as a ranked list allowing for lower thresholds such as DC2 score ≥ 0.5 (or lower) in cases when a higher recall is desired. This was our original justification for a threshold of 0.5 but as the reviewer pointed out we should choose a more theoretically justifiable threshold. We therefore recommend two thresholds, 0.9 and 0.7. First, we choose 0.9 because of the peak (or "bump") in the density plot in the updated Supplementary Figure 2D. In addition, for a higher recall network, we choose 0.7 because at this threshold we begin to see more positives than negatives (Supplementary Figure 2E). After filtering through known and predicted complexes, this results in 6,967 (≥ 0.9) and 16,461 (≥ 0.7) confident protein pairs. We also now provide a new Table 1, which evaluates several performance metrics at these thresholds and see superior performance over other networks. The full set of predictions is available for others to choose their own thresholds as their application warrants. We have now updated the text (see 'DirectContacts2 Network Outperforms Previous Networks on Leave Out Set of Physically Contacting Protein Pairs') to reflect these new thresholds.

5. In the "DirectContacts2 Guides the Structural Model of a Human Ciliopathy Complex" section, the authors highlight the OFD1-PIBF1-FOPNL(CEP20, FOR20)-KIAA0753 complex as an example to demonstrate how DirectContacts2 can guide AlphaFold modeling. However, this complex has already been identified in prior datasets (e.g., hu.MAP2.0), which diminishes the novelty of this example and does not convincingly illustrate the unique contribution of DirectContacts2 in structural modeling. The authors are encouraged to present novel direct interactions or complexes uniquely discovered by DirectContacts2, ideally supported by independent functional evidence or orthogonal validation.

Thank you for this comment. We agree that the novelty of the OFD1-KIAA0753-FOPNL example does not lie in the prediction of the complex but rather lies in the structural modeling based on our DirectContacts2 predictions. In particular the complex does not have a solved experimental structure nor has it previously been structurally modeled (to our knowledge). The DirectContacts2 prediction allowed us to confidently build structural models of the appropriate interactions and place these proteins in their three-dimensional arrangement. Moreover, the placement of human disease mutations into their three-dimensional context is extremely novel and suggests a molecular mechanism for this disease. We would also like to point out additional novel examples we highlighted in the manuscript that were predicted by our network including NEFM-NEFL, NEFM-VIM, HYOU1-HSPA5, and TMED10-TMED5, all of which have crosslinking support which bolsters their confidence. That being said, we welcome the suggestion to identify additional novel predictions by DirectContacts2. We therefore provide three additional examples of uncharacterized proteins associating with novel interactors (H2AX - H2BC17, C2orf74 - PPP1CA and C15orf61 - PCDHAC2) in Supplementary Figure 6.

6. One of the main advantages of DirectContacts2 over structure prediction-based approaches (e.g., AF3) is its ability to generate large-scale, all-by-all predictions of direct interactions across nearly the entire proteome. However, this strength is not sufficiently emphasized in the current manuscript. Much of the paper focuses on validating predicted

interactions, many of which correspond to previously known interactions. To better highlight the novelty and distinct contribution of DirectContacts2, it would be valuable to include additional analyses that demonstrate its innovation, for example, performing some network-scale functional analyses.

We thank the reviewer for encouraging further discussion of how DirectContacts2 can be used in system-wide analyses. We therefore asked if we could test the hypothesis that protein interactions evolve primarily with their complex or independently. We first compared the evolutionary age of high-confidence DirectContacts2 pairs to random pairs and showed DirectContacts2 pairs have more similar evolutionary ages (Figure 7A). We then tested if DirectContacts2 pairs have similar ages as their complexes suggesting they evolved together or if they have different ages suggesting they evolved separately in a piecemeal fashion. We see in Figure 7B, most directly contacting pairs have similar ages to their complexes yet there are some directly contacting pairs which do not. We highlight several of these examples as exceptions to the rule of complex evolution including INTS9 - INTS11 of the Integrator complex (Figure 7C), and RNF146 - CHD1L of the DNA Damage Response complex (Figure 7D).

Minor comments:

1. In Figure 1B, the “Benchmark” component (grey box) in the workflow diagram is somewhat confusing. The arrow connecting the “Benchmark” dataset (containing direct and indirect protein pairs) to the “Feature matrix” (grey-blue box) suggests that these data are used as input features. However, they should instead serve as ground truth labels. Additionally, please clarify how the number 5,252 in the “Benchmark” box was calculated. In the main text (line 129), it is stated that there are 2,177 known directly interacting pairs and 100,707 known indirectly and non-interacting (non-co-complexing) pairs, so the derivation of 5,252 is unclear.

We have altered our workflow (Figure 1B) to highlight the use of PDB structures as our test and training labels. We have also created a new table (Table 3) which details the positive and negative labels used in our study and have clarified the text.

2. In Figure 2, panels A and B appear redundant and could be combined into a single panel that shows both precision-recall curves and auPR values. The authors may consider adding ROC curves and auROC values for additional evaluation. Also, additional details are needed to clarify whether the methods for comparison were applied to the same set of protein pairs and how fair comparisons were guaranteed in the evaluation.

Thank you for the suggestion. We now add the bar plot as an inset in one panel with the curves. We have also added a ROC curve (Supplementary Figure 2A) and calculated several metrics including F1 score, MCC, and FDR for comparable sets from each network (Table 1).

In reference to comparison of models, yes, all the same sets were used for PR curve (and ROC) comparison. We have now clarified this in the methods (see ‘Calculation of Precision, Recall, F1, MCC, FDR, and ROC’).

3. In the “Integration of Proteomics Data Improves Discrimination Between Direct and Indirect Interactions” section, the authors mentioned that they “filtered our network by known co-complex associations in hu.MAP3.0, CORUM, and ComplexPortal”. It is unclear, however, how the resulting network can then be fairly compared to the hu.MAP 3.0 network, given that hu.MAP 3.0 itself was used as part of the filtering process.

We thank the reviewer for making this point as we see the description of this analysis may be unclear. We have now updated the precision-recall curves in Figure 2 to now show raw DirectContacts2 predictions (i.e. prior to filtering by complex). We additionally provide precision-recall curves for DirectContacts2 pre- and post-complex-filter over our leave-out test set, which directly compares the two and shows negligible change (Supplementary Figure 2C). We also clarify our rationale for filtering the network by known and predicted complexes, as this reduces promiscuous interactions like HLA and HSP, and we additionally see better enrichment for confident AlphaFold models (Supplementary Figure 3A).

4. In the section “Enrichment of High Confidence Multichain AlphaFold2 Models in DirectContact2 Predictions”, the text states that “we calculate the fraction of each network’s most confident set of predictions with confident AlphaFold models (pDockQ ≥ 0.23) for increasing sizes of top-ranked pairs.” However, in Figure 4, the y-axis label is “pDockQ > 0.23”, which is inconsistent and should be unified.

Thank you for pointing this out. We have updated our Figure 4’s axis labels to be consistent with the methodology outlined in the text.

5. In Figure 4A, the blue line representing “DirectContacts” is significantly shorter than the others. Does this indicate that “DirectContacts” predicts only ~2,500 pairs? Also, in the main text, please provide citations for all previously published methods used in benchmarking (e.g., DirectContacts, hu.MAP, etc.).

Yes, the original DirectContacts method predicts 2434 pairs but only 1478 overlap with the published AlphaFold pairwise datasets. We have now updated the text to be explicit on this point. We have also added / updated the citation for benchmarked networks into the main text.

6. In Figure 4B, it is unclear what each dot represents; please clarify this in the text. Moreover, many yellow dots have high DirectContacts2 scores but low fractions of AF3 models with high pDockQ scores, suggesting that the most confidently predicted direct interactions by DirectContacts2 are not supported by AF3 models. This observation appears to contradict the argument presented in this section.

Each dot in Figure 4B represents the top-N (x-axis) AlphaFold3 models according to their DirectContacts2 score. We have now clarified this in the text. The reason why low-N (leftward on the graph) have low fractions of models with pDockQ ≥ 0.23 is due to low sample numbers. For example, our highest confident DC2 pair we predicted in this set (MAGEB4, MAGEA1) had a score pDockQ < 0.23 but most other confident DC2 pairs were above 0.23, resulting in an

early recovery to a fraction of over 0.7 models. We now color the curve by the number of predictions evaluated at each point to aid the reader.

7. The legend next to Figure 5D is confusing. The blue and dark red edges represent “Satisfied Xlinks” or “Violated Xlinks” in the structure models from panels B/E/F, while the green edges indicate “Contacts supported by Xlinks” in the network shown in panel D. However, combining these legends next to panel D makes interpretation unclear. Consider separating or relabeling them for clarity.

We agree with the reviewer that the current legend layout is confusing. We have revised Figure 5B-F to include dedicated legends per panel to improve clarity. We hope this layout is more interpretable.

8. In “DirectContacts2 guides the structural model of a human ciliopathy complex” section line 325, please explain the sources of the multiple OFD1 mutations and further provide the detailed information for these selected mutations (i.e., associations with any diseases; deleteriousness scores from variant effect prediction, etc.). It can make the statement “the disruption of this interface is responsible for the downstream functional defect giving rise to a clinical phenotype” stronger and convincing.

Thank you for this comment. The mutations in OFD1 are all from human patients with Orofaciodigital syndrome 1, a known ciliopathy disease that gives rise to serious clinical phenotypes in females (e.g. malformations in the face, oral cavity, and digits) and neonatal lethality in males. We have made updates to the text to highlight these mutations.

9. In Supplementary Figure 3, the y-axis tick labels are confusing. They appear to correspond to the feature names used in the model, but the current labels are difficult to interpret. Please rename them to improve readability and interpretability.

We agree that the feature names can be slightly cryptic and to address this we have added Supplementary Table 11 that provides additional description for each feature in our entire feature matrix, including the publication for the data from which the feature is derived, experimental conditions, cell types used, and the metric used to generate that feature. We believe that this curation will be a useful resource for others in the community and hope that it will ultimately make using our feature matrix more accessible. We also update labels on Supplementary Figure 4 (updated) for clarity. We thank the reviewer for the comment and prompting this response.

10. In Supplementary Figure 4, the colors assigned to Prefoldin subunits in panel A do not match those in panel B. Please either relabel the subunits in panel B or adjust the color scheme so that they correspond consistently across panels.

We have updated the colors of PFDN3 and VBP1 in panel A of the now Supplementary Figure 5 (updated) to better correspond to the subunit color shown in panel B.

11. In “Precision Recall Analysis” of the Methods section, please use the full name of “sklearn” package (should be scikit-learn).

We have updated the methods section to reflect the complete name of the package.

Reviewer #3 (Remarks to the Author):

The authors present a machine-learning model for separating direct from indirect protein–protein interactions. This is an important distinction that is often ignored in constructing molecular network models based on proteomics data. The new model was trained on large-scale mass spectrometry datasets. The authors report that their model can separate direct from indirect protein–protein interactions more effectively than the existing methods. Thus, the method should be of interest to the scientific community, including systems biologists and structural biologists. I have the following comments/questions for the authors:

1. The model is trained on features derived from mass spectrometry datasets and supervised using only the protein–protein interactions documented in the PDB. The model is then applied to the full set of ~25M human protein pairs. Because the model is trained on stable, crystallizable complexes, how applicable is it to identifying novel physical interactions that are transient, weak, and condition dependent? In other words, how much can DirectContacts2 generalize beyond the training set in the PDB?

This is an excellent point and we thank the reviewer for making it. In our discussion we mention, DirectContacts2 has limitations identifying transient and weak interactions. This stems from the majority of available mass spec data, and consequently the majority data type in our training set being AP-MS and CF-MS, which are limited in their ability to capture these interactions. Experimental techniques like cross-linking mass spectroscopy or proximity labeling are capable of capturing weak or transient interactions. Here we reanalyze and incorporate proximity labeling data while we use cross-linking data for validation. This suggests there are data in our model which are within the distribution of weak and transient interactions but still limited.

Other interactions not present in the PDB involve proteins with intrinsic disorder. In Supplementary Figure 3D we compare AlphaFold model confidence to disorder content for high confident DirectContacts2 predictions (>= 0.9 confidence score). We confidently identify several pairs of proteins to directly interact that have substantial disorder (e.g. spliceosome subunits SNRPB (72% disorder) and SNRNP70 (66% disorder) and SOSS complex subunits NABP2 (58% disorder) and INIP (60% disorder)). These cases show that, even though we trained on labels from the PDB, we are still able to find interactions that may not be found in the PDB.

2. The DirectContacts2 score distribution is bimodal, with a large peak near 0 (presumably false interactions) and a small peak near 1 (likely true interactions) (Supplementary Figure 2A). Authors have used 0.5 as the confidence cutoff, but this appears to have been

an ad hoc choice. Is there a rationale supporting this choice? The authors should probably provide a threshold sensitivity analysis, reporting precision, recall, F1, MCC, and/or FDR at each possible cutoff. Without such an analysis, it is unclear if 0.5 is optimal in some sense; for example, one could argue based on the bimodal distribution that a higher threshold (eg, 0.7–0.9) might yield "better" predictions.

We thank the reviewer for this comment. We responded to a similar critique above from reviewer #2 (major point 4). Briefly, we have updated our thresholds to 0.7 and 0.9 and provide justifications for these thresholds. Additionally we provide a table of performance metrics for each threshold and other models in Table 1 which shows these thresholds outperform other networks.

3. The AlphaFold validation shows that only about 50% of the highest-scoring predictions (DirectContacts2 > ~0.99) result in good structural models (pDockQ ≥ 0.23). This finding suggests that many predicted direct interactions, even with scores as high as 0.9, cannot currently be structurally modelled. It would be interesting to discuss such outcomes in terms of different kinds of structural modeling errors, intrinsically disordered regions, and other reasons.

We agree that this is an important point as there are several reasons why biologically relevant protein interactions are difficult to structurally model. As the reviewer suggested, inference algorithms like AlphaFold3 may fail due to modeling errors of a variety of types including low sequence coverage resulting in shallow MSAs and disordered regions. This points to an advantage of our method which is based solely on mass spectrometry experiments and does not suffer from these problems. We investigated the degree of which our high confidence DirectContacts2 predictions produced confident structural models given their disorder content. As expected, in Supplementary Figure 3D, we do see a slight negative correlation between percent disorder of proteins in a direct interaction and pDockQ score of AlphaFold3 models (-0.215 Pearson correlation coefficient). We now state this in our Results section "Enrichment of High Confidence Multichain AlphaFold2 Models in DirectContact2 Predictions" along with several relevant examples of confident DirectContacts2 predictions with high disorder content.

Reviewer #4 (Remarks to the Author):

I co-reviewed this manuscript with one of the reviewers who provided the listed reports. This is part of the Nature Communications initiative to facilitate training in peer review and to provide appropriate recognition for Early Career Researchers who co-review manuscripts.

Reviewer #5 (Remarks to the Author):

I co-reviewed this manuscript with one of the reviewers who provided the listed reports. This is part of the Nature Communications initiative to facilitate training in peer review and to provide appropriate recognition for Early Career Researchers who co-review manuscripts.

REVIEWER COMMENTS

Reviewer #1 (Remarks to the Author):

The manuscript revisions clarify and improve several technical aspects of the methodology that were raised by the reviewers, including the choice of confidence thresholds, balance between training and testing sets, the null model used for the CLMS benchmarking, and the description of the benchmarking procedures. These changes improve the clarity of the manuscript and make the evaluation of the method more transparent.

My primary concern remains that the experimental readouts used as input features are derived from MS-based proteomics experiments that do not clearly distinguish between interaction strength/stability and physical interaction directness. While the authors provide arguments that integrating many experiments can help extract such signals, I remain cautious about the interpretation that the model specifically captures direct physical contacts. For example, Figure 4A shows only modest improvement in recovering high-quality AF3 structural models relative to hu.MAP3, which is trained on essentially the same underlying data but without labels explicitly intended to represent direct contacts.

Thank you for this comment because it is an important one. First, it is important to state that none of the mass spectrometry experiments utilized here in our model were originally meant for identifying direct physical interactions but rather co-complex interactions. Yet, when we train a model using these data and based on labels of direct physical interactions from the PDB we identify proteins which directly interact. Crucially, this is a statistical argument that is quantified by a highly error-controlled confidence score where the higher the score, the more likely the prediction is true. The other important thing to note is that our model was applied to 25 million pairs of proteins which far exceeds other high-throughput direct physical interaction methods. This is due to the extremely large size of our mass spectrometry experimental dataset which samples different cell types, tissues, and parts of the proteome.

As the reviewer states, our previously published co-complex model hu.MAP3.0 shows relatively good performance on recovering high-quality AF3 models (Figure 4A). Interestingly, however, the hu.MAP3.0 network suffers on the leave-out test benchmark evaluated using precision recall analysis showing a large dip in precision with high-confidence predictions (Figure 2). Our DirectContacts2 model, presented here, not only performs well on all independent benchmarks we compare, it outperforms all other models.

A similar pattern is seen in the CLMS benchmarking analysis. According to the revised methods section, 229 crosslinked protein pairs exist within the “universe” of interactions shared among the three datasets considered. DirectContacts2 identifies 127 of these pairs among its 1,418 predictions, while hu.MAP3 identifies 123 among 2,239 predictions. While DirectContacts2 shows somewhat stronger enrichment for crosslinked pairs than hu.MAP3, both methods still fail to recover a substantial fraction of the experimentally observed direct interactions (eg, the 229 crosslinked PPIs).

Thank you for giving us the opportunity to address this point. It is important to remember that while high-throughput XL-MS provides valuable evidence of physical contact, such datasets represent a sparse and biased sampling of direct interactions rather than a comprehensive ground truth. Crosslink detection is often limited by crosslinking efficiency, strong constraints on peptide detectability following proteolysis and MS acquisition, and residue-specific and geometric requirements that render many bona fide interfaces unobservable with a given crosslinking chemistry. These factors all lead to non-negligible false negative and false positive rates, even among stable complexes.

As a result, the set of 229 crosslinked PPIs should not be interpreted as a gold standard list of direct interactions present in the system. Therefore, failure to recover all of these pairs does not necessarily reflect on the model's inaccuracy, but rather the combined sparsity of XL-MS observations and differences in what each method is able to capture. The crosslinking dataset does, however, give an independent comparison for evaluating different prediction models, by which DirectContacts2 performs significantly better.

Minor, side point: it's not clear how they come up with the "universe" of 12,086 possible protein-pairs.

We apologize for the lack of clarity in our methods section, and we have updated it to address this point. In short, the set, or "statistical universe", of possible protein pairs was constructed by first determining the set of 1,329 proteins common between the three datasets (i.e. DirectContacts2, Hu.MAP3.0, Wheat et al.) to account for the models' compositional differences in coverage. From this, we generate an expanded set of all 882,546 possible pairs (1,329 choose 2). We further restrict this expanded set to the pairs of proteins known or strongly predicted to co-complex per literature-curated databases (i.e. CORUM, ComplexPortal, Hu.MAP3.0), yielding the final 'universe' of 12,086 possible *in-complex* protein pairs which we utilize as a null background against which to compare X-link enrichment. We have clarified this methodology further in the main text.

That said, the authors make reasonable points regarding the ability of machine learning models to extract signal across many experiments even when individual datasets are noisy or incomplete. The revised manuscript is also technically clearer and better contextualized.

Overall, I remain somewhat skeptical that a machine learning model trained primarily on MS-based association data can fully resolve interaction directness. However, the approach appears useful as a prioritization or ranking framework for candidate direct interactions, and the resulting resource is likely to be of interest to the community, particularly if the manuscript frames the method as enriching for likely direct interactions rather than definitively detecting them.

We thank the reviewer once again and we hope we have alleviated any remaining concerns.

Reviewer #2 (Remarks to the Author):

Summary:
This revised manuscript presents DirectContacts2, a machine learning framework that aims to distinguish direct from indirect protein-protein interactions using features derived from large-scale mass spectrometry datasets. Although the authors have clarified several points from the initial review, including benchmark construction, handling of missing values, and negative sampling rationale, the central methodological concerns remain unresolved, limiting confidence in the validity and significance of the reported findings.

Follow-up comments:
1. The main concern about novelty remains. DirectContacts2 appears to use essentially the same overall feature space as hu.MAP3.0 and related previous work. The main difference is the benchmark construction and the relabeling of the task from co-complex association to direct-versus-indirect interaction. However, both this extension and the results still seem incremental. Even though DirectContacts2 is trained specifically on direct-versus-indirect labels, the gains over hu.MAP2.0/3.0 are modest. In Table 1, the performance improvement is limited. In Fig. 4A and Fig. S3B for the comparison of enrichment for confident pair interfaces, especially for the top-ranked pairs that are often the most relevant in practice, hu.MAP3.0 is not clearly worse and in some regions looks comparable. This makes the practical advantage of the new framework less convincing. To summarize, the work still appears to be a modest reworking of the previous hu.MAP framework, without the level of methodological innovation or validation expected for a high-impact publication. Comparison only to earlier hu.MAP networks is not enough. If the authors wish to argue that DirectContacts2 represents a meaningful advance, it should be benchmarked not only against prior co-complex networks, but also against current ML- and structure-based PPI prediction methods such as AF2Complex, RoseTTAFold2-PPI, Boltz-2, TT3D, and other relevant recent baselines. Without these comparisons, the manuscript does not convincingly establish either the novelty or the practical advantage of the proposed method.

We thank the reviewer for raising this concern. We would like to gently push back on the statement that our work is incremental. As the reviewer mentions, a major methodological difference between our hu.MAP3.0 model and our DirectContacts2 model is the benchmark test and training set. This difference should not be considered minimal or incremental as it required substantial development, including PDB-derived direct and indirect labels, novel clustering, stringent filtering, and train/test splits designed to avoid overlap of complexes and protein pairs, resulting in a benchmark that is more rigorous than methods that use randomly selected pairs for their negative set or co-complex pairs for their positive set. For example, several of the ML sequence based models utilize the STRING database as a way to train (e.g. TT3D) or test (e.g. RoseTTAFold2-PPI) their models. The STRING database, however, is limited as a benchmark for the problem of identifying direct physical interactions because STRING's physical interaction network is a mixture of direct physical interactions and co-complex interactions, ultimately confounding these two types of interactions. This again points to the value of our benchmark as an advance in the field, which is based solely on the PDB with appropriate splits into test and training.

To address the reviewer's suggestion to benchmark our DirectContacts2 network against other ML sequence-based PPI prediction methods, we first note that all methods suggested by the reviewer are complementary to the DirectContacts2 network because their underlying data is orthogonal. We now evaluate the relative performances of DirectContacts2 and RoseTTAFold2-PPI, as it was previously shown to have excellent performance in identifying direct interactions, has a publicly accessible set of predictions on human protein pairs, and has a benchmark of positive and negative protein pairs on which to test. Other suggested methods lacked either a sizable accessible set of predictions or benchmark suitable for fair comparison (e.g. AF2Complex has only been applied to *E.coli*, Boltz-2 has only been applied to only a minimal set of human protein interactions (1,394 pairs, Ille et al. BioRxiv 2025), TT3D was trained on human data, specifically the STRING physical network, but only applied to other model organisms). We evaluated RoseTTAFold2-PPI using their benchmark of 3 different classes of directly interacting proteins (i.e. Strong, Medium, and Weak, Data S2 in their publication Zhang et al. Science 2025). This was for several reasons: 1) the benchmark is derived from human PPIs in the PDB providing a set of positively labeled direct interactions and randomly selected negative interactions, 2) we could remove our training data from their benchmark to make a fair comparison, 3) the construction of the benchmark is independent of ours. We were not able to compare using our benchmark due to the limited accessibility of RoseTTAFold2-PPI's training set. This comparison is provided in Figure2B. We see that DirectContacts2 and RoseTTAFold2-PPI have similar performance on the strong interactors while DirectContacts2 outperforms RoseTTAFold2-PPI on the medium and weak interactors, suggesting DirectContacts2 identifies interactions on a wider variety of interaction types

Additionally, we measured the overlap between the DirectContacts2 confident network and RoseTTAFold2-PPI confident network (Supplementary Figure 2G). We show ~3,500 interactions are predicted by both networks while ~13,000 and ~25,800 predicted independently by DirectContacts2 and RoseTTAFold2-PPI, respectively. This limited overlap supports the view that the approaches are complementary rather than redundant: DirectContacts2 is based solely on high-throughput proteomics evidence, whereas RoseTTAFold2-PPI is sequence-based. The RoseTTAFold2-PPI study also identifies factors that limit sequence-based prediction, including MSA depth, selective pressure and conservation at the interface, number of interface residues, and interface residue variability. None of these limitations directly apply to DirectContacts2, again indicating the complementarity of the two approaches. Thus, predictions from one method may provide independent support for borderline predictions from the other.

Finally, while we agree that hu.MAP3.0 is competitive in some AlphaFold enrichment analyses, the broader set of benchmarks supports a practical advance. DirectContacts2 outperforms hu.MAP2.0, hu.MAP3.0, DirectContacts1, and HuRI on the held-out PDB-derived direct-versus-indirect benchmark. The HuRI network is a particularly relevant comparator, as it represents the largest yeast2hybrid screen available for humans, specifically assaying direct physical interactions. Interestingly, we also see that while hu.MAP3.0 incorporates substantially more mass-spectrometry data than hu.MAP2.0, it does not improve on the direct-versus-indirect benchmark (Figure 2A). This further supports our conclusion that the gain comes from

redefining the task and benchmark around direct physical contacts, rather than simply from adding more training data. We have revised the manuscript to make this distinction clearer and to include the new RoseTTAFold2-PPI comparison.

2. The clarifications regarding the “greedy clustering” and training objectives serve more to highlight the inherent limitations of the study than to mitigate them. The core technical contribution appears to be an incremental data-splitting strategy, essentially a standard search for connected components, which the authors overstate as a novel “greedy clustering” algorithm. The authors didn’t explain why other deduplication tools (e.g., CD-HIT, MMseqs2-based splits) are insufficient for this specific problem. In addition, this approach, combined with a highly limited test set of only 50 complexes and a training phase reliant on “easy” inter-complex negatives, raises serious doubts about the model’s ability to generalize to the broader human proteome. The provided pseudocode is not clear and contains several inconsistencies and apparent logical errors (e.g., incorrect stopping condition, ambiguous indexing of clustering outputs, and improper list operations). As written, it is difficult to reproduce the method, which undermines the transparency and reliability of the benchmark construction.

We thank the reviewer again for their close reading of the manuscript and appreciate the opportunity to clarify several points of possible confusion. While we did develop an implementation of a clustering algorithm, the reviewer is correct that there are other clustering methods that could achieve similar results. What is new however is the application of a clustering algorithm to the generation of the benchmark. This improves on the common approach of randomly splitting the benchmark which results in loss of samples after redundancy is eliminated. In addition our benchmark improves on previous published work such as those that use the STRING database which confounds direct physical interactions with co-complex interactions.

In regards to the sequence clustering tools the reviewer mentions, these tools are not applicable as our goal is to remove redundancy of protein pairs between the test and training sets, not among protein sequences. Since all of our features are mass spectrometry based, each protein is independent (i.e. only unique peptides are utilized for identification) and no feature is based on a single protein but rather all features describe pairs of proteins. Therefore, it is not necessary to cluster proteins based on sequence. Again, the solution we develop here eliminates redundancy of protein pairs across test and training splits with limited or no loss of pairs.

We disagree with the reviewer that the problem of identifying direct physical interactions is “easy” when the negative set is randomly selected from non-co-complexing pairs. Many state-of-the-art ML based methods such as RoseTTAFold2-PPI use randomly selected pairs for the negative sets of their evaluation benchmarks and do not have greater than 0.5 area under the precision recall curve. This suggests considerable more work is left to be done to ultimately solve these problems.

With respect to the reviewer's comment that our benchmark is limited, we have now evaluated our method to the benchmark created to evaluate RoseTTAFold2-PPI which was independently constructed from ours (see point 1 above, Figure 2B).

We thank the reviewer for the close reading of our clustering method and apologize for the lack of clarity in our pseudocode. We have updated the pseudocode and also added a link to our github repository where an implementation can be found.

3. The authors' response regarding accuracy versus balanced accuracy relies on an unverified, framework-specific behavioral claim rather than principled reasoning. Throughout the manuscript, critical methodological decisions including model selection, training objective, missing value handling, and ensemble construction are justified primarily by deferring to the AutoGluon framework rather than by principled experimental design. Furthermore, in the revised manuscript and the response letter, the observation that "accuracy" and "balanced accuracy" yield identical models appears to be a consequence of the AutoGluon framework rather than a principled property of the learning problem. The authors seem to be unclear about the training objectives and evaluation metric in the model selection process. In other words, the training objective is not directly tied to the evaluation metric, and the role of the metric is primarily in model selection. The training objective for each binary classifier doesn't seem to adjust the class imbalance according to the AutoGluon implementation. Therefore, this observation does not justify the use of accuracy in an imbalanced setting, nor does it demonstrate that class imbalance has been appropriately handled. Instead, it raises additional concern about the validity and correctness of the entire model training and evaluations. To summarize, the use of accuracy as the evaluation metric in a highly imbalanced setting is not adequately justified, and the observation that accuracy and balanced accuracy yield identical models appears to reflect framework-specific behavior rather than a principled result. Furthermore, the feature selection process is insufficiently described, and no explicit strategies are employed to address class imbalance.

We thank the reviewer for their comment and the opportunity to clarify this point. We agree that class imbalance is an important consideration for model training and evaluation. To directly test whether mitigation of the class imbalance in our training dataset would improve model performance, we trained LightGBM models using the native LightGBM Python package under various weighting strategies, including an unweighted model, where both negatives and positives receive equal weight, as well as models that increased the relative weight of positive examples. We then evaluated these models on the leave-out test and compared their performance to our DirectContacts2 model.

As shown in the figure included below in this response, the best performing LightGBM model is the unweighted model, whereas increasing the weight of the positive examples substantially reduces the performance. Although we agree that class imbalance should be considered carefully, our results indicate that explicit mitigation through class reweighting is not beneficial for this prediction task.

Importantly, our final model evaluation is performed using precision-recall analysis, which robustly handles class imbalance by focusing on recovery of the positive class. Using this analysis, we see that DirectContacts2 remains the top-performing method on both a balanced (Fig. 2A) and unbalanced (Supplementary Figure 2B) leave-out test set. Together these results suggest that our original DirectContacts2 model is robust to the class imbalance in the training data and that additional class reweighting strategies do not improve performance.

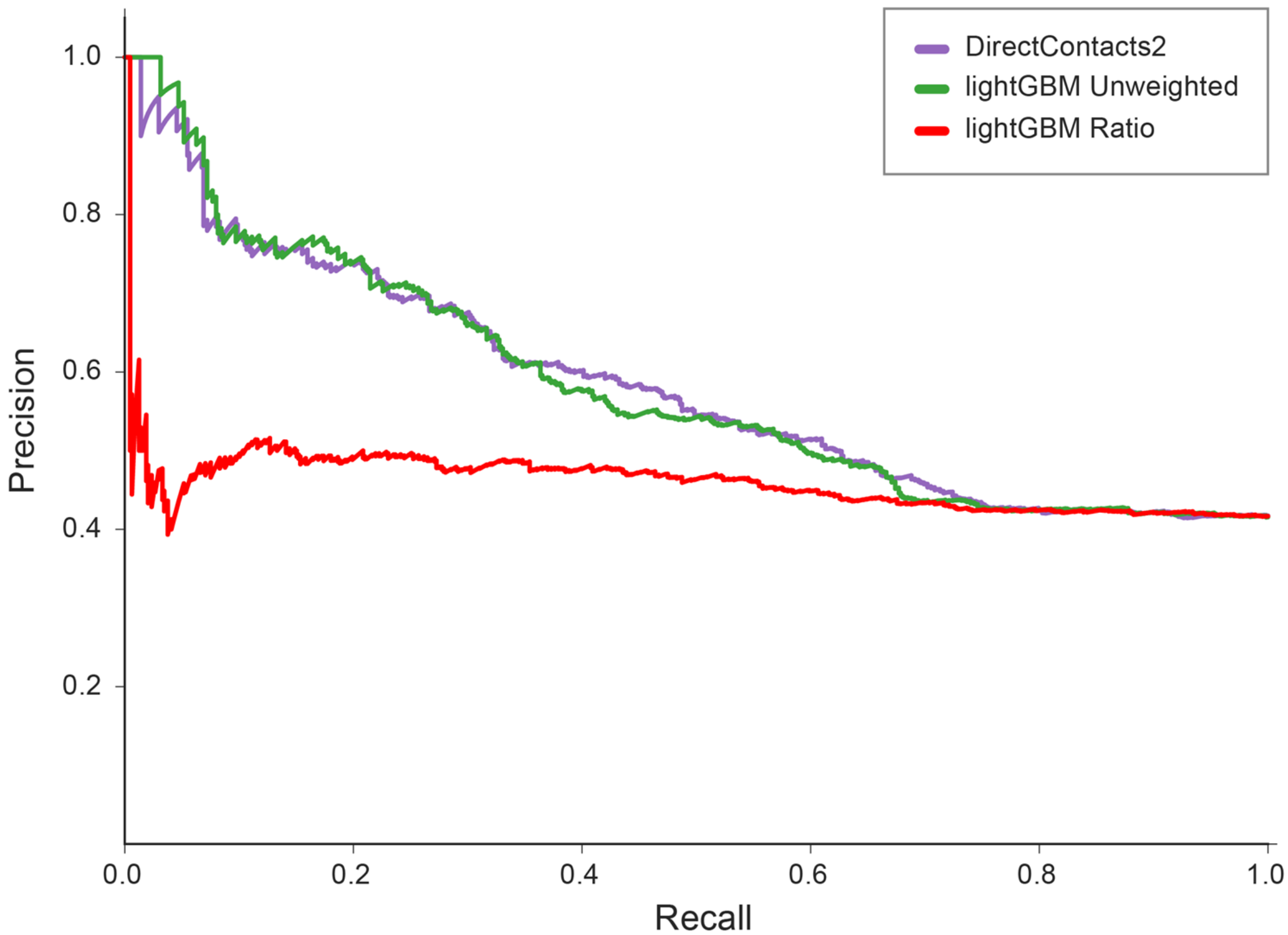

Precision-Recall analysis of DirectContact2 vs lightGBM models.
All lightGBM models were trained using the same cross-fold validation set and set of typical base parameters. The lightGBM models only differ in their weighting of positive examples during training, including: a weight of 1 (Unweighted) and the number of negative examples divided by the positive examples (Ratio). The comparison is done on the intra-negative test set (Supplemental Table 4).

4. In addition to the class imbalance issue, the internal cross-validation setting also seems to be problematic. There’s no enforced complex-level separation during the cross-validation step, therefore data leakage may be reintroduced at the feature and model selection stage, undermining the carefully constructed outer benchmark split. The model is subsequently applied to tens of millions of protein pairs without validation of generalization or calibration, and the final network is heavily filtered using existing complex databases, limiting the extent to which the

results represent de novo predictions. Overall, the methodological rigor of the modeling pipeline falls short of the standards expected for a high-impact study.

We agree with the reviewer that there is no enforcement of complex-level separation during the training step but we disagree that this undermines or limits our methodological rigor. All model features are defined at the protein-pair level, and pairs only appear once in the training set, meaning no explicit feature leakage can occur between the training and validation splits during a cross-validation. The ultimate test of our network is the leave-out benchmark which has no overlap with the training set at the pairwise or complex level. In addition, we now evaluate using an independently developed benchmark (see point 1 above). Moreover, separating leave-out test and training sets at the complex-level exceeds the standards of the field as many state of the art methods do not do this.

5. The description of the final classifier is also somewhat misleading. The manuscript states that AutoGluon trained 13 base models and then a WeightedEnsemble_L2 model using these base models. However, the authors also state that only the LightGBM base model received non-zero weight in the final WeightedEnsemble_L2. If so, the final predictor is effectively just the LightGBM model rather than a meaningful ensemble. While it is fine to note that an ensemble-selection procedure was attempted, the manuscript should describe the final deployed classifier more directly and avoid implying that predictions benefited from combining multiple base learners when the ensemble in practice collapsed to a single model.

We again want to thank the reviewer for their careful reading of the manuscript and providing us the opportunity to clarify this point. The reviewer's conclusion is correct that the final DirectContacts2 model is a LightGBM model. We have edited the language in the main text (see section: "Integration of Proteomics Data Improves Discrimination Between Direct and Indirect Interactions") to make this more clear.

6. We appreciate the authors for providing Table 3, but it also shows that the number of pairs actually used for model fitting is much smaller than the benchmark counts emphasized in the text, which is misleading. For example, the main text described the training set as having 2,177 positives and 100,707 negatives, but only 1,472 positives and 47,246 negatives appear to have feature-matrix entries and are actually used for training. The same issue applies to the test set. Pairs without feature-matrix entries are excluded from the model and should not be presented interchangeably with the effective training and test samples. The manuscript should explicitly report the number of PPIs with usable features that were actually used for training and evaluation, rather than foregrounding the larger benchmark totals in a way that overstates the amount of informative data available to the model.

We thank the reviewer for their comment and agree that this could be more clear. We have edited the figure legend of Figure 1 to include descriptions of the entries that are used for model training and evaluation and edited the main text (see "Integration of Proteomics Data Improves Discrimination Between Direct and Indirect Interactions"). We retain the description of the entire feature matrix and provide the unfiltered benchmark in Supplementary Tables 1&2 and on our

huggingface repo. We strongly believe that the careful curation of this benchmark will be useful to other researchers and that it will become the new standard for evaluating direct protein-protein interaction prediction methods.

7. The explanation for adding non-co-complexing pairs to the training set is understandable, given the limited number of within-complex indirect negatives. However, this still remains a concern. The biological question of interest is whether the model can distinguish direct from indirect interactions within complexes. Once a large number of clearly unrelated inter-complex pairs are added as negatives, the training problem becomes easier and shifts toward a broader distinction between associated and unrelated pairs. Even if the evaluation is later restricted to within-complex negatives, the training objective is no longer fully aligned with the biological claim.

The reviewer notes a valid concern about inter-complex negatives changing the empirical distribution. The central biological question is the discrimination of direct from indirect interactions among proteins that participate in the same complex, and we do not view inter-complex negatives as a substitute for the within-complex problem. However, we do not believe that their inclusion makes the training objective misaligned with the biological claim. Instead, it places the direct-versus-indirect problem within a broader and biologically appropriate space of possible protein pairs over which the model must learn calibrated, low-confidence predictions. The available structurally resolved human complexes provide too few within-complex indirect examples to train a high-dimensional model using only direct and indirect co-complexing pairs without substantial risk of overfitting to a sparse negative set. Consistent with this concern, during model development we found that models trained exclusively on indirect co-complexing negatives were less effective when evaluated on our within-complex benchmark, and performance improved when the negative set was expanded to include pairs from separate complexes. We have clarified this rationale explicitly in our main text.

We believe this improvement arises because inter-complex negatives help define regions of feature space that should receive low direct-interaction scores, thereby regularizing the model and reducing high-confidence predictions for unrelated protein pairs. The within-complex indirect pairs remain the critical negatives that determine the biologically relevant decision boundary, as defined by our benchmark, while the inter-complex pairs serve to stabilize representation during training and improve model calibration across the broader PPI space. The use of non-associated or random protein pairs as negative examples is also common practice for large-scale PPI prediction, including recent structure-based models such as RoseTTAFold2-PPI. Importantly, our evaluation is stricter than this training setup, as the leave-out benchmark excludes protein pairs and complexes overlapping with training, and its primary negatives are within-complex indirect pairs rather than inter-complex unrelated pairs. Thus, any gain from inter-complex negatives cannot by itself explain the reported performance unless the model also succeeds on the within-complex discrimination task. DirectContacts2 outperforms the tested alternative networks on held-out within-complex direct and indirect pairs by precision-recall and ROC analyses (Figure 2, Supplemental Figure 2A, 2B), indicating that the broader negative

training space improves calibration and regularization without reducing the model to an associated-versus-unrelated classifier.

8. In Fig. S2E, the overlap between the score distributions for direct and indirect pairs is still substantial. Since these pairs come from well-characterized PDB complexes, this is not a minor issue. If the model does not separate the two classes well even in this benchmark setting, it is difficult to be confident about performance on less well-supported pairs. This does not mean the model has no value, but it suggests that the paper is showing enrichment rather than strong discrimination. That is a more limited claim than the manuscript currently makes.

We thank the reviewer for their comment and the opportunity to clarify this point. We fully acknowledge that the score distributions for direct and indirect pairs are not completely separated. However, we would like to politely push back that this limits the use of our model or indicates that DirectContacts2 is only capturing broad enrichment.

Although our benchmark is derived from well-characterized PDB structures, the classification problem is intentionally difficult. Our negative examples are not composed of random protein pairs, as is commonly seen in other large-scale screens for protein-protein interactions (see above point 7). Instead, the benchmark includes difficult indirect examples, including within-complex indirect interactions, which can have proteomics-derived association signals and in some cases we fail to distinguish as noted in our discussion. Therefore, the benchmark asks whether the model can distinguish between direct physical interactions from closely related indirect associations rather than separating direct interactions from obviously unrelated proteins. This is a stringent and non-trivial distinction.

Although the score distributions are not completely separated, we rigorously evaluate the performance of our model in several ways. We test performance on held-out benchmark sets with no overlapping complexes, protein pairs, or proteins between training and testing (Figure 2A, Supplementary Figure 2A-B). We also compare DirectContacts2 against other state-of-the-art tools such as RoseTTaFold-PPI (Figure 2B). We validate our model's predictions by showing their enrichment in high-confidence AlphaFold models (Figure 4, Supplementary Figure 3A, B) and by using orthogonal cross-linking mass spectrometry data (Figure 5A). Across these analyses we show that DirectContacts2 consistently outperforms all other methods. We also note that across the ~25 million protein pairs scored by DirectContacts2 most pairs receive low scores, consistent with the expectation that most protein pairs do not directly interact (Supplementary Figure 2D).

These comparisons highlight the broader utility of DirectContacts2. The model can prioritize direct interactions when structures are unavailable and provide orthogonal evidence for structurally characterized interactions identified under non-native conditions.

9. The manuscript continues to emphasize large-scale applicability, but there is still not enough guidance on what this means in practice. For example: how much feature support is needed before a prediction becomes reliable, how performance changes for pairs with sparse evidence,

what fraction of the proteome is covered well enough for confident use, and how medium-confidence predictions should be interpreted. These questions are important for evaluating the real utility of the method from a user's perspective.

Thank you for this suggestion. We now show (Supplementary Figure 4B) the relationship between our top most predictive features and their prevalence in varying levels of confident predictions. The figure shows the top features are more likely in higher confident pairs than low confident pairs. It is important to remember that multiple lines of limited evidence may give rise to confident predictions. Additionally, the feature types used are considerably different in their construction, confidence, and prevalence, for example, the AP-MS-based features are generally highly predictive and present for many pairs.

To address the question of how performance changes for pairs with sparse evidence, we plotted density estimate curves for protein pairs with varying degrees of evidence (Supplementary Figure 4C). In particular, we see when a pair is supported by all feature types, it is more confidently predicted. Further, when a pair has less evidence support, it scores lower.

Regarding the proteome covered in the DirectContacts2 network, in our confident predictions (>=0.7) we identify a total of 10,385 proteins or 52% of the proteome. Additionally, we see a total of 7,164 proteins or 36% of the proteome in our high-confidence network (>=0.9).

While we suggest two score thresholds for our network, certain applications may want to use less confident predictions. We now provide a score vs precision plot (Supplementary Figure 2F) that allows one to evaluate the precision at any confidence score.

10. While DirectContacts2 shows a higher fraction of overlap with cross-linked pairs (127/1,418 vs 123/2,239), the absolute improvement is modest (~3.5 percentage points), yet corresponds to a disproportionately large increase in Z-score (20.5 vs 13.7). This suggests that the null distribution has very low variance, inflating statistical significance and potentially overstating the magnitude of improvement.

As the reviewer correctly notes, DirectContacts2 increases cross-linked protein pair enrichment by ~3.5 percentage points over the previous hu.MAP3.0 model. However, since XL-MS-supported protein pairs are sparse and the two networks contain different numbers of predictions within the evaluated protein-pair set, the more relevant comparison becomes the fold increase of XL-MS support among predicted pairs. DirectContacts2 identifies 127 XL-MS-supported pairs among its 1,418 predictions, whereas hu.MAP3.0 identifies only 123 among its 2,239 predictions, corresponding to hit rates of ~9.0% and ~5.5%, respectively. DirectContacts2 achieves a roughly 1.6-fold higher recovery rate of cross-linked pairs, despite making substantially fewer predictions.

The Z-score comparison is consistent with this effect: DirectContacts2 shows a ~1.5-fold higher enrichment over its matched random background than hu.MAP3.0 (20.5 versus 13.7), indicating that the Z-scores are in fact slightly conservative relative to observed improvement. We agree

that Z-scores should not be interpreted as absolute effect sizes and have therefore clarified the text to report both the absolute overlap as well as the fold improvement in XL-MS-supported prediction density. Taken together, these indicate that DirectContacts2 is not merely statistically significant against a low-variance null, but more concentrated for independently observed cross-linked proteins compared to the previous hu.MAP3.0 model.

11. There is also a minor consistency issue with the score thresholds. Earlier sections define 0.7 and 0.9 as the lower- and higher-confidence cutoffs, but later in the “Intermolecular Cross-links are enriched in DirectContact2 Network” section, TMED5-TMED10 is discussed as a predicted direct interaction despite having a DirectContacts2 score of 0.64. This should be clarified.

Thank you for bringing this to our attention. We now state that this protein pair is “predicted to directly interact near our confident cutoff.” This highlights the case where crosslinking is an orthogonal source of validation in cases where confidence is just below suggested threshold and where one can use additional data in combination with DC2 to improve confidence.

12. Some of the figures are still underdeveloped for a paper at this level. Fig. 2 in particular remains rather thin as a main figure, and the inset bar plot is largely redundant with the PR curves.

We thank the reviewer for this comment. The inset bar plot was a result of a previous reviewer’s comment to combine the figures. We find the bar plot of AUPRC gives the reader a straightforward view of the head-to-head performance of each network. Further, in regards to Figure 2 as a whole, we have now added an additional performance analysis of DirectContacts2 and RoseTTAFold2-PPI in Figure 2B.

Reviewer #3 (Remarks to the Author):

The revision addresses the comments on the original version.

Reviewer #4 (Remarks to the Author):

I co-reviewed this manuscript with one of the reviewers who provided the listed reports. This is part of the Nature Communications initiative to facilitate training in peer review and to provide appropriate recognition for Early Career Researchers who co-review manuscripts.

Reviewer #5 (Remarks to the Author):

I co-reviewed this manuscript with one of the reviewers who provided the listed reports. This is part of the Nature Communications initiative to facilitate training in peer review and to provide appropriate recognition for Early Career Researchers who co-review manuscripts.
