# Supplementary material for: DirectContacts2: a wiring diagram of human physical protein interactions
Source: Nat Commun. 2026 Jul 24;17:9037. doi: 10.1038/s41467-026-75863-3 (PMC13507227; doi:10.1038/s41467-026-75863-3)
Supplement: Supplementary file 3 [file 41467_2026_75863_MOESM1_ESM.pdf]

**Supplementary Figures**

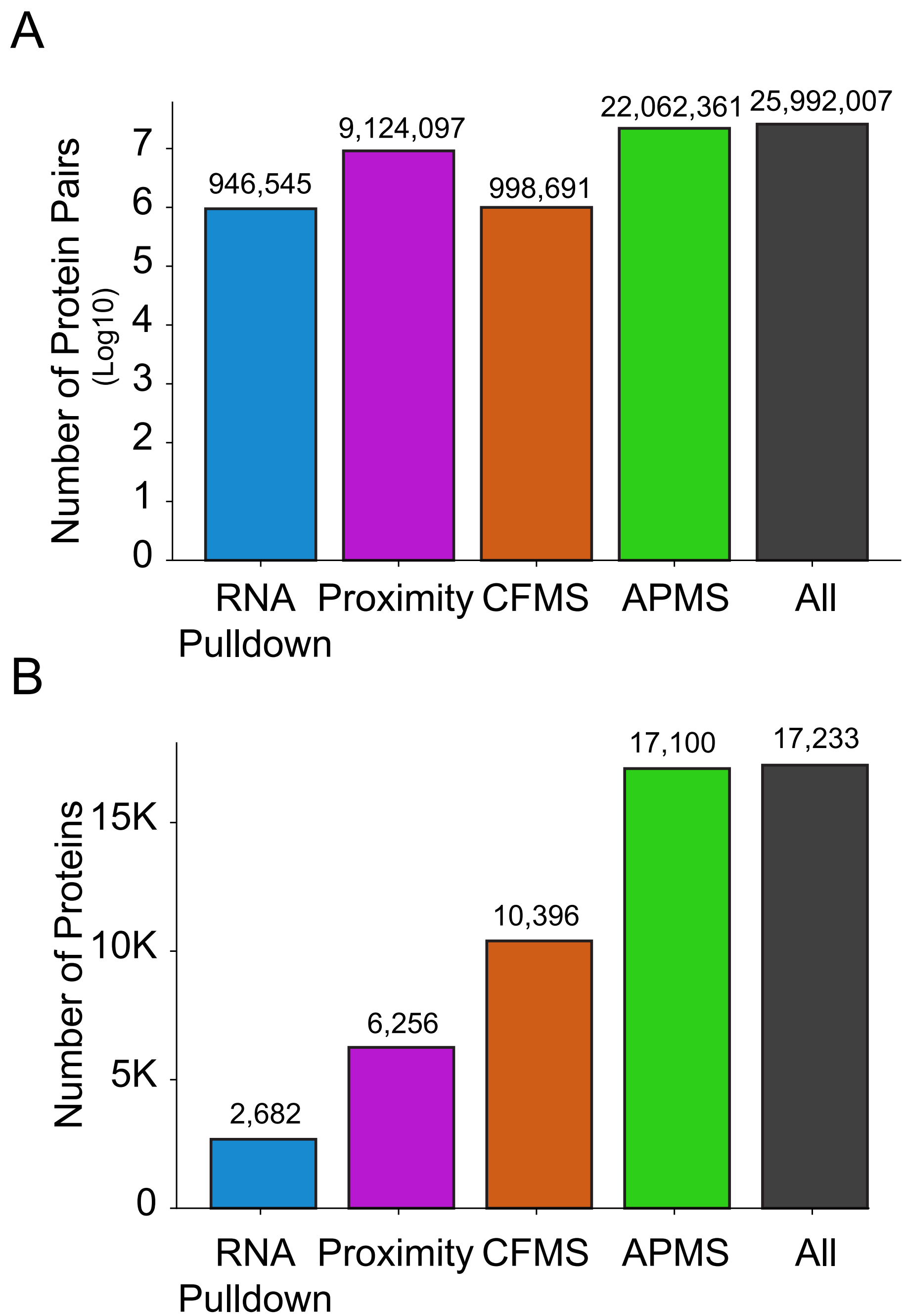


**Supplementary Figure 1: Proteomics dataset coverage of DirectContacts2. A**. Coverage of each proteomic experimental evidence type for human protein pairs. **B.** Coverage of each proteomic experimental evidence type for human proteins.

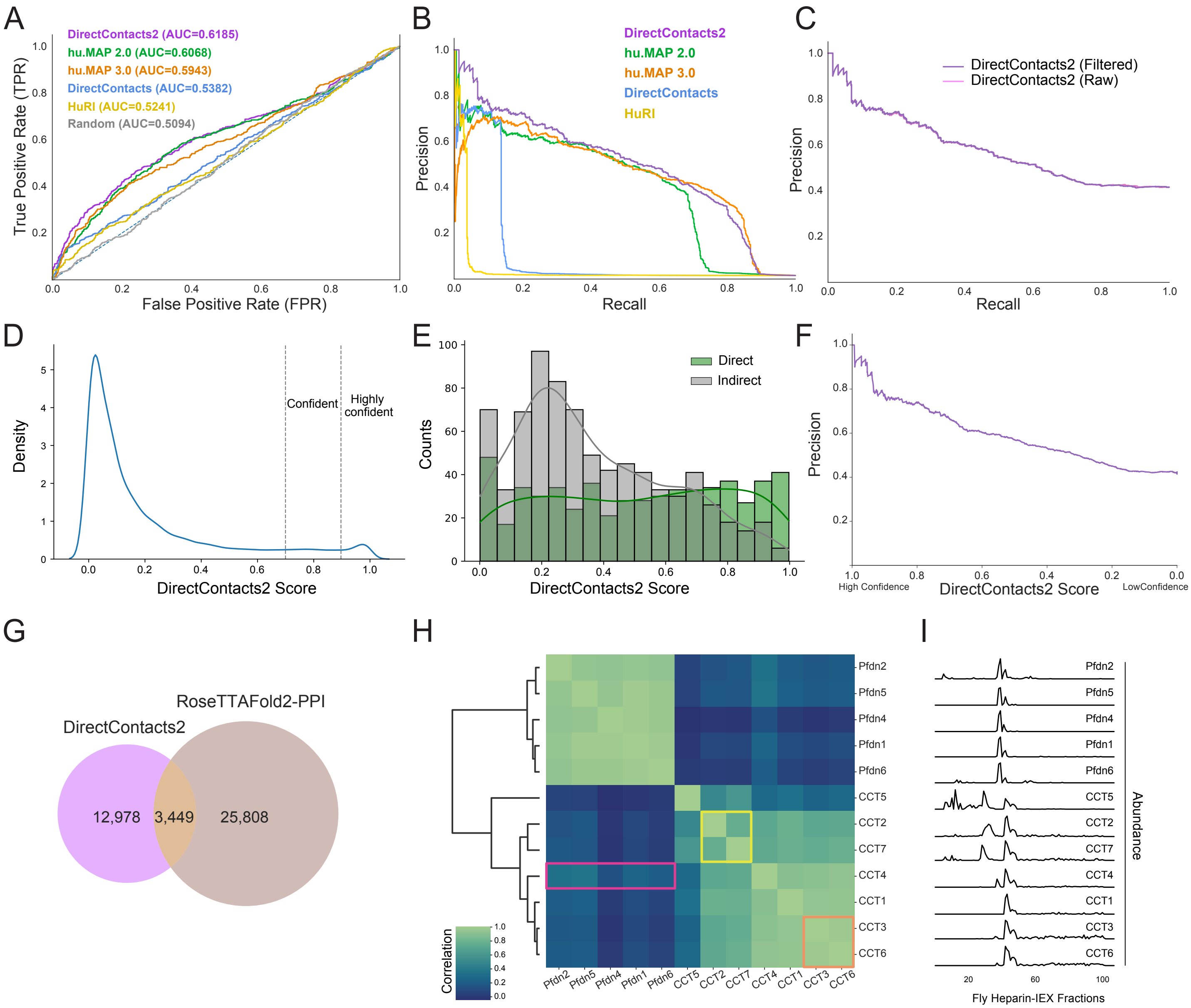


**Supplementary Figure 2: Evaluation of DirectContacts2 network A.** Receiver operator curve of unfiltered DirectContacts2 network plotted with other models evaluated on test set with intra-complex negative labels only. **B**. Precision recall analysis with expanded set of inter-complex negative labels for the unfiltered DirectContacts2 network against other models. **C**. Precision-recall curve of DirectContacts2 network on test benchmark with negatives restricted to intra-complex pairs pre (pink) and post (purple) filtering pairs found in known or predicted complexes. **D.** Distribution of DirectContacts2 scores for predicted pairs post filtering by complexes with confidence thresholds indicated. **E**. Distribution of DirectContacts2 scores for true direct interactions (green) and false indirect interactions (gray) in the leave-out test set are significantly different (Mann Whitney two-sided test p-value: 4e-18). **F.** Precision vs confidence score plot. **G**. Venn diagram of confident DirectContact2 predictions (score >= 0.7) and RoseTTAFold2-PPI network (80% expected precision). **H**. Heatmap of Pearson correlation coefficients between CCT/Prefoldin complex subunit protein abundance profiles from a selected Heparin-IEX fractionation in *D. melanogaster.* Proteins are hierarchically clustered along both axes. The yellow (CCT2 – CCT7) and orange (CCT3 – CCT6) boxes highlight high correlation among direct interactions of the CCT complex. The magenta box highlights the highest correlation of a CCT subunit (i.e. CCT4) with subunits of the Prefoldin subcomplex. **I**. Example co-fractionation data (normalized spectral counts) for the same experiment clustered in panel **H**. Subunits for the CCT/Prefoldin complexes show

characteristic co-elution. Directly interacting subunits (i.e. CCT2 – CCT7 and CCT3 – CCT6) display the most similar pairs to other CCT subunit elution profiles. Source data are provided as a Source Data file.

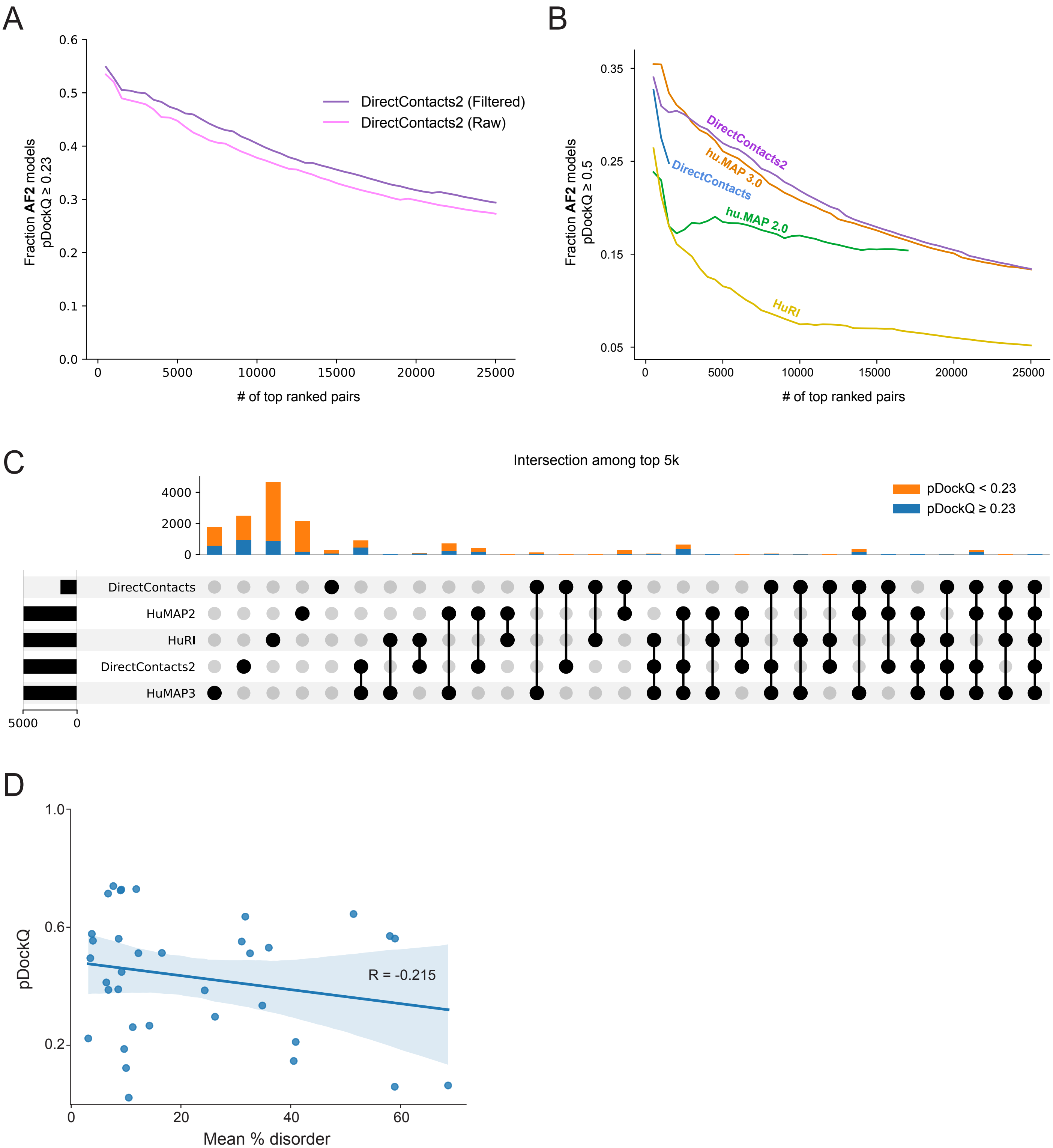


**Supplementary Figure 3: DirectContacts2 is enriched with confident AlphaFold models A**. Comparison of enrichment for confident pair interfaces (pDockQ ≥ 0.23) out of the top confident pairs in pre and post complex filtered DirectContacts2 networks. **B**. Comparison of enrichment for highly-confident pair interfaces (pDockQ ≥ 0.5) out of the top confident pairs in DirectContacts2 and other networks. **C**. UpSet plot showing the overlap among the top 5,000 most confident predicted pairs from each network with the fraction of pairs with confident pDockQ scores (≥ 0.23) indicated in blue. **D**. Regression plot showing negative Pearson correlation (R = -0.215) between pDockQ and mean disorder percentage of protein pairs. Each point represents a

pairwise AlphaFold3 model, for which pDockQ scores were calculated and disorder annotations were obtained from DisProt[67]. Source data are provided as a Source Data file.

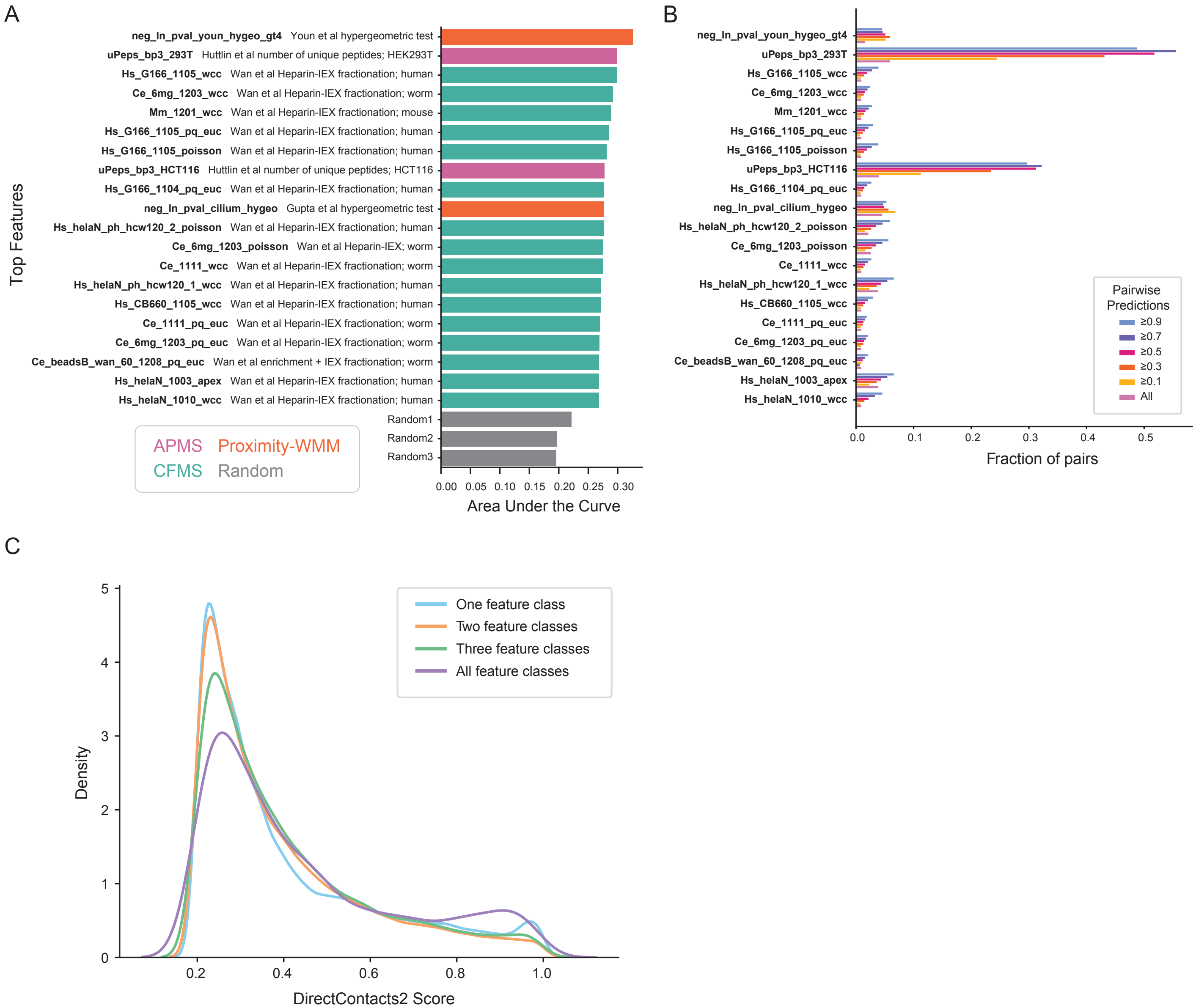


**Supplementary Figure 4: Evaluation of DirectContacts2 features to discriminate between direct and indirect interactions. A.** Bar plot shows the area under the precision recall curve for untrained individual features evaluated on a training set excluding inter-complex negative edges. CFMS features are shown in green, APMS features are shown in pink, and Proximity-WMM features are shown in orange. Three iterations of randomly shuffled benchmark pairs are also evaluated (gray). See Supplementary Data 11 for full feature descriptions. **B**. Grouped bar plot showing the fraction of pairs that have high-scoring values (top 25%) for each high-ranking feature from **A**. Fractions of pairs are shown for different sets of DirectContacts2 confidence thresholds. **C**. Density plot that shows the relationship of the DirectContacts2 score and the number of feature classes present for a pair. The set of pairs includes those which have a DirectContacts2 score ≥ 0.2 (see Methods). Source data are provided as a Source Data file.

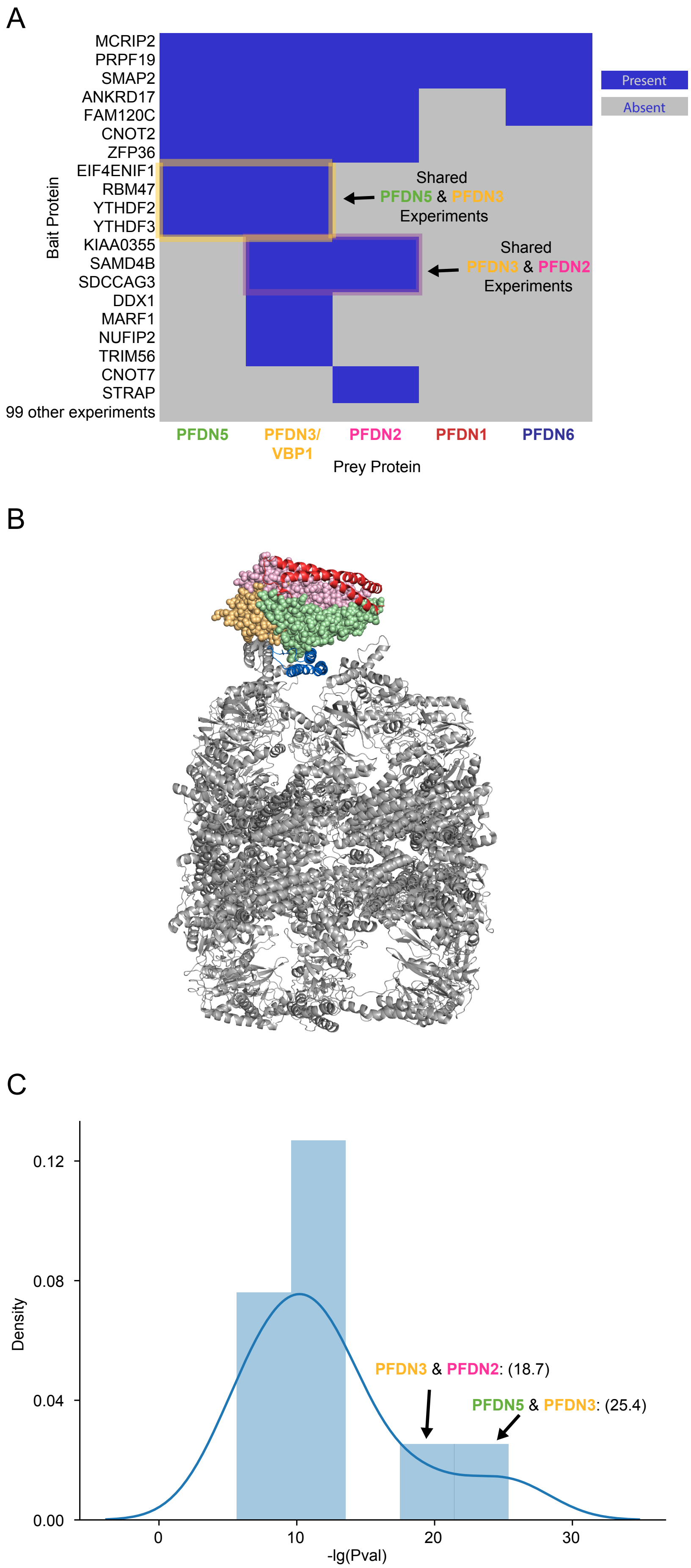
A
MCRIP2
PRPF19
SMAP2
ANKRD17
FAM120C
CNOT2
ZFP36
EIF4ENIF1
RBM47
YTHDF2
YTHDF3
KIAA0355
SAMD4B
SDCCAG3
DDX1
MARF1
NUFIP2
TRIM56
CNOT7
STRAP
99 other experiments
Bait Protein
Present
Absent
Shared PFDN5 & PFDN3 Experiments
Shared PFDN3 & PFDN2 Experiments
PFDN5
PFDN3/ VBP1
PFDN2
PFDN1
PFDN6
Prey Protein
B
C
0.12
0.08
0.04
0.00
Density
0
10
20
30
-lg(Pval)
PFDN3 & PFDN2: (18.7)
PFDN5 & PFDN3: (25.4)

**Supplementary Figure 5: Weighted Matrix Model applied to proximity labeling experiments provides evidence for direct interactions in CCT/Prefoldin complex. A.** Heatmap of proximity labeling experiments which identify prefoldin subunits. Directly interacting pairs (PFDN5-PFDN3 (yellow box) and PFDN3-PFDN2 (purple box)) are seen together in the same experiments. PFDN4 was excluded because it did not pass the abundance threshold of ≥ 4 peptide spectral matches. **B.** PDB structure of CCT/Prefoldin complex showing PFDN5-PFDN3 and PFDN3-PFDN2 directly interact. CCT subunits in gray, PFDN5 (green), PFDN3 (orange), PFDN2 (pink), PFDN1 (red), PFDN6 (blue). **C.** Distribution of WMM calculated -lg(pval) of prefoldin subunit pairs shows bimodal distribution. Directly interacting pairs PFDN3-PFDN2 and PFDN5-PFDN3 are right shifted with higher WMM values. Source data are provided as a Source Data file.

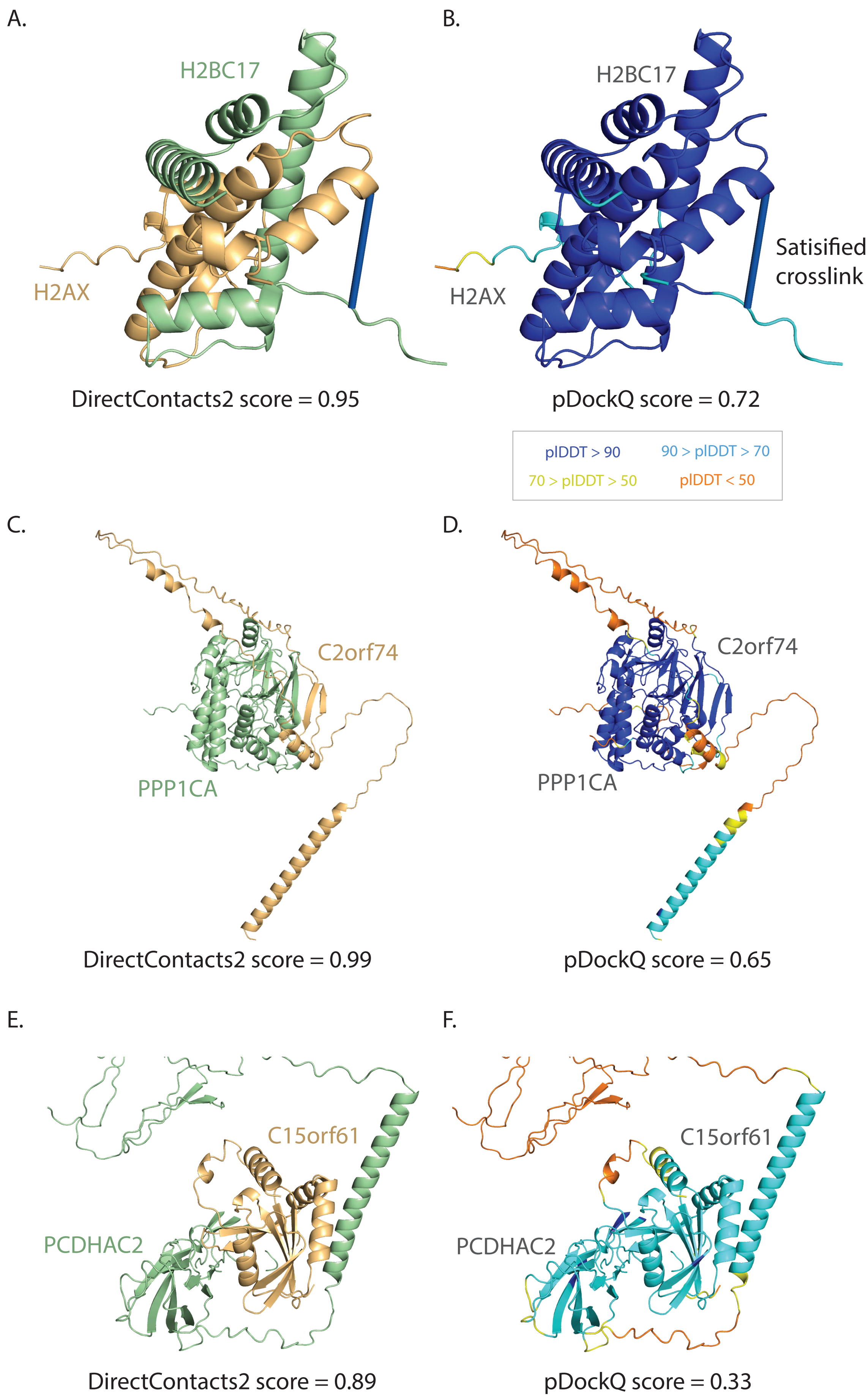

A.
H2BC17
H2AX
DirectContacts2 score = 0.95
B.
H2BC17
H2AX
Satisified
crosslink
pDockQ score = 0.72
pIDDT > 90
90 > pIDDT > 70
70 > pIDDT > 50
pIDDT < 50
C.
C2orf74
PPP1CA
DirectContacts2 score = 0.99
D.
C2orf74
PPP1CA
pDockQ score = 0.65
E.
C15orf61
PCDHAC2
DirectContacts2 score = 0.89
F.
C15orf61
PCDHAC2
pDockQ score = 0.33

**Supplementary Figure 6: Structural modeling of interactions with uncharacterized proteins. A.** AlphaFold3 model of H2AX (orange) and H2BC17 (green) interaction. Blue line represents satisfied crosslink. **B.** Same model in **A,** colored by AlphaFold3 pLDDT. **C.** AlphaFold3 model of C2orf74 (orange) and PPP1CA (green) interaction. **D.** Same model in **C,** colored by AlphaFold3 pLDDT. **E.** AlphaFold3 model of C15orf61 (orange) and PCDHAC2 (green) interaction. **F.** Same model in **E,** colored by AlphaFold3 pLDDT.

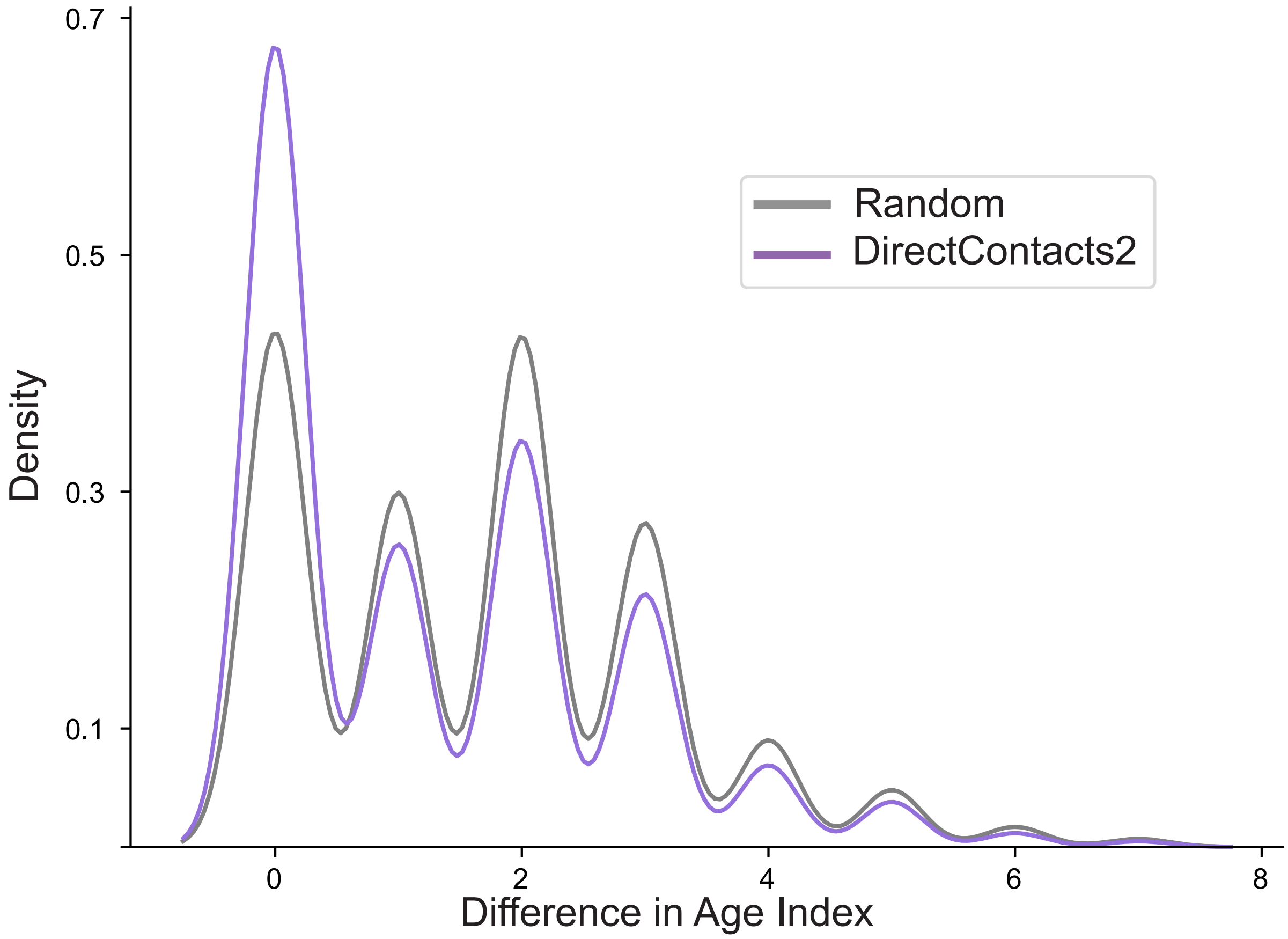


**Supplementary Figure 7: Evolutionary ages of directly interacting proteins are more similar than random.** Kernel density estimation for the distribution of pairwise differences in evolutionary age index for confident DirectContacts2-predicted interactions compared to randomly shuffled pairs (n=6,844). Lower values indicate greater similarity of inferred evolutionary age between proteins in a pair, while higher values reflect greater age disparities. Source data are provided as a Source Data file.

**Supplementary Note 1: Benchmark clustering algorithm pseudocode**

```
def cluster_complexes(complexes):
    C <- complexes
    clusters <- [ ]

    jaccard_df <- init_dataframe(|C|, |C|) # |C| is the number of complexes
    #calculate jaccard index for all pairs of complexes
    for i, ci in C:
        for j, cj in C:
            jaccard_df[i,j] <- |ci ∩ cj| / |ci ∪ cj|

    while |C| > 0:
        #reorder the jaccard dataframe based on hierarchical clustering
        ordered_complexes <- hierarchical_cluster_order(jaccard_df)
        anchor <- ordered_complexes[0]

        #find index of first nonoverlapping complex in ordered_complexes
        h <- first position k s.t. Jaccard(anchor, ordered_complexes[k]) = 0
        seed <- ordered_complexes[0 : h]

        while True:
            add_complexes <- [ ] #initialize empty list of complexes to add to seed
            for i, c in C: # i is the index of complex c in set of complexes C
                for j, s in seed: # j is the index of complex s in seed cluster
                    if jaccard_df[i,j] > 0:
                        add_complexes.append(c)

            if |add_complexes| == 0:
                #no new complexes were added
                break
            else:
                seed.append(add_complexes)

        clusters.extend(seed) #add finalized cluster to list of clusters
        C.remove(seed) #remove all complexes in seed from C
        jaccard_df.remove(seed) #remove all complexes in seed from jaccard matrix

    return cluster
```
