# Supplementary material for: DirectContacts2: a wiring diagram of human physical protein interactions
Source: Nat Commun. 2026 Jul 24;17:9037. doi: 10.1038/s41467-026-75863-3 (PMC13507227; doi:10.1038/s41467-026-75863-3)
Supplement: Supplementary file 4 [file 41467_2026_75863_MOESM2_ESM.pdf]

Description of Additional Supplementary Files

Filename: Supplementary Data 1
Description: Benchmark Train Positive Pairs. This file contains the positive protein pairs from the benchmark training set. The pairs are listed as two proteins (as UniProt accessions) per line.

Filename: Supplementary Data 2
Description: Benchmark Train Intra- and Inter-Complex Negative Pairs. This file contains the negative protein pairs from the benchmark training set, which includes indirect interactions from within a complex (i.e., intra-complex negative pairs) and interactions between complexes (i.e., inter-complex negative pairs). The pairs are listed as two proteins (as UniProt accessions) per line.

Filename: Supplementary Data 3
Description: Benchmark Test Positive Pairs. This file contains the positive protein pairs from the benchmark test set. The pairs are listed as two proteins (as UniProt accessions) per line.

Filename: Supplementary Data 4
Description: Benchmark Test Intra-Complex Negative Pairs. This file contains the negative protein pairs from the benchmark test set that only includes indirect interactions from within a complex (i.e., intra-complex negative pairs). The pairs are listed as two proteins (as UniProt accessions) per line.

Filename: Supplementary Data 5
Description: Benchmark Test Intra- and Inter-Complex Negative Pairs. This file contains the negative protein pairs from the benchmark test set, which includes indirect interactions from within a complex (i.e., intra-complex negative pairs) and interactions

between complexes (i.e., inter-complex negative pairs). The pairs are listed as two proteins (as UniProt accessions) per line.

Filename: Supplementary Data 6
Description: Benchmark Positive Pairs with PDBs. This file contains positive pairs from the benchmark, their benchmark split (e.g., test or train), and the PDB that the protein pair is associated with.

Filename: Supplementary Data 7
Description: AutoGluon leaderboard of models. This file contains the performance comparison of the 13 base models and one ensemble model from AutoGluon.

Filename: Supplementary Data 8
Description: All 25 million predictions. This file contains the protein identifiers (UniProt accessions) for each pair and their DirectContacts2 score

Filename: Supplementary Data 9
Description: DirectContacts2 Complex Filtered Network (unthresholded). This file contains all the protein pairs (as UniProt accessions) from the final complex-filtered network, their DirectContacts2 scores, and the complex identifier that they are associated with. There is no threshold on the DirectContacts2 score and therefore the confidence group that each pair belongs to is listed.

Filename: Supplementary Data 10
Description: AlphaFold3 model prediction metrics. This file contains the protein pairs (as UniProt accessions) for all AlphaFold3 predictions made in this study, their DirectContacs2 scores, their ipTM scores, and their pDockQ scores.

Filename: Supplementary Data 11
Description: Detailed description of machine learning features. This file contains descriptions for every feature in the feature matrix, including: the feature type,

experimental information (e.g., cell type, type of biochemical fractionation), and whether the feature is one of the Top 20 features.

Filename: Supplementary Data 12

Description: Evolutionary age of DirectContacts2 interactions. This file contains the protein pairs (as UniProt accessions) analyzed for their evolutionary age, their DirectContacts2 scores, their individual evolutionary ages and differences, the identifiers of the associated complexes to each pair, and their average complex difference.
